# Novel exon combinations generated by alternative splicing of gene fragments mobilized by a CACTA transposon in *Glycine max*

**DOI:** 10.1186/1471-2229-7-38

**Published:** 2007-07-14

**Authors:** Gracia Zabala, Lila Vodkin

**Affiliations:** 1 Department of Crop Sciences, University of Illinois, Urbana, Illinois 61801, USA

## Abstract

**Background:**

The recent discoveries of transposable elements carrying host gene fragments such as the Pack-MULEs (Mutator-like transposable elements) of maize (*Zea mays*), rice (*Oryza sativa*) and *Arabidopsis thaliana*, the *Helitrons *of maize and the *Tgm-Express *of soybeans, revealed a widespread genetic mechanism with the potential to rearrange genomes and create novel chimeric genes affecting genomic and proteomic diversity. Not much is known with regard to the mechanisms of gene fragment capture by those transposon elements or the expression of the captured host gene fragments. There is some evidence that chimeric transcripts can be assembled and exist in EST collections.

**Results:**

We report results obtained from analysis of RT-PCR derived cDNAs of the *Glycine max *mutant flower color gene, *wp*, that contains a 5.7-kb transposon (*Tgm-Express1*) in Intron 2 of the flavanone 3-hydroxylase gene (*F3H*) and is composed of five unrelated host gene fragments. The collection of cDNAs derived from the *wp *allele represents a multiplicity of processed RNAs varying in length and sequence that includes some identical to the correctly processed mature F3H transcript with three properly spliced exons. Surprisingly, the five gene fragments carried by the *Tgm-Express1 *were processed through complex alternative splicing as additional exons of the *wp *transcript.

**Conclusion:**

The gene fragments carried by the *Tgm *inverted repeat ends appear to be retained as functional exons/introns within the element. The spliceosomes then select indiscriminately the canonical intron splice sites from a pre-mRNA to assemble diverse chimeric transcripts from the exons contained in the *wp *allele. The multiplicity and randomness of these events provide some insights into the origin and mechanism of alternatively spliced genes.

## Background

**Figure 1:**
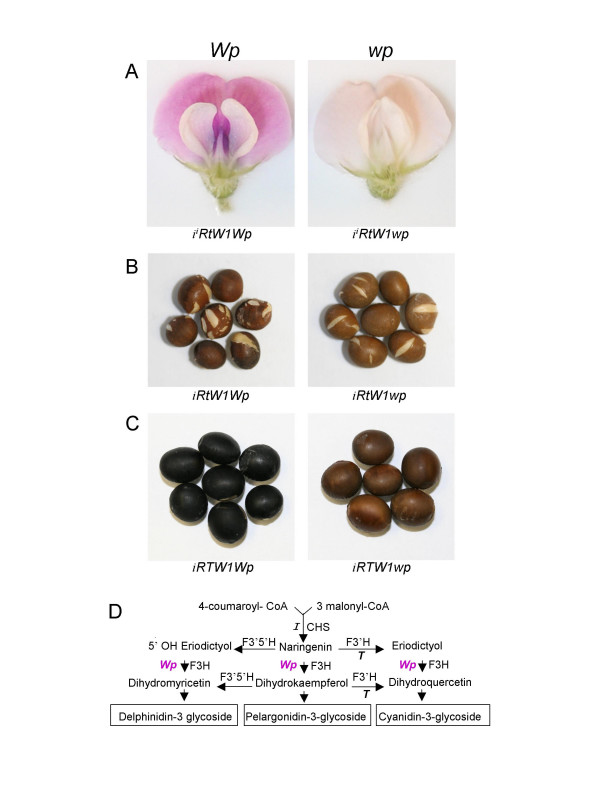
**Illustration of the effect of *wp *on flower and seed coat phenotypes**. **(A) **Stable purple flower of plants with *Wp *genotype (left panel) or stable pink flower of plants with *wp *genotype (right panel) in lines LN89-5320-6 (*i*^*i*^*RtW1Wp) and *LN89-5322-2 (*i*^*i*^*RtW1wp*) both of which have yellow seed coats. In soybean *I *(*CHS*), *R *and *T *(*F3'H*) are three independent loci that control pigmentation in seed coats and *W1 *(*F5'3'H*) and *Wp *(*F3H*) were described as flower color markers, but all five loci seem to be encoding genes of the anthocyanin and proanthocyanidin pathways. Mutant alleles of those loci (*i, i*^*i*^, *r, t, w1 *and *wp*) affect flower, seed coat, hypocotyle or pubescence coloration [1, 28, 30, 31]. **(B) **Imperfect black color of seed coats of plants with *iRtW1Wp *genotype (left panel) as contrasted with the lighter shaded seed coats of plants with *iRtW1wp *genotype (right panel). Effect on the seed coat phenotype was revealed by crossing the *wp *allele into lines having the recessive *i *allele that allows spatial pigmentation of the entire seed coat [24]. The cracks on both seed coat types result from an epistatic effect of *t *[31]. **(C) **Black seed coats of plants with *iRTW1Wp *genotype where the *T *allele drives the synthesis of cyanidins (left panel) contrasted with the lighter seed coats of plants with *iRTW1wp *genotype (right panel). **(D) **Abbreviated schematic representation of the three branches leading to the synthesis of the three anthocyanin classes and the genes encoding the enzymes relevant to the present study.

A mutation in a soybean flower color gene *(Wp) *encoding a flavanone 3-hydroxylase (F3H) was characterized as a novel transposon insertion, *Tgm-Express1*, of the CACTA superfamily, that carried multiple captured host gene fragments [1]. The most visible effect of the *wp *mutation is production of pink rather than purple flowers and lighter color in the seed coats (Figure 1). It has also been associated with lower oil and higher seed protein content than the purple flowered *Wp *isoline [2,3].

The *Tgm-Express1 *element, like the Pack-MULEs (Mutator-like elements) of maize, rice and *Arabidopsis*, retroelements in rice and the *Helitrons *of maize contains several host gene fragments. It carries intronic and exonic regions of five genes: unknown protein (*UP*), cell division cycle 2 (*CDC2*), fructose-6-phosphate 2-kinase/fructose-2-6-biphosphatase (*FPK*), malate dehydrogenase (*M*) and cysteine synthase (*CS*) [1]. Little is known about how any of those transposons or retroelements acquired the gene pieces but there is some evidence that they are transcribed creating chimeric cDNAs that exist in EST (expressed sequence tag) collections [4-7]. Of 475 Pack-MULEs identified on chromosomes 1 and 10 of rice via computer searches, 5% were transcribed based on exact matches to full-length cDNAs [4]. Most of the transcripts (91%) were initiated from promoters at the TIRs (terminal inverted repeats) or within the element while 9% of the transcripts initiated outside the element. Three chimeric transcripts were seen in an RNA blot probed with both a *sh2 *and a *Helitron *insertion fragment probes [8]. A single 2,620 bp chimeric transcript spanning the entire *Helitron *including several gene fragments contained within the element has also been described [6]. The promoter was predicted to reside upstream of the *Helitron *insertion site.

We present here an array of 12 distinct alternatively spliced chimeric transcripts obtained via RT-PCR that were derived from the soybean *wp *mutant allele in which the second intron of the *F3H1 *gene is interrupted by the 5.7 kb transposon containing five captured host gene-fragments. The chimeric transcripts analyzed were more abundant in seed coats than in cotyledons and ranged in size from 3,108 bp to a correctly processed one of 1,422 bp that was identical to the transcript derived from the wild type *Wp *allele. All transcripts isolated initiated at the *F3H1 *gene (*Wp*, *wp*) promoter that is strongly expressed in seed coats.

Alternative splicing is a common regulatory mechanism in higher eukaryotes and the mechanisms governing it have been studied extensively in mammalian systems but sparingly in plants [9,10]. In general, the splicing pattern of a multiexon pre-mRNA can be altered in many ways. Exons that are always spliced and included in the mature mRNA are known as constitutive exons. However, mechanistic decisions of the splicing components can result in exons that are included at times but excluded at others times from the mature mRNA. These are referred to as cassette exons. There are also occurrences of 5' and 3' alternative splice sites altering the length of some exons. In addition, the failure to remove an intron, referred to as intron retention, is also found. Genes whose pre-mRNAs have multiple locations of alternative splicing produce a family of related proteins with different allosteric regulation, protein localization, or enzymatic activity [9].

We show that the exon/intron regions of gene fragments carried by the *Tgm-Express1 *of the *wp *allele are alternatively spliced and assembled with the constitutive exons of the *F3H1 *gene to generate an array of chimeric transcripts encoding a variety of open reading frames (orfs). Analysis of the derived amino acid sequence from the 12 distinct *wp *chimeric cDNAs predicted putative chimeric orfs varying in length and frame locations.

The splicing machinery at times eliminates all extraneous (cassette) exons and introns of the *Tgm-Express1 *element to generate a full length transcript identical to that of the wild type gene and thus likely functional. The number of F3H molecules may be extensive enough to allow the synthesis of sufficient anthocyanin pigment that could account for the pink flower and the lighter seed coat phenotypes in the *wp *lines (Figure 1). On the other hand, the more complex chimeric transcripts containing cassette exons (UP, CDC2, FPK, M and CS) from the captured gene fragments in the transposon may upon translation generate products that could interfere with function of the wild type host-gene counterparts leading to secondary phenotypes. Whether any of the novel exon combinations derived from alternative splicing of the mobile exons of the *Tgm-Express1 *element create new phenotypes is unknown. However, there is growing evidence from both plant [11,7] and animal [12,13] systems that repeat sequences derived from mobile elements play a significant role in generation and evolution of novel genes and exons. The *wp *locus in soybean is a unique example of an insertional mutation in the act of *de novo *generation of fused, multiple chimeric exons through inclusion or exclusion of cassette exons carried by the element into the affected gene.

## Results

### Complex aberrant expression of the flower color mutant gene wp

We discovered that a pink flower locus (*Wp*) of soybean encoded a flavanone 3- hydroxylase gene (*F3H1*) by differential screening of a cDNA soybean microarray using RNAs from mutant pink (*wp*) and standard purple (*Wp*) flower isolines [1]. We also showed that the *Tgm-Express1 *transposon insertion impaired expression of the mutated gene and that the *F3H1 *gene was strongly expressed in the seed coats but not in cotyledons [1].

**Figure 2:**
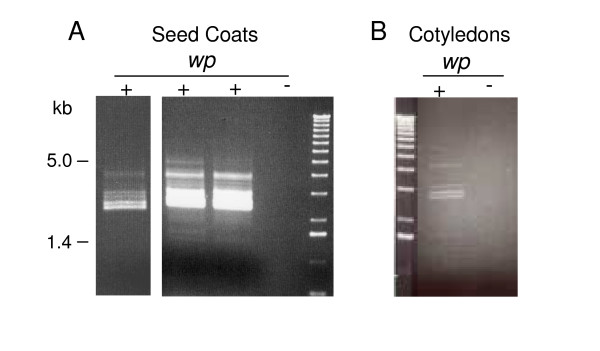
**Variant flavanone 3-hydroxylase cDNAs from isolines containing mutant *wp *alleles. (A) **Ethidium bromide-stained gel showing an array of cDNA bands between 5 and 1.4 kb in size that were amplified from RNAs of seed coats of the *wp *mutant line LN89-5322-2 through RT-PCR reactions. The (+) and (-) at top indicate reactions with and without Superscript RTII. The bright broad bands obtained from mutant RNA samples in the (+) reactions were resolved into a group of discreet bands with shorter photographic exposure of the same gel (far left lane). **(B) **Ethidium bromide-stained gel showing cDNAs amplified via RT-PCR with RNA from cotyledons of the LN89-5322-2 (*wp*) mutant line.

Analysis of the *wp *allele expression by RT-PCR with a pair of *F3H1 *outermost 5' and 3'-primers revealed a bizarre pattern of amplification resulting in a variety of cDNA sizes from both seed coat and cotyledon RNAs (Figure 2). The broad bright band of PCR product obtained with the seed coat samples (Figure 2A) represents multiple size bands. Shorter exposure photograph of that same gel revealed at least 4 distinct bands (left most lane, Figure 2A). The *wp *transcriptional activity between the two tissues, cotyledons and seed coats, could be deduced from the difference in the intensity of the PCR products obtained from the two *wp *RNA sets (Figure 2A and 2B). Even though no hybridization to a F3H probe was apparent by RNA blots with cotyledon RNAs of either genotype (*Wp *and *wp*) [1], RT-PCR showed the existence of 1.4 kb transcripts representing the mature *F3H1 *gene (data not shown) and the aberrant larger transcripts from mutant line RNAs (Figure 2B).

**Figure 3:**
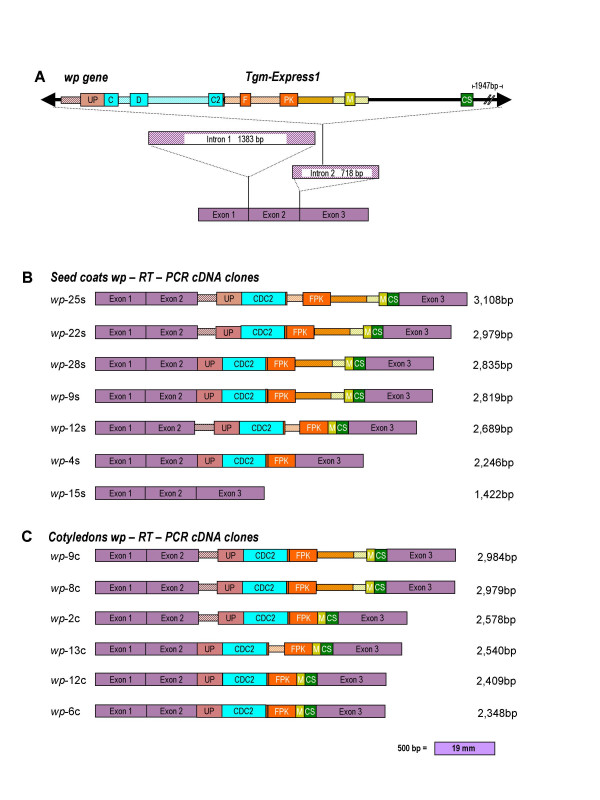
**Schematic representation of the *wp *recessive allele and the novel exon combinations generated in its transcribed RNAs. (A) **Represents the genomic sequence of the mutant *wp *allele obtained from the line LN89-5322-2 (*i*^*i*^*RtW1wp*). The introns are indicated and their length given in bp. The 5,725 bp *Tgm-Express *insertion in Intron 2 is drawn at top with the arrow heads representing inverted repeats and the five captured gene fragments color coded. The full length of the mutant gene is 9,251 bp. The three Exons in purple represent the cDNA of the proper spliced wild type gene 1,422 bp in size. The 7F and 1428R primers used in the PCR reactions that generated the chimeric cDNA clones shown in Figure 3B and C map at the 5' end of Exon 1 (7F) and the 3' end of Exon 3 (1428R) respectively. **(B) **Graphic representation of six chimeric, multi-exon cDNA clones (*wp*-25s, -22s, -28s, -9s, 12s, -4s) derived from seed coat RNAs of the *wp *mutant line via RT-PCR. These clones contained besides the F3H three Exons (1, 2, 3) varying numbers of alternatively spliced exons (solid color boxes) and introns (dashed narrower boxes) from 3 or 5 of the *Tgm-Express1 *captured gene fragments (*UP, CDC2, FPK, M *and *CS*). A seventh cDNA clone, *wp*-15s, also derived from the mutant *wp *line is composed only of the wild type gene (*Wp*) Exons 1, 2 and 3. **(C) **Six chimeric cDNA clones (*wp*-9c, -8c, -2c, -13c, -12c, -6c) derived from cotyledon RNAs of the *wp *mutant line via RT-PCR. All clones contained the F3H Exons (1, 2,3) with varying numbers of alternatively spliced exonic and intronic regions from the *Tgm-Express1 *acquired host-gene fragments separating the Exon 2-Exon 3 junction. Abbreviations: *UP*, unknown protein; *CDC2*, cell division cycle 2; *FPK*, fructose-6-phosphate 2-kinase/fructose-2-6-biphosphatase; *M*, malate dehydrogenase; *CS*, cysteine synthase. Two CDC2 intronic regions captured by the transposon element and sandwished between the three exonic regions (C, D and C2, Figure 2A) were spliced out to form the CDC2 exon in the chimeric transcripts (Figure 2B and C). One FPK intronic fragment captured by the transposon between two flanking exons (F and PK, Figure 2A) was also spliced out to form the FPK exon in the chimeric transcripts (Figure 2B and C). A smaller FPK intron flanked by 15 bp exon fragment (narrow orange block not named) at the 5'end (Figure 2A) is not always spliced out (Figure 2B and C).

Cloning the larger sized RT-PCR cDNAs from plants homozygous for the *wp *allele resulted in a surprising array of alternatively spliced transcripts. Sequence analysis of the multiple size cDNAs cloned from both seed coat and cotyledons revealed multiple transcripts derived from the *wp *allele containing the wild type gene (*F3H1*) exons (1, 2, 3) plus varying portions of exonic and intronic regions of the gene fragments captured by the *Tgm-Express1 *element that interrupts Intron 2 in the *wp *allele (Figure 3).

Figure 3A shows the schematic representation of the genomic sequence of the *wp *allele with the *Tgm-Express1 *insertion in Intron 2. The number of gene fragments contained within the element and their exons (solid colored boxes) and introns (striped boxes) were revealed upon sequence analysis of the multiple transcripts derived from *wp *expression in seed coats (See additional file 1: Seed coat *wp *RT-PCR cDNA sequence alignment) and cotyledons (See additional file 2: Cotyledons *wp *RT-PCR cDNA sequence alignment). There were exonic portions of five distinct genes: unknown protein (*UP*), cell division cycle 2 (*CDC2*), fructose-6-phosphate 2-kinase/fructose-2-6-biphosphatase (*FPK*), malate dehydrogenase (*M*) and cysteine synthase (*CS*). Some of the intronic regions could be assigned to specific genes (one color stripes) while others (two colors) could not. The solid black line between the solid arrow heads (inverted repeats) could be introns or intergenic DNA regions. Including the latter, all marked intronic regions conform to the canonical 5'GT donor and 3'AG acceptor splice sites.

Figure 3B is a graphic summary of seven distinct RT-PCR cDNA clones derived from seed coat RNAs. The larger clones (*wp*-25s, *wp*-22s, *wp*-28s, *wp*-9s and *wp*-12s) contain beside the three exons of the *F3H1 *gene, all cassette exons of the *Tgm-express1 *gene fragments and varying intron pieces. The smaller clones (*wp*-4s and *wp*-15s) had only the F3H exons (*wp*-15s) or the F3H exons and three cassette exons (*wp*-4s). Sequence data from these clones have been deposited with the EMBL/GenBank Data Libraries under accession numbers: EF100865 (*wp-*25s), EF100866 (*wp-*22s), EF100867 (*wp-*28s), EF100868 (*w-p*9s), EF100869 (*wp-*12s), EF100870 (*wp-*4s), EF100871 (*wp*-15s).

Likewise, Figure 3C shows six cDNA clones obtained via RT-PCR from cotyledon RNAs. As in the case of the seed coat derived cDNA clones, the larger cotyledon cDNA clones (*wp*-9c, *wp*-8c, *wp*-2c and *wp*-13c) contained some intron fragments besides the three F3H exons and cassette exons from the *Tgm-Express1 *element. The smaller clones (*wp*-12c and *wp*-6c) contained only exons, the three F3H exons and the five cassette exons correctly spliced. The latter two clones diverged only by 61 bp mostly due to two splicing errors in *wp-*6c deleting 15 bp at the beginning of Exon 2 and 47 bp at the CDC2/FPK exons junction (See additional file 2: Cotyledons *wp *RT-PCR cDNA sequence alignment). Of the six cotyledon cDNAs cloned, only one (*wp*-8c) was identical to one (*wp*-22s) of the seed coat cDNA clones. Sequence data from these clones have been deposited with the EMBL/GenBank Data Libraries under accession numbers: EF100872 (*wp*-9c), EF100873 (*wp*-8c), EF100874 (*wp*-2c), EF100875 (*wp*-13c), EF100876 (*wp*-12c), and EF100877 (*wp*-6c).

Overall, we isolated 12 different transcripts synthesized from the *wp *allele. These are a good representation of the chimeric transcripts generated by the spliceosome machinery in the tissues examined. We conclude that the most abundant transcripts shown by the discrete bands in figure 2A (left lane) have been cloned based on their size. The four most abundant bands are between 2 and 3 kb in size as are 11 of the 12 different cloned cDNAs. Our results also demonstrated that alternative splicing at the *wp *allele occurs in two tissues, one (the seed coats) in which the F3H promoter is highly expressed and another (cotyledons) in which it is not.

### Open reading frames of chimeric, multi-exon wp transcripts

**Figure 4:**
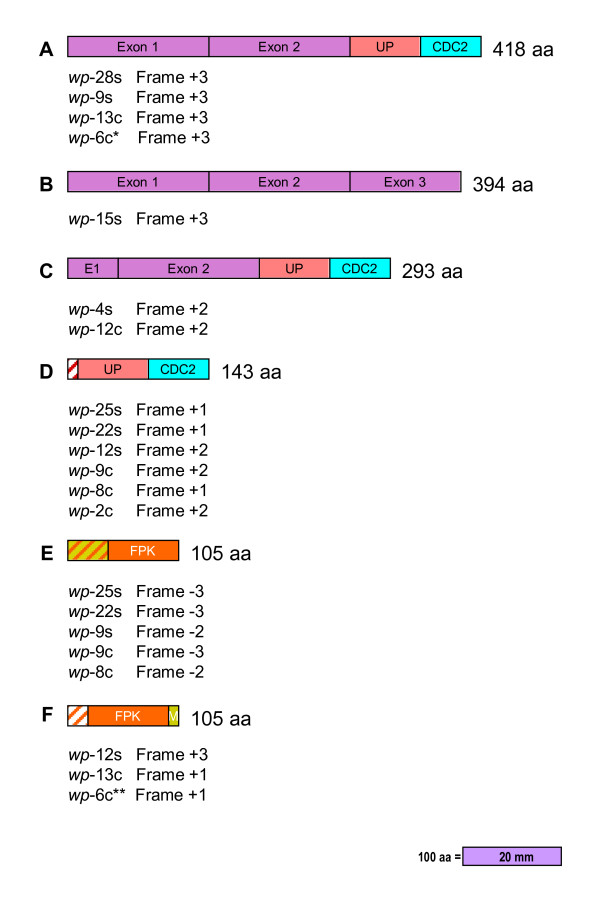
**Schematic of relevant chimeric and non-chimeric orfs generated by the *wp *allele**. In order of decreasing aa length the chimeric orfs from several of the chimeric mRNAs isolated were: A (418 aa) containing F3H Exons 1 and 2, the UP and CDC2 Exons; C (293 aa) with F3H Exon 1, 3'end 16 aa, and Exon 2 plus UP and CDC2 Exons; D (143 aa) had 9 aa of the UP intron plus the UP and CDC2 Exons. E (105 aa) with 34 aa of the FPK/MDH Intron plus 71 aa of the FPK Exon; F (105 aa) had 19 aa of FPK Intron, 77 aa of FPK Exon and 9 aa of MDH Exon. The non chimeric orf B (394 aa) had the three F3H Exons identical to the ones translated from the *Wp *allele the only cDNA clone with this orf was *wp*-15s. The chimeric orfs were generated from several of the cDNAs sequenced and they are listed underneath each orf class and also the frame in each one of the clones. * The orf from the *wp*-6c clone was 5 aa shorter. ** The orf from the *wp*-6c was 2 aa longer.

The amino acid sequences derived from the cDNA sequences of seed coat and cotyledon *wp*-cDNA clones shown in Figure 3B and 3C, varied significantly from clone to clone and consequently the putative open reading frames (orfs) of these chimeric transcripts. A search for orfs consisting of more than 100 amino acids (aa) found that many were chimeric (Figure 4 and additional file 3: Seed coat *wp *RT-PCR cDNA derived amino acid sequences and open reading frames, and additional file 4: Cotyledon *wp *RT-PCR cDNA derived amino acid sequences and open reading frames). Of interest were two putative chimeric orfs present in several of the *wp*-cDNAs. One was composed of approximately 210 bp (70 aa) of the *UP *exon fragment and 192 bp (64 aa) of the *CDC2 *exon fragment. It was present in seed coat transcripts *wp*-25s, -22s, -28s, -9s, -12s, -4s, and cotyledon transcripts *wp*-9c, -8c, -2c, -13c, -12c, -6c, always in a (+) frame. In half the clones this orf appears as just described (seed coat *wp*-25s, -22s, -12s and cotyledon *wp*-9c, -8c, -2c) (Figure 4-D). In the other half the orf is part of a larger chimeric orf containing also the *F3H1 *Exon 1 and Exon 2 sequences (Figure 4-A and 4C) (seed coat *wp*-28s, -9s, -4s and cotyledon *wp*-13c, -6c, -12c). A second chimeric orf predicted for five of the *wp*-cDNAs (seed coat *wp*-25s, -22s, -9s and cotyledon *wp*-9c, -8c) consisted of approximately 103 bp (35 aa) of *FPK/M *intronic fragment and 212 bp (70 aa) of *FPK *exonic region, always in one of the three (-) frames (Figure 4-E). A related chimeric orf containing *FPK *exonic sequence of approximately 231/199 bp (77/66 aa) appears in seed coat *wp*-12s clone and cotyledon *wp*-13c and *wp*-6c, all three in a (+) frame (Figure 4-F).

The products of these chimeric orfs may not serve enzymatic functions *per se *but if translated, they could potentially affect the function of wild type proteins synthesized from the intact host genes (*UP*, *CDC2 *or *FPK*). In addition to the chimeric orfs, we also found a cDNA that reconstituted the *F3H1*. The seed coat *wp*-15s cDNA clone in the (+3) frame contained an orf of 394 amino acids identical to the orf derived from the functional allele *F3H1 *of the purple flower isoline (*Wp*) (Figure 4-B). The product of this *F3H1*orf has the full potential to be translated into a functional F3H enzyme.

### Expression of host genes with homology to the *Tgm-Express1 *captured gene fragments

To analyze the expression of the host genes related to the exons captured by the *Tgm-Express1 *element, we amplified the cassette exons from the seed coat derived *wp*-12 cDNA clone (Figure 3B) to generate a chimeric radiolabeled probe that would hybridized to all RNAs with homology to the probe's exon fragments. These include those transcripts derived from the related host genes as well as the chimeric transcripts expressed from the *wp *mutant allele. This probe contained sequence fragments with similarity to an unknown protein (*UP*), cell division cycle 2 (*CDC2*), fructose-6-phosphate 2-kinase/fructose-2-6-biphosphatase (*FPK*), malate dehydrogenase (*M*) and cysteine synthase (*CS*) genes.

**Figure 5:**
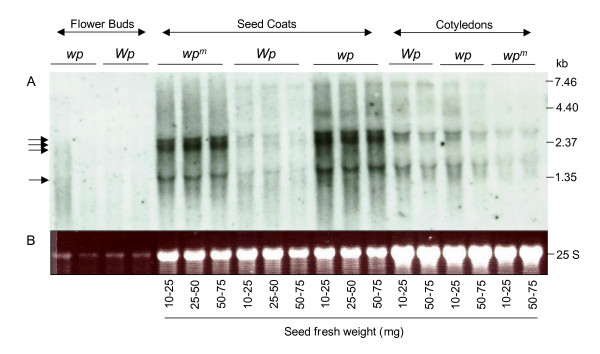
**Expression of the *Tgm-Express1 *captured gene fragments and related host genes in the *Wp*-flower color isolines. (A) **RNA gel blot with total RNA samples purified from flower buds, seed coats of three developmental stages and cotyledons of two developmental stages of the flower color isolines: LN89-5320-8-53 (*wp*^*m *^*wp*^*m*^), LN89-5320-6 (*WpWp*) and LN89-5322-2 (*wpwp*). Seed fresh weight of each seed coat and cotyledon sample in mg is shown at bottom. The chimeric DNA probe containing *UP, CDC2, FPK, M *and *CS *sequences was an amplification product from the seed coat RT-PCR derived *wp*-12 cDNA clone (Figure 3). **(B) **Ethidium bromide-stained gel prior to membrane transfer. The 25 S rRNA is shown to compare RNA sample loading.

Figure 5 shows the hybridization of the chimeric radiolabelled probe to an RNA blot containing flower bud, seed coat and cotyledon RNAs from the *wp*, *Wp *and *wp*^*m *^isolines. It appears that the hybridization signal in the seed coats from the *wp *line that contains the *Tgm-Express1 *insertion is greater than the signal in the *Wp *line that lacks the insertion, suggesting that the chimeric fragments within the *wp *allele are detected in the RNA blots along with hybridization to the host genes. In cotyledons on the other hand, hybridization levels to RNAs from both isolines *Wp *and *wp *were similar. Since we have previously shown that the *F3H1 *promoter is strongly expressed in seed coats but with practically undetectable expression in the cotyledons by RNA blot analysis [1], we deduced that the hybridization seen with cotyledon RNAs is likely to represent expression from one or more of the host genes. We have RNA blot data showing transcripts hybridizing to a soybean FPK cDNA clone (Gm-c1023-5325) of a size similar to those of the chimeric transcripts and that were more highly expressed in cotyledons of all three isolines than in seed coats (data not shown). This would explain the hybridization results observed for the cotyledons of the *Wp *where no chimeric transcripts should be synthesized.

In addition, the hybridizing RNAs shown in Figure 5 are large transcripts of size similar to those recovered by RT-PCR suggesting that most likely there are no internal transcription initiation sites within the *Tgm-Express1 *element to generate truncated smaller transcripts.

## Discussion

Plant transposable elements have long been known to cause changes in gene expression as a result of insertion or deletion in coding regions and gene promoters and their effect on RNA processing [14]. However, more than 20 years of research on tracking the movements of individual transposable elements at the molecular level has revealed only a few examples that these elements were capable of transposing non-element associated coding regions from the host genome. A part of a host gene was carried by a *Mutator *(*Mu*) related element [15] and another by the *Bs1 *retroviral element [16]. More recently, with the availability of entire genome sequences for rice and *Arabidopsis*, many *Mu*-like elements associated with fragments of host cellular genes have been found in those two plant species [17,18,4]. Bioinformatics analysis of the rice genome uncovered 898 intact retrogenes of which 380 were predicted to have chimeral protein coding sequences and several of these were confirmed by expression data [7]. A novel family of maize transposons, the *Helitrons*, has been found recently to be embedded with portions of cellular gene fragments [8,19,5,6]. The *Tgm-Express1*, a member of the CACTA family, was shown to carry four genic fragments [1]. An additional exonic region, UP, was identified upon analysis of the RT-PCR cDNA sequences obtained in the present study. More significantly, we show also that the *wp *allele carrying the *Tgm-Express1 *spawns an array of chimeric transcripts resulting from alternative splicing events at this locus, some of which lead to novel open reading frames.

If one were to envision an evolutionary advantageous mechanism for *the novo *generation of mutations, it likely would be one that would alter the splicing of a gene's constitutive exons to produce novel proteins without the complete loss of the wild-type protein. The *wp *pink flower mutation of soybean that we describe offers an example of how a transposon insertion in the intron of the wild-type gene (*F3H1*) could generate an array of alternatively spliced transcripts with potential to be translated into novel proteins without totally losing the ability to synthesize the wild-type transcript and encoded protein. The transposon insertion, *Tgm-Express1*, responsible for this mutation carries five host gene fragments, *UP, CDC2, FPK, M *and *CS*, which we show are exons that are alternatively spliced with *F3H1 *exons. Sequence analysis of multiple RT-PCR derived cDNAs from two different tissues, the seed coat and the cotyledon, revealed the processing of at least 12 distinct putative transcripts, 11 of which contained besides the F3H exons (1, 2 and 3) varying numbers of cassette exons with or without small host gene-fragment intronic portions. In addition, transcripts with only the constitutive F3H exons were also synthesized from the *wp *mutant allele. Although observations of element removal from maize exons [20,21] and introns [22] has previously been observed, the splicing of the very large *wp *intron of 6.44 kb (consisting of the 718 bp F3H intron 2 and the 5722 bp *Tgm-Express1 *transpon within it) is an unusual event. Plant introns are generally short and splicing of extremely large introns is rare [10].

In alternative splicing processes in other eukaryotic systems, splicing of the pre-messenger RNA required five ribonucleoproteins and numerous accessory proteins to form four complexes (E, A, B and C) to join two adjacent exons with the release of an intervening sequence [9]. It is remarkable to see the variety of processed transcripts that were created from the *wp *mutant allele leading to the precise splicing of the F3H and the cassette exons from within the *Tgm-Express1 *element (Figure 3B and 3C, seed coat *wp-*4s, *wp-*15s and cotyledon *wp*-12c and *wp*-6c, for example). Of greater interest also is the proper processing of the three CDC2 and two FPK exons in some of the transcripts that results in fusion of unrelated exons.

Analysis of the derived amino acid sequence from the 12 distinct *wp *chimeric cDNA sequences predicted putative chimeric open reading frames (orfs) varying in location and length within the cDNAs (Figure 4). Alternative splicing of the cassette exons within *Tgm-Express1 *creates premature termination codons in many of the transcripts which could lead to their being targets for nonsense-mediated mRNA decay, the surveillance mechanism that degrades selectively nonsense mRNAs [23]. However the larger 5'-end chimeric orfs (seed coats *wp*-28s, -9s, and cotyledons *wp*-13c and *wp-*6c) may have a higher chance to be translated (Figure 4-A). This orf is 418 aa, 24 aa longer than the one like the F3H wild type orf of 394 aa (Figure 4-B).

The correctly processed transcript, represented by the isolated *wp*-15s clone (Figure 4-B), would allow the synthesis of functional F3H protein which could explain the existence of residual anthocyanin pigment in the pink flowers and the lighter colored seed coats in plants with *wpwp *genotype (Figure 1). F3H function is a required step in the synthesis of all three branches of anthocyanin and proanthocyanidins (Figure 1D). The results presented here demonstrate that *wp *is not a null mutation and that correctly spliced F3H1 transcripts are synthesized in sufficient amounts to allow the synthesis of anthocyanin pigment coloring the pink flowers (Figure 1A) and seed coats of plants with the *i*, *t*, *wp *(Figure 1B) and *i*, *T*, *wp*, (Figure 1C) genotypes. The fact that *wp *is not a null mutation suggests that the residual anthocyanins being synthesized in the *wp *flowers are most likely the same as in the *Wp *purple flowered isolines. The only difference between the colors of the two phenotypes may be the amount of pigment being synthesized.

We cloned two similar F3H genomic sequences (*F3H1 *and *F3H2*) and found that the *F3H2 *gene is not expressed in the tissues discussed here [1]. Therefore, the residual flower and seed coat color- phenotypes displayed by *wp *allele (Figure 1 right panels) are not the result of the expression of other *F3H *family member genes elsewhere in the genome.

Except for the wild-type like orf in the *wp*-15 cDNA clone with the three correctly spliced Exons (1, 2 and 3), that reconstitute F3H, most other orfs of more than 100 amino acids were chimeric. The more extensive ones were composed of the two first exons of *F3H *and varying portions of *UP *and *CDC2 *(418 aa and 293 aa; Figure 4-A and 4C). In addition, two other chimeric orfs encoding 143 and 105 amino acids respectively were created in several of the cDNA clones analyzed. The first contains portions of *UP *and *CDC2 *sequences in (+) frames while the second has *FPK *and *FPK/M *intron-derived sequences in (-) frames (Figure 4-D and 4E). Since both the host genes and the *wp *chimeric transcripts appear to be weakly expressed in the tissues examined (Figure 5 and data not shown), any polypeptide fragments translated from the putative orfs, might interfere with the assembly and function of their active host protein counterparts. Thus, the CDC2 and FPK polypeptide pieces translated from the *wp *mutant allele may have potential to interfere with the functional enzymes translated from intact *CDC2 *or *FPK *host genes. Intriguingly, the second phenotype manifested in the *wp *mutant plants is seeds with lower oil and higher protein content than the *Wp *plants [2,3]. Inhibition of key metabolic enzymes such as FPK could potentially influence the direction of metabolic flux resulting in decreased fatty acid metabolism and linked increases in protein synthesis.

## Conclusion

The multiplicity of transcript isoforms described here add an additional layer of complexity reinforcing the tremendous potential these gene-fragment-loaded-transposon elements such as *Tgm-Express*, Pack-MULEs, *Helitrons*, and retroelements can have not only in disrupting or modifying gene function but in the creation of new or modified genes leading to an increase in plant genome and possibly proteome diversity. Analysis of human and other vertebrate genomes [12,13] revealed that recently evolved exons are more likely to be alternatively spliced cassette exons originating from highly repeated DNA elements including transposons, SINEs, LINEs, and Alu repeats, emphasizing the importance of mobile elements in creating diversity during evolution of both animal and plant species.

## Methods

### Plant material and genotypes

The *Glycine max *cultivars and isolines used for this study were: LN89-5320-6 (*Wp*, purple-flowered plants), LN89-5322-2 (*wp*, a stable line with pink flowered plants), and LN89-5320-8-53 (*wp*^*m*^, a mutable line with chimeric flower colors or sectors of pink and purple flowers). Each is homozygous for the indicated alleles of the *Wp *locus. The origin, genetics, and isolation of the *wp *allele have been described previously [2,3,25,1]. Plants were grown in the greenhouse. Seed coats dissected from seeds at varying stages of development, cotyledons of various stages of seed development, flower buds, stems, mature leaves and roots from two week old plants, and shoot tips (meristems surrounded by primordial leaves) were frozen in liquid nitrogen, freeze dried (Multi-dry lyophilazer; FTS systems), and stored at -20°C. For seed coat's (cotyledon's) developmental stages, seeds were divided into the following groups according to the fresh weight of the entire seed: 10–25 mg, 25–50 mg, 50–75 mg.

### RNA extraction, purification and cDNA synthesis

Total RNA was isolated from seed coats and other soybean tissues using a phenol-chloroform and lithium chloride precipitation method [25,26]. RNA was stored at -70°C until used.

cDNA copies of the *F3H1 *gene from the three isolines (LN89-5320-6, LN89-5322-2 and LN89-5320-8-53) were amplified from a first-strand cDNA pool synthesized using 1 μg of seed coat or cotyledon total RNA and the Superscript first strand synthesis system for reverse transcriptase (RT)-PCR with oligo (dT)_12–18 _primers (Invitrogen, San Diego). The total RNAs used for these RT-PCR reactions were treated with DNAaseI using Ambion's DNA-free kit and concentrated in Microcon YM-30 columns (Millipore, Bedford, MA). For each RNA sample, parallel reactions were allowed in the absence of Superscript (- controls) to assess the extent of DNA contamination. The sequences of the two primers used were: 5'-GCATTGCATTCTGCTATTTAATTCC-3' (7F) and 5'-AAAGACAGTGCCACTTATTTTCATT-3' (1428R). These primers map at the 5' and 3' ends of the *F3H1 *gene respectively. The numbering correspond to the base pair of the *F3H1 *gene cDNA sequence (Figure 3A)

### Primer synthesis, PCR reaction conditions, cDNA cloning and DNA sequencing

Oligonucleotide primers were synthesized on an Applied Biosystems (Foster City, CA) model 394A DNA synthesizer at the Keck Center, a unit of the University of Illinois Biotechnology Center. PCR reactions were performed by an initial denaturation step at 94°C for 2 min followed by 30 cycles of denaturing at 94°C for 30 sec, annealing at 56°C for 1 min, extension at 68°C for 9 min, to end with a 10 min extension at 72°C. High-fidelity and -efficiency *ExTaq *(Takara Bio Inc. Otsu, Japan) polymerase was used at 0.75 units per 50 μl reaction. Amplified cDNAs were separated from oligonucleotides with a QIAquick PCR Purification kit (QIAGEN), cloned into pGem-T easy and sequenced in an ABI 3730 × l (Applied Biosystems, Inc. Foster City, CA) at the Keck Center.

### RNA gel-blot analysis and cDNA probe synthesis

RNA (10 μg/sample) was electrophoresed in a 1.2% agarose-3% formaldehyde gel [27]. Size-fractionated RNAs were transferred to Optitran-supported nitrocellulose membrane (Midwest Scientific, Valley Park, MO) by capillary action as described in Sambrook *et al*. (1989) [27] and cross-linked with UV light (Stratagene, La Jolla, CA). Nitrocellulose RNA blots were prehybridized, hybridized, washed, and exposed to Hyperfilm (Amersham, Arlington Heights, IL) as described by Todd and Vodkin (1996) [28]. All the RNA blot results presented are from autoradiographs exposed for 7 days.

Cloned DNAs used as probes were PCR amplified, electrophoresed, and purified from the agarose using the QIAquick gel extraction kit (QIAGEN, Valencia, CA). DNA concentration of the final eluate was determined with a NanoDrop (NanoDrop Technologies, Inc. Rockland, DE). Purified DNA fragments (25–250 ng) were labeled with [a-^32^P]dATP by random primer reaction [29].

## Authors' contributions

GZ designed and performed the experiments, analyzed the results and drafted the manuscript.

LV led the research and edited the manuscript.

## Supplementary Material

Additional file 1 Seed coat *wp *RT-PCR cDNA sequence alignment. Click here for file

Additional file 2 Cotyledons *wp *RT-PCR cDNA sequence alignment. Click here for file

Additional file 3 See coat *wp *RT-PCR cDNA derived anubi acxud sequences and open reading frames. Click here for file

Additional file 4 Cotyledon *wp *RT-PCR cDNA derived amino acid sequences and open reading frames. Click here for file
