# Supplementary material for: Novel exon combinations generated by alternative splicing of gene fragments mobilized by a CACTA transposon in Glycine max
Source: BMC Plant Biol. 2007 Jul 14;7:38. doi: 10.1186/1471-2229-7-38 (PMC1947982; doi:10.1186/1471-2229-7-38)
Supplement: Additional file 1 — Seed coat wp RT-PCR cDNA sequence alignment. [file 1471-2229-7-38-S1.doc]

**Seed Coat *wp* RT-PCR cDNA clones**

1 60

wp-25s GCATTGCATT CTGCTATTTA ATTCCACTAC GTACACGCAC ATTCTCCTCA AAGACAACAA

wp-22s GCATTGCATT CTGCTATTTA ATTCCACTAC GTACACGCAC ATTCTCCTCA AAGACAACAA

wp-28s GCATTGCATT CTGCTATTTA ATTCCACTAC GTACACACAC ATTCTCCTCA AAGACAACAA

wp-9s GCATTGCATT CTGCTATTTA ATTCCACTAC GTACACGCAC ATTCTCCTCA AAGACAACAA

wp-12s GCATTGCATT CTGCTATTTA ATTCCACTAC GTACACGCAC ATTCTCCTCA AAGACAACAA

wp-4s GCATTGCATT CTGCTATTTA ATTCCACTAC GTACACGCAC ATTCTCCTCA AAGACAACAA

wp-15s GCATTGCATT CTGCTATTTA ATTCCACTAC GTACACGCAC ATTCTCCTCA AAGACAACAA

Consensus GCATTGCATT CTGCTATTTA ATTCCACTAC GTACACgCAC ATTCTCCTCA AAGACAACAA

61 120

wp-25s TGGCACCAAC AGCCAAGACT CTGACTTACC TGGCCCAGGA GAAAACCCTA GAATCGAGCT

wp-22s TGGCACCAAC AGCCAAGACT CTGACTTACC TGGCCCAGGA GAAAACCCTA GAATCGAGCT

wp-28s TGGCACCAAC AGCCAAGACT CTGACTTACC TGGCCCAGGA GAAAACCCTA GAATCGAGCT

wp-9s TGGCACCAAC AGCCAAGACT CTGACTTACC TGGCCCAGGA GAAAACCCTA GAATCGAGCT

wp-12s TGGCACCAAC AGCCAAGACT CTGACTTACC TGGCCCAGGA GAAAACCCTA GAATCGAGCT

wp-4s TGGCACCAAC AGCCAAGACT CTGACTTACC TGGCCCAGGA GAAAACCCTA GAATCGAGCT

wp-15s TGGCACCAAC AGCCAAGACT CTGACTTACC TGGCCCAGGA GAAAACCCTA GAATCGAGCT

Consensus TGGCACCAAC AGCCAAGACT CTGACTTACC TGGCCCAGGA GAAAACCCTA GAATCGAGCT

121 180

wp-25s TCGTTCGGGA CGAGGAGGAG CGTCCCAAGG TTGCCTACAA CGAATTCAGC GACGAGATCC

wp-22s TCGTTCGGGA CGAGGAGGAG CGTCCCAAGG TTGCCTACAA CGAATTCAGC GACGAGATCC

wp-28s TCGTTCGGGA CGAGGAGGAG AGTCCCAAGG TTGCCTACAA CGAATTCAGC GACGAGATCC

wp-9s TCGTTCGGGA CGAGGAGGAG CGTCCCAAGG TTGCCTACAA CGAATTCAGC GACGAGATCC

wp-12s TCGTTCGGGA CGAGGAGGAG CGTCCCAAGG TTGCCTACAA CGAATTCAGC GACGAGATCC

wp-4s TCGTTCGGGA CGAGGAGGAG CGTCCCAAGG TTGCCTACAA CGAATTCAGC GACGAGATCC

wp-15s TCGTTCGGGA CGAGGAGGAG CGTCCCAAGG TTGCCTACAA CGAATTCAGC GACGAGATCC

Consensus TCGTTCGGGA CGAGGAGGAG cGTCCCAAGG TTGCCTACAA CGAATTCAGC GACGAGATCC

181 240

wp-25s CAGTGATTTC TCTTGCCGGA ATCGACGAGG TGGATGGACG CAGAAGAGAG ATTTGTGAGA

wp-22s CAGTGATTTC TCTTGCCGGA ATCGACGAGG TGGATGGACG CAGAAGAGAG ATTTGTGAGA

wp-28s CAGTGATTTC TCTTGCCGGA ATCGACGAGG TGGATGGACG CAGAAGAGAG ATTTGTGAGA

wp-9s CAGTGATTTC TCTTGCCGGA ATCGACGAGG TGGATGGACG CAGAAGAGAG ATTTGTGAGA

wp-12s CAGTGATTTC TCTTGCCGGA ATCGACGAGG TGGATGGACG CAGAAGAGAG ATTTGTGAGA

wp-4s CAGTGATTTC TCTTGCCGGA ATCGACGAGG TGGATGGACG CAGAAGAGAG ATTTGTGAGA

wp-15s CAGTGATTTC TCTTGCCGGA ATCGACGAGG TGGATGGACG CAGAAGAGAG ATTTGTGAGA

Consensus CAGTGATTTC TCTTGCCGGA ATCGACGAGG TGGATGGACG CAGAAGAGAG ATTTGTGAGA

241 300

wp-25s AGATCGTGGA GGCTTGCGAG AATTGGGGTA TATTCCAGGT TGTTGATCAC GGTGTGGATC

wp-22s AGATCGTGGA GGCTTGCGAG AATTGGGGTA TATTCCAGGT TGTTGATCAC GGTGTGGATC

wp-28s AGATCGTGGA GGCTTGCGAG AATTGGGGTA TATTCCAGGT TGTTGATCAC GGTGTGGATC

wp-9s AGATCGTGGA GGCTTGCGAG AATTGGGGTA TATTCCAGGT TGTTGATCAC GGTGTGGATC

wp-12s AGATCGTGGA GGCTTGCGAG AATTGGGGTA TATTCCAGGT TGTTGATCAC GGTGTGGATC

wp-4s AGATCGTGGA GGCTTGCGAG AATTGGGGTA TATTCCAGGT TGTTGATCAC GGTGTGGATC

wp-15s AGATCGTGGA GGCTTGCGAG AATTGGGGTA TATTCCAGGT TGTTGATCAC GGTGTGGATC

Consensus AGATCGTGGA GGCTTGCGAG AATTGGGGTA TATTCCAGGT TGTTGATCAC GGTGTGGATC

301 360

wp-25s AACAACTCGT GGCCGAGATG ACCCGTCTCG CCAAAGAGTT CTTTGCTTTG CCACCGGACG

wp-22s AACAACTCGT GGCCGAGATG ACCCGTCTCG CCAAAGAGTT CTTTGCTTTG CCACCGGACG

wp-28s AACAACTCGT GGCCGAGATG ACCCGTCTCG CCAAAGAGTT CTTTGCTTTG CCACCGGACG

wp-9s AACAACTCGT GGCCGAGATG ACCCGTCTCG CCAAAGAGTT CTTTGCTTTG CCACCGGACG

wp-12s AACAACTCGT GGCCGAGATG ACCCGTCTCG CCAAAGAGTT CTTTGCTTTG CCACCGGACG

wp-4s AACAACTCGT GGCCGAGATG ACCCGTCTCG CCAAAGAGTT CTTTGCTTTG CCACCGGACG

wp-15s AACAACTCGT GGCCGAGATA ACCCGTCTCG CCAAAGAGTT CTTTGCTTTG CCACCGGACG

Consensus AACAACTCGT GGCCGAGATg ACCCGTCTCG CCAAAGAGTT CTTTGCTTTG CCACCGGACG

361 420

wp-25s AGAAGCTTCG TTTTGATATG TCCGGCGCCA AAAAGGGTGG ATTCATTGTC TCCAGCCATC

wp-22s AGAAGCTTCG TTTTGATATG TCCGGCGCCA AAAAGGGTGG ATTCATTGTC TCCAGCCATC

wp-28s AGAAGCTTCG TTTTGATATG TCCGGCGCCA AAAAGGGTGG ATTCATTGTC TCCAGCCATC

wp-9s AGAAGCTTCG TTTTGATATG TCCGGCGCCA AAAAGGGTGG ATTCATTGTC TCCAGCCATC

wp-12s AGAAGCTTCG TTTTGATATG TCCGGCGCCA AAAAGGGTGG ATTCATTGTC TCCAGCCATC

wp-4s AGAAGCTTCG TTTTGATATG TCCGGCGCCA AAA-GGGTGG ATTCATTGTC TCCAGCCATC

wp-15s AGAAGCTTCG TTTTGATATG TCCGGCGCCA AAAAGGGTGG ATTCATTGTC TCCAGCCATC

Consensus AGAAGCTTCG TTTTGATATG TCCGGCGCCA AAAaGGGTGG ATTCATTGTC TCCAGCCATC

421 480

wp-25s TCCAAGGGGA ATCGGTGCAG GACTGGAGAG AAATAGTGAC ATACTTTTCG TACCCAAAAA

wp-22s TCCAAGGGGA ATCGGTGCAG GACTGGAGAG AAATAGTGAC ATACTTTTCG TACCCAAAAA

wp-28s TCCAAGGGGA ATCGGTGCAG GACTGGAGAG AAATAGTGAC ATACTTTTCG TACCCAAAAA

wp-9s TCCAAGGGGA ATCGGTGCAG GACTGGAGAG AAATAGTGAC ATACTTTTCG TACCCAAAAA

wp-12s TCCAAG---- ---------- -ACTGGAGAG AAATAGTGAC ATACTTTTCG TACCCAAAAA

wp-4s TCCAAGGGGA ATCGGTGCAG GACTGGAGAG AAATAGTGAC ATACTTTTCG TACCCAAAAA

wp-15s TCCAAGGGGA ATCGGTGCAG GACTGGAGAG AAATAGTGAC ATACTTTTCG TACCCAAAAA

Consensus TCCAAGggga atcggtgcag gACTGGAGAG AAATAGTGAC ATACTTTTCG TACCCAAAAA

481 540

wp-25s GAGAGAGGGA CTATTCAAGG TGGCCAGACA CGCCAGAAGG GTGGAGATCG GTGACTGAGG

wp-22s GAGAGAGGGA CTATTCAAGG TGGCCAGACA CGCCAGAAGG GTGGAGATCG GTGACTGAGG

wp-28s GAGAGAGGGA CTATTCAAGG TGGCCAGACA CGCCAGAAGG GTGGAGATCG GTGACTGAGG

wp-9s GAGAGAGGGA CTATTCAAGG TGGCCAGACA CGCCAGAAGG GTGGAGATCG GTGACTGAGG

wp-12s GAGAGAGGGA CTATTCAAGG TGGCCAGACA CGCCAGAAGG GTGGAGATCG GTGACTGAGG

wp-4s GAGAGAGGGA CTATTCAAGG TGGCCAGACA CGCCAGAAGG GTGGAGATCG GTGACTGAGG

wp-15s GAGAGAGGGA CTATTCAAGG TGGCCAGACA CGCCAGAAGG GTGGAGATCG GTGACTGAGG

Consensus GAGAGAGGGA CTATTCAAGG TGGCCAGACA CGCCAGAAGG GTGGAGATCG GTGACTGAGG

541 600

wp-25s AATACAGCGA CAAAGTAATG GGTCTAGCTT GCAAGCTCAT GGAGGTGTTG TCCGAAGCAA

wp-22s AATACAGCGA CAAAGTAATG GGTCCATCTT GCAAGCTCAT GGAGGTGTTG TCCGAAGCAA

wp-28s AATACAGCGA CAAAGTAATG GGTCTAGCTT GCAAGCTCAT GGAGGTGTTG TCCGAAGCAA

wp-9s AATACAGCGA CAAAGTAATG GGTCTAGCTT GCAAGCTCAT GGAGGTGTTG TCCGAAGCAA

wp-12s AATACAGCGA CAAAGTAATG GGTCTAGCTT GCAAGCTCAT GGAGGTGTTG TCCGAAGCAA

wp-4s AATACAGCGA CAAAGTAATG GGTCTAGCTT GCAAGCTCAT GGAGGTGTTG TCCGAAGCAA

wp-15s AATACAGCGA CAAAGTAATG GGTCTAGCTT GCAAGCTCAT GGAGGTGTTG TCCGAAGCAA

Consensus AATACAGCGA CAAAGTAATG GGTCtAgCTT GCAAGCTCAT GGAGGTGTTG TCCGAAGCAA

601 660

wp-25s TGGGGTTAGA GAAAGAGGGT TTAAGCAAAG CATGTGTTGA CATGGACCAG AAGGTGGTGG

wp-22s TGGGGTTAGA GAAAGAGGGT TTAAGCAAAG CATGTGTTGA CATGGACCAG AAGGTGGTGG

wp-28s TGGGGTTAGA GAAAGAGGGT TTAAGCAAAG CATGTGTTGA CATGGACCAG AAGGTGGTGG

wp-9s TGGGGTTAGA GAAAGAGGGT TTAAGCAAAG CATGTGTTGA CATGGACCAG AAGGTGGTGG

wp-12s TGGGGTTAGA GAAAGAGGGT TTAAGCAAAG CATGTGTTGA CATGGACCAG AAGGTGGTGG

wp-4s TGGGGTTAGA GAAAGAGGGT TTAAGCAAAG CATGTGTTGA CATGGACCAG AAGGTGGTGG

wp-15s TGGGGTTAGA GAAAGAGGGT TTAAGCAAAG CATGTGTTGA CATGGACCAG AAGGTGGTGG

Consensus TGGGGTTAGA GAAAGAGGGT TTAAGCAAAG CATGTGTTGA CATGGACCAG AAGGTGGTGG

661 720

wp-25s TTAATTACTA CCCCAAATGC CCTCAACCTG ACCTCACTCT TGGCCTGAAG CGCCACACGG

wp-22s TTAATTACTA CCCCAAATGC CCTCAACCTG ACCTCACTCT TGGCCTGAAG CGCCACACGG

wp-28s TTAATTACTA CCCCAAATGC CCTCAACCTG ACCTCACTCT TGGCCTGAAG CGCCGCACGG

wp-9s TTAATTACTA CCCCAAATGC CCTCAACCTG ACCTCACTCT TGGCCTGAAG CGCCACACGG

wp-12s TTAATTACTA CCCCAAATGC CCTCAACCTG ACCTCACTCT TGGCCTGAAG CGCCACACGG

wp-4s TTAATTACTA CCCCAAATGC CCTCAACCTG ACCTCACTCT TGGCCTGAAG CGCCACACGG

wp-15s TTAATTACTA CCCCAAATGC CCTCAACCTG ACCTCACTCT TGGCCTGAAG CGCCACACGG

Consensus TTAATTACTA CCCCAAATGC CCTCAACCTG ACCTCACTCT TGGCCTGAAG CGCCaCACGG

721 780

wp-25s ATCCGGGCAC TATCACCTTG CTGCTTCAGG ACCAAGTGGG TGGACTTCAA GCCACCAGGG

wp-22s ATCCGGGCAC TATCACCTTG CTGCTTCAGG ACCAAGTGGG TGGACTTCAA GCCACCAGGG

wp-28s ATCCGGGCAC TATCACCTTG CTGCTTCAGG ACCAAGTGGG TGGACTTCAA GCCACCAGGG

wp-9s ATCCGGGCAC TATCACCTTG CTGCTTCAGG ACCAAGTGGG TGGACTTCAA GCCACCAGGG

wp-12s ATCCGGGCAC TATCACCTTG CTGCTTCAGG ACCAAGTGGG TGGACTTCAA GCCACCAGGG

wp-4s ATCCGGGCAC TATCACCTTG CTGCTTCAGG ACCAAGTGGG TGGACTTCAA GCCACCAGGG

wp-15s ATCCGGGCAC TATCACCTTG CTGCTTCAGG ACCAAGTGGG TGGACTTCAA GCCACCAGGG

Consensus ATCCGGGCAC TATCACCTTG CTGCTTCAGG ACCAAGTGGG TGGACTTCAA GCCACCAGGG

781 840

wp-25s ACAATGGCAA AACATGGATC ACCGTTCAGC CTGTGGAGGC TGCCTTCGTC GTCAATCTTG

wp-22s ACAATGGCAA AACATGGATC ACCGTTCAGC CTGTGGAGGC TGCCTTCGTC GTCAATCTTG

wp-28s ACAATGGCAA AACATGGATC ACCGTTCAGC CTGTGGAGGC TGCCTTCGTC GTCAATCTTG

wp-9s ACAATGGCAA AACATGGATC ACCGTTCAGC CTGTGGAGGC TGCCTTCGTC GTCAATCTTG

wp-12s ACAATGGCAA AACATGGATC ACCGTTCAGC CTGTGGAGGC TGCCTTCGTC GTCAATCTTG

wp-4s ACAATGGCAA AACATGGATC ACCGTTCAGC CTGTGGAGGC TGCCTTCGTC GTCAATCTTG

wp-15s ACAATGGCAA AACATGGATC ACCGCTCAGC CTGTGGAGGC TGCCTTCGTC GTCAATCTTG

Consensus ACAATGGCAA AACATGGATC ACCGtTCAGC CTGTGGAGGC TGCCTTCGTC GTCAATCTTG

841 900

wp-25s GAGATCATGC TCATGTAGAC CACGATGGCA TTTTTGTAAT TCGAAGACTT TATTCTAAGA

wp-22s GAGATCATGC TCAT----AC CACGATGGCA TTTTTGTAAT TCGAAGACTT TATTCTAAGA

wp-28s GAGATCATGC TCAT------ ---------- ---------- ---------- ----------

wp-9s GAGATCATGC TCAT------ ---------- ---------- ---------- ----------

wp-12s GAGATCATGC TCATGTAGAC CACGATGGCA TTTTTGTAAT TCGAAGACTT TATTCTAAGA

wp-4s GAGATCATGC TCAT------ ---------- ---------- ---------- ----------

wp-15s GAGATCATGC TCAT------ ---------- ---------- ---------- ----------

Consensus GAGATCATGC TCAT...... .......... .......... .......... ..........

901 960

wp-25s CGAGTGTCAT TAGAAAACGT CGTTGTTAGT TTAGAAAAAA AAA-GTTATA CGCGTTCTGA

wp-22s CGAGTGTCAT TAGAAAACGT CGTTGTTAGT TTAGAAAAAA AAAAGTTATA CGCGTTCTGA

wp-28s ---------- ---------- ---------- ---------- ---------- ----------

wp-9s ---------- ---------- ---------- ---------- ---------- ----------

wp-12s CGAGTGTCAT TAGAAAACGT CGTTGTTAGT TTAGAAAAAA AAAAGTTATA CGCGTTCTGA

wp-4s ---------- ---------- ---------- ---------- ---------- ----------

wp-15s ---------- ---------- ---------- ---------- ---------- ----------

Consensus .......... .......... .......... .......... .......... ..........

961 1020

wp-25s ACCCTAAGCA AGGTCACAAC CTCGAAGCTG AGTTCTTTCT ACTGTGCTGC ATCAACAGTT

wp-22s ACCCTAAGCA AGGTCACAAC CTCGAAGCTG AGTTCTTTCT ACTGTGCTGC ATCAACAGTT

wp-28s ---------- ---------- ---------- ---------- ---------- --------TT

wp-9s ---------- ---------- ---------- ---------- ---------- --------TT

wp-12s ACCCTAAGCA AGGTCACAAC CTCGAAGCTG AGTTCTTTCT ACTGTGCTGC ATCAACAGTT

wp-4s ---------- ---------- ---------- ---------- ---------- --------TT

wp-15s ---------- ---------- ---------- ---------- ---------- ----------

Consensus .......... .......... .......... .......... .......... ........tt

1021 1080

wp-25s ATACGACGAC AATGACTGTG ACCTAAAATC ACCTCTGAAG AAACCTGATT CCGCATCCAC

wp-22s ATACGACGAC AATGACTGTG ACCTAAAATC ACCTCTGAAG AAACCCGATT CCGCATCCAC

wp-28s ATACGACGAC AATGACTGTG ACCTAAAATC ACCTCTGAAG AAACCCGATT CCGCATCCAC

wp-9s ATACGACGAC AATGACTGTG ACCTAAAATC ACCTCTGAAG AAACCCGATT CCGCATCCAC

wp-12s ATACGACGAC AATGACTGTG ACCTAAAATC ACCTCTGAAG AAACCCGATT CCGCATCCAC

wp-4s ATACGACGAC AATGACTGTG ACCTAAAATC ACCTCTGAAG AAACCCGATT CCGCATCCAC

wp-15s ---------- ---------- ---------- ---------- ---------- ----------

Consensus atacgacgac aatgactgtg acctaaaatc acctctgaag aaacccgatt ccgcatccac

1081 1140

wp-25s CCCCAGTCCC ACCGCGTGCG GCGTCAAAGA GGATTTCTCC GTGCTCACCA ACGACGAAGA

wp-22s CCCCAGTCCC ACCGCGTGCG GCGTCAAAGA GGATTTCTCC GTGCTCACCA ACGACGAAGA

wp-28s CCCCAGTCCC ACCGCGTGCG GCGTCAAAGA GGATTTCTCC GTGCTCACCA ACGACGAAGA

wp-9s CCCCAGTCCC ACCGCGTGCG GCGTCGAAGA GGTTTTCTCC GTGCTCACCA ACGACGAAGA

wp-12s CCCCAGTCCC ACCGCGTGCG GCGTCAAAGA GGATTTCTCC GTGCTCACCA ACGACGAAGA

wp-4s CCCCAGTCCC ACCGCGTGCG GCGTCAAAGA GGATTTCTCC GTGCTCACCA ACGACGAAGA

wp-15s ---------- ---------- ---------- ---------- ---------- ----------

Consensus ccccagtccc accgcgtgcg gcgtcaaaga ggatttctcc gtgctcacca acgacgaaga

1141 1200

wp-25s AGAAGAAGAT GTAATTGCCG GTATCCGAAA TGATTTCACA GAGTTAGTAG CAAGTTCAAG

wp-22s AGAAGAAGAT GTAATTGCCG GTATCCGAAA TGATTTCACA GAGTTAGTAG CAAGTTCAAG

wp-28s AGAAGAAGAT GTAATTGCCG GTATCCGAAA TGATTTCACA GAGTTAGTAG CAAGTTCAAG

wp-9s AGAAGAAGAT GTAATTGCCG GTATCCGAAA TGATTTCACA GAGTTAGTAG CAAGTTCAAG

wp-12s AGAAGAAGAT GTAATTGCCG GTATCCGAAA TGATTTCACA GAGTTAGTAG CAAGTTCAAG

wp-4s AGAAGAAGAT GTAATTGCCG GTATCCGAAA TGATTTCACA GAGTTAGTAG CAAGTTCAAG

wp-15s ---------- ---------- ---------- ---------- ---------- ----------

Consensus agaagaagat gtaattgccg gtatccgaaa tgatttcaca gagttagtag caagttcaag

1201 1260

wp-25s AACGGGATCT TCAAAATCTG CGAGGCTCAC TGTAACTCCT AACTTCCCAG ATTGTCATCT

wp-22s AACGGGATCT TCAAAATCTG CGAGGCTCAC TGTAACTCCT AACTTCCCAG ATTGTCATCT

wp-28s AACGGGATCT TCAAAATCTG CGAGGCTCAC TGTAACTCCT AACTTCCCAG ATTGTCATCT

wp-9s AACGGGATCT TCAAAATCTG CGAGGCTCAC TGTAACTCCT AACTTCCCAG ATTGTCATCT

wp-12s AACGGGATCT TCAAAATCTG CGAGGCTCAC TGTAACTCCT AACTTCCCAG ATTGTCATCT

wp-4s AACGGGATCT TCAAAATCTG CGAGGCTCAC TGTAACTCCT AACTTCCCAG ATTGTCATCT

wp-15s ---------- ---------- ---------- ---------- ---------- ----------

Consensus aacgggatct tcaaaatctg cgaggctcac tgtaactcct aacttcccag attgtcatct

1261 1320

wp-25s TCCTCTTCAG TCATCTACTT TTCCTCTTTT CTCTCCGACG ATTGTTGGAA CTCAAAGAGC

wp-22s TCCTCTTCAG TCATCTACTT TTCCTCTTTT CTCTCCGATG ATTGTTGGAA CTCAAAGAGC

wp-28s TCCTCTTCAG TCACCTACTT TTCCTCTTTT CTCTCCGATG ATTGTTGGAA CTCAAAGAGC

wp-9s TCCTCTTCAG TCATCTACTT TTCCTCTTTT CTCTCCGATG ATTGTTGGAA CTCAAAGAGC

wp-12s TCCTCTTCAG TCATCTACTT TTCCTCTTTT CTCTCCGATG ATTGTTGGAA CTCAAAGAGC

wp-4s TCCTCTTCAG TCATCTACTT TTCCTCTTTT CTCTCCGATG ATTGTTGGAA CTCAAAGAGC

wp-15s ---------- ---------- ---------- ---------- ---------- ----------

Consensus tcctcttcag tcatctactt ttcctctttt ctctccgatg attgttggaa ctcaaagagc

1321 1380

wp-25s TTCTTTGATG GAACAGTACG AAAAGGTTGA GAAGATAGGG TCGCGACCGC GTCACCAACG

wp-22s TTCTTTGATG GAACAGTACG AAAAGGTTGA GAAGATAGGG TCGCGACCGC GTCACCAACG

wp-28s TTCTTTGATG GAACAGTACG AAAAGGTTGA GAAGATAGGG TCGCGACCGC GTCACCAACG

wp-9s TTCTTTGATG GAACAGTACG AAAAGGTTGA GAAGATAGGG TCGCGACCGC GTCACCAACG

wp-12s TTCTTTGATG GAACAGTACG AAGAGGTTGA GAAGATAGGG TCGCGACCGC GTCACCAACG

wp-4s TTCTTTGATG GAACAGTACG AAAAGGTTGA GAAGATAGGG TCGCGACCGC GTCACCAACG

wp-15s ---------- ---------- ---------- ---------- ---------- ----------

Consensus ttctttgatg gaacagtacg aaaaggttga gaagataggg tcgcgaccgc gtcaccaacg

1381 1440

wp-25s AGACCATCGC GTTGAAGAAG ATTCGCCTCG AGCAGGAGGA TGAGGGGGTT CCCAGCACCG

wp-22s AGACCATCGC GTTGAAGAAG ATTCGCCTCG AGCAGGAGGA TGAGGGGGTT CCCAGCACCG

wp-28s AGACCATCGC GTTGAAGAAG ATTCGCCTCG AGCAGGAGGA TGAGGGGGTT CCCAGCACCG

wp-9s AGACCATCGC GTTGAAGAAG ATTCGCCTCG AGCAGGAGGA TGAGGGGGTT CCCAGCACCG

wp-12s AGACCATCGC GTTGAAGAAG ATTCGCCTCG AGCAGGAGGA TGAGGGGGTT CCCAGCACCG

wp-4s AGACCATCGC GTTGAAGAAG ATTCGCCTCG AGCAGGAGGA TGAGGGGGTT CCCAGCACCG

wp-15s ---------- ---------- ---------- ---------- ---------- ----------

Consensus agaccatcgc gttgaagaag attcgcctcg agcaggagga tgagggggtt cccagcaccg

1441 1500

wp-25s CCATTCACGA GATTTCTCTC TTGAAAGAAA TGCAGCACAG GAACATTGTT AGGTTGTAGG

wp-22s CCATTCACGA GATTTCACTC TTGAAAGAAA TGCAGCACAG GAACATTGTT AGGTTGTAGG

wp-28s CCATTCACGA GATTTCTCTC TTGAAAGAAA TGCAGCACAG GAACATTGTT AGGTTGTAGG

wp-9s CCATTCACGA GATTTCTCTC TTGAAAGAAA TGCAGCACAG GAACATTGTT AGGTTGTAGG

wp-12s CCATTCACGA GATTTCTCTC TTGAAAGAAA TGCAGCACAG GAACATTGTT AGGTTGTAGG

wp-4s CCATTCACGA GATTTCTCTC TTGAAAGAAA TGCAGCACAG GAACATTGTT AGGTTGTAGG

wp-15s ---------- ---------- ---------- ---------- ---------- ----------

Consensus ccattcacga gatttctctc ttgaaagaaa tgcagcacag gaacattgtt aggttgtagg

1501 1560

wp-25s ATGTAGTGCA CGATGAGAAG AGTTTGTATC TGGTACAATT GGTTTCTGAG GGAAGACAAA

wp-22s ATGTAGTGCA CGATGAGAAG AGTTTGTATC TGGTACAATT GGTTTCTGAG GGAAGACAAA

wp-28s ATGTAGTGCA CGATGAGAAG AGTTTGTATC TGGTACAATT GGTTTCTGAG GGAAGACAAA

wp-9s ATGTAGTGCA CGATGAGAAG AGTTTGTATC TGGTACAATT GGTTTCTGAG GGAAGACAAA

wp-12s ATGTAGTGCA CGATGAGAAG AGTTTGTATC TGGTACAATT GGTTTCTGAG GGAAGACAAA

wp-4s ATGTAGTGCA CGATGAGAAG AGTTTGTATC TGGTACAATT GGTTTCTGAG GGAAGACAAA

wp-15s ---------- ---------- ---------- ---------- ---------- ----------

Consensus atgtagtgca cgatgagaag agtttgtatc tggtacaatt ggtttctgag ggaagacaaa

1561 1620

wp-25s GGTAGTTAGA TAATAAATTC AAGGACTTTG TTGCAAGGTT GAAATGGTGT AATAGTTCTA

wp-22s GGTAGTTAGA TAATAAATTC AAGGACTTTG TTGCAAGGTT GAAATGGT-- ----------

wp-28s GGTAGTTAGA TAATAAATTC AAGGACTTTG TTGCAAGGTT GAAATGGT-- ----------

wp-9s GGTAGTTAGA TAATAAATTC AAGGACTTTG TTGCAAGGTT GAAATGGT-- ----------

wp-12s GGTAGTTAGA TAATAAATTC AAGGACTTTG TTGCAAGGTT GAAATGGTGT AATAGTTCTA

wp-4s GGTAGTTAGA TAATAAATTC AAGGACTTTG TTGCAAGGTT GAAATGGT-- ----------

wp-15s ---------- ---------- ---------- ---------- ---------- ----------

Consensus ggtagttaga taataaattc aaggactttg ttgcaaggtt gaaatggt.. ..........

1621 1680

wp-25s ACTTCTCTAT CCTTGAATTC TTGCTTCATT ACTCTCTTTT CTAATGTGAT --GTGGCATT

wp-22s ---------- ---------- ---------- ---------- ---------- ----------

wp-28s ---------- ---------- ---------- ---------- ---------- ----------

wp-9s ---------- ---------- ---------- ---------- ---------- ----------

wp-12s ACTTCTCTAT CCTTGAATTC TTGCTTCATT ACTCTCTTTT CTAATGTGAT ATGTGGCATT

wp-4s ---------- ---------- ---------- ---------- ---------- ----------

wp-15s ---------- ---------- ---------- ---------- ---------- ----------

Consensus .......... .......... .......... .......... .......... ..........

1681 1740

wp-25s AGAATCATTT TCAATATTTG GCTGAATACC TAGAACACTA TGCAATTTGT TATAACAGTG

wp-22s ---------- ---------- ---------- ---------- ---------- --------TG

wp-28s ---------- ---------- ---------- ---------- ---------- --------TG

wp-9s ---------- ---------- ---------- ---------- ---------- --------TG

wp-12s AGAATCATTT TCAATATTTG GCTGAATACC TAGAACACTA TGCAATTTGT TATAACAGTG

wp-4s ---------- ---------- ---------- ---------- ---------- --------TG

wp-15s ---------- ---------- ---------- ---------- ---------- ----------

Consensus .......... .......... .......... .......... .......... ........tg

1741 1800

wp-25s AGGCTGGAGA ACTTTATTCA AAGAAGCTTG CCAAGTTTGT TGGAAAGCGT CTCAAATCAG

wp-22s AGGCTGGAGA ACTTTATTCA AAGAAGCTTG CCAAGTTTGT TGGAAAGCGT CTCAAATCAG

wp-28s AGGCTGGAGA ACTTTATTCA AAGAAGCTTG CCAAGTTTGT TGGAAAGCGT CTCAAATCAG

wp-9s AGGCTGGAGA ACTTTATTCA AAGAAGCTTG CCAAGTTTGT TGGAAAGCGT CTCAAATCAG

wp-12s AGGCTGGAGA ACTTTATTCA AAGAAGCTTG CCAAGTTTGT TGGAAAGCGT CTCAAATCAG

wp-4s AGGCTGGAGA ACTTTATTCA AAGAAGCTTG CCAAGTTTGT TGGAAAGCGT CTCAAATCAG

wp-15s ---------- ---------- ---------- ---------- ---------- ----------

Consensus aggctggaga actttattca aagaagcttg ccaagtttgt tggaaagcgt ctcaaatcag

1801 1860

wp-25s AATGGGCTGC TTCT------ ---------- ATATGGACTA GTACACTGCA ACGAACAATT

wp-22s AATGGGCTGC TTCT------ ---------- ATATGGACTA GTACACTGCA ACGAACAATT

wp-28s AATGGGCTGC TTCTGTAAGC ATTATCTATT ATATGGACTA GTACACTGCA ACGAACAATT

wp-9s AATGGGCTGC TTCT------ ---------- ATATGGACTA GTACACTGCA ACGAACAATT

wp-12s AATGGGCTGC TTCT------ ---------- ATATGGACTA GTACACTGCA ACGAACAATT

wp-4s AATGGGCTGC TTCT------ ---------- ATATGGACTA GTACACTGCA ACGAACAATT

wp-15s ---------- ---------- ---------- ---------- ---------- ----------

Consensus aatgggctgc ttct...... .......... atatggacta gtacactgca acgaacaatt

1861 1920

wp-25s CTGACAGCCA CTCCAATTAT TGGATTTCCC AAGATACAAT GGCGTGCACT TGATGAGATA

wp-22s CTGACAGCCA CTCCAATTAT TGGATTTCCC AAGATACAAT GGCGTGCACT TGATGAGATA

wp-28s CTGACAGCCA CTCCAATTAT TGGATTTCCC AAGATACAAT GGCGTGCACT TGATGAGATA

wp-9s CTGACAGCCA CTCCAATTAT TGGATTTCCC AAGATACAAT GGCGTGCACT TGATGAGATA

wp-12s CTGACAGCCA CTCCAATTAT TGGATTTCCC AAGATACAAT GGCGTGCACT TGATGAGATA

wp-4s CTGACAGCCA CTCCAATTAT TGGATTTCCC AAGATACAAT GGCGTGCACT TGATGAGATA

wp-15s ---------- ---------- ---------- ---------- ---------- ----------

Consensus ctgacagcca ctccaattat tggatttccc aagatacaat ggcgtgcact tgatgagata

1921 1980

wp-25s AACGCAGGGG TGTGTGATGG TATGGCATAT GCAGAAATCA A---AAACAT GCCAGAGGAG

wp-22s AACGCAGGGG TGTGTGATGG TATGGCATAT GCAGAAATCA AGAAAAACAT GCCAGAGGAG

wp-28s AACGCAGGGG TGTGTGATGG TATGGCATAT GCAGAAATCA AGAAAAACAT GCCAGAGGAG

wp-9s AACGCAGGGG TGTGTGATAG TATGGCATAT GCAGAAATCA AGAAAAACAT GCCAGAGGAG

wp-12s AACGCAGGGG TGTGTGATGG TATGGCATAT GCAGAAATCA AGAAAAGCAT GCCAGAGGAG

wp-4s AACGCAGGGG TGTGTGATGG TATGGCATAT GCAGAAATCA AGAAAAACAT GCCAGAGGAG

wp-15s ---------- ---------- ---------- ---------- ---------- ----------

Consensus aacgcagggg tgtgtgatgg tatggcatat gcagaaatca agaaaaacat gccagaggag

1981 2040

wp-25s TATGAGTAGG TTTGCAGACT TGTAATTTTT CCCTTATTTG CAAGTTGCAT GCAACTAATA

wp-22s TATGAGTAGG TTTGCAGACT TGTAATTTTT CCCTTATTTG CAAGTTGCGT GCAACTAATA

wp-28s TATGAGTAGG TTTGCAGACT TGTAATTTTT CTCTTATTTG CAAGTTGCAT GCAACTAATA

wp-9s TATGAGTAGG TTTGCAGACT TGTAATTTTT CCCTTATTTG CAAGTTGCAT GCAACTAATA

wp-12s TATGAGTA-- ---------- ---------- ---------- ---------- ----------

wp-4s TATGAGTA-- ---------- ---------- ---------- ---------- ----------

wp-15s ---------- ---------- ---------- ---------- ---------- ----------

Consensus tatgagtagg tttgcagact tgtaattttt c.cttatttg caagttgc.t gcaactaata

2041 2100

wp-25s TGGCAGATAA AAAGAAGCAC AATGTGAAAC TTCTCTCTTA TTAATTTTTA TGATACATAG

wp-22s TGGCAGATAA AAAGAAGCAC AATGTGAAAC TTCTCTCTTA TTAATTTTTA TGATACATAG

wp-28s TGGCAGATAA AAAGAAGCAC AATGTGAAAC TTCTCTCTTA TTAATTTTTA TGATACATAG

wp-9s TGGCAGATAA AAAGAAGCAC AATGTGAAAC TTCTCTCTTA TTAATTTTTA TGATACATAG

wp-12s ---------- ---------- ---------- ---------- ---------- ----------

wp-4s ---------- ---------- ---------- ---------- ---------- ----------

wp-15s ---------- ---------- ---------- ---------- ---------- ----------

Consensus tggcagataa aaagaagcac aatgtgaaac ttctctctta ttaattttta tgatacatag

2101 2160

wp-25s ATTAAAATAT AATATGTCTT AGACAAATAT TAACTAAGAC CTTGTAACTC TAGATTATAT

wp-22s ATTAAAATAT AATATGTCTT AGACAAATAT TAACTAAGAC CTTGTAACTC TAGATTATAT

wp-28s ATTAAAATAT AATATGTCTT AGACAAATAT TAACTAAGAC CTTGTAACTC TAGATTATAT

wp-9s ATTAAAATAT AATATGTCTT AGACAAATAT TAACTAAGAC CTTGTAACTC TAGATTATAT

wp-12s ---------- ---------- ---------- ---------- ---------- ----------

wp-4s ---------- ---------- ---------- ---------- ---------- ----------

wp-15s ---------- ---------- ---------- ---------- ---------- ----------

Consensus attaaaatat aatatgtctt agacaaatat taactaagac cttgtaactc tagattatat

2161 2220

wp-25s GACCAGGAAC CATATAATAT TTCCTTTGGA TTTATTCCTT AAACTTTTTT AAAAATTGTT

wp-22s GACCAGGAAC CATATAATAT TTC-TTTGGA TTTATTCCTT AAACTTTTTT AAAAATTGTT

wp-28s GACCAGGAAC CATATAATAT TTC-TTTGGA TTTATTCCTT AAACTTTTTT AAAAATTGTT

wp-9s GACCAGGAAC CATATAATAT TTC-TTTGGA TTTATTCCTT AAACTTTTTT AAAAATTGTT

wp-12s ---------- ---------- ---------- ---------- ---------- ----------

wp-4s ---------- ---------- ---------- ---------- ---------- ----------

wp-15s ---------- ---------- ---------- ---------- ---------- ----------

Consensus gaccaggaac catataatat ttc.tttgga tttattcctt aaactttttt aaaaattgtt

2221 2280

wp-25s TTTAGTTCGT GAATTTTTTT TATTTATTTT TAGTTCTTTA ATTATATTTT GTCTTGGCTT

wp-22s TTTAGTTCGT GAATTTTTTT TATTTATTTT TAGTTCTTTA ATTATATTTT GTCTTGGCTT

wp-28s TTTAGTTCGT GAATTTTTTT TATTTATTTT TAGTTCTTTA ATTATATTTT GTCTTGGCTT

wp-9s TTTAGTTCGT GAATTTTTTT TATTTATTTT TAGTTCTTTA ATTATATTTT GTCTTGGCTT

wp-12s ---------- ---------- ---------- ---------- ---------- ----------

wp-4s ---------- ---------- ---------- ---------- ---------- ----------

wp-15s ---------- ---------- ---------- ---------- ---------- ----------

sConsensus tttagttcgt gaattttttt tatttatttt tagttcttta attatatttt gtcttggctt

2281 2340

wp-25s TTTTGTTTTT CTTGCAAATT TAAGCATGTC CAGCTTTCAT TTATACATTT GATGTCTTTT

wp-22s TTTTGTTTTT CTTGCAAATT TAAGCATGTC CAGCTTTCAT TTATACATTT GATGTCTTTT

wp-28s TTTTGTTTTT CTTGCAAATT TAAGCATGTC CAGCTTTCAT TTATACATTT GATGTCTTTT

wp-9s TTTTGTTTTT CTTGCAAATT TAAGCATGTC CAGCTTTCAT TTATACATTT GATGTCTTTT

wp-12s ---------- ---------- ---------- ---------- ---------- ----------

wp-4s ---------- ---------- ---------- ---------- ---------- ----------

wp-15s ---------- ---------- ---------- ---------- ---------- ----------

Consensus ttttgttttt cttgcaaatt taagcatgtc cagctttcat ttatacattt gatgtctttt

2341 2400

wp-25s TATTTTCTTA TTGTGAGTTA CTATTCAATC ACAAAGTTGC CAATTGCAAT ACCAGGTATA

wp-22s TATTTTCTTA TTGTGAGTTA CTATTCAATC ACAAAGTTGC CAATTGCAAT ACCAGGTATA

wp-28s TATTTTCTTA TTGTGAGTTA CTATTCAATC ACAAAGTTGC CAATTGCAAT ACCAGGTATA

wp-9s TATTTTCTTA TTGTGAGTTA CTATTCAATC ACAAAGTTGC CAATTGCAAT ACCAGGTATA

wp-12s ---------- ---------- ---------- ---------- ---------- --------TA

wp-4s ---------- ---------- ---------- ---------- ---------- ----------

wp-15s ---------- ---------- ---------- ---------- ---------- ----------

Consensus tattttctta ttgtgagtta ctattcaatc acaaagttgc caattgcaat accaggtata

2401 2460

wp-25s TAGGAACGGA AATCCTTATG GAATAGCTGA AGGTATTGTT TTCAGTATGC CATGCCGATC

wp-22s TAGGAACGGA AATCCTTATG GAATAGCTGA AGGTATTGTT TTCAGTATGC CATGCCGATC

wp-28s TAGGAACGGA AATCCTTATG GAATAGCTGA AGGTATTGTT TTCAGTATGC CATGCCGATC

wp-9s TAGGAACGGA AATCCTTATG GAATAGCTGA AGGTATTGTT TTCAGTATGC CATGCCGATC

wp-12s TAGGAACGGA AATCCTTATG GAATAGCTGA AGGTATTGTT TTCAGTATGC CATGCCGATC

wp-4s ---------- ---------- ---------- ---------- ---------- ----------

wp-15s ---------- ---------- ---------- ---------- ---------- ----------

Consensus taggaacgga aatccttatg gaatagctga aggtattgtt ttcagtatgc catgccgatc

2461 2520

wp-25s AAAGGTGATC ACCAAATCTA AGGAATTGGT AATGGTCTTA TTCCAAGTGT ACTGGATGTT

wp-22s AAAGGTGATC ACCAAATCTA AGGAATTGGT AATGGTCTCA TTCCAAGTGT ACTGGATGTT

wp-28s AAAGGTGATC ACCAAATCTA AGGAATTGGT AATGGTCTCA TTCCAAGTGT ACTGGATGTT

wp-9s AAAGGTGATC ACCAAATCTA AGGAATTGGT AATGGTCTCA TTCCAAGTGT ACTGGATGTT

wp-12s AAAGGTGATC ACCAAATCTA AGGAATTGGT AATGGTCTCA TTCCAAGTGT ACTGGATGTT

wp-4s ---------- ---------- ---------- ---------- ---------- ----------

wp-15s ---------- ---------- ---------- ---------- ---------- ----------

Consensus aaaggtgatc accaaatcta aggaattggt aatggtctca ttccaagtgt actggatgtt

2521 2580

wp-25s AATCTACTAG ATGAAGTTAT TCTACTAGAT GAAGTTATTC AGTATCTGAG CAATGGAAGG

wp-22s AATCTACTAG ATGAAGTTAT TCTACTGGAT GAAGTTATTC AGTATCTGAG CAATGGAAGG

wp-28s AATCTACTAG ATGAAGTTAT TCTACTAGAT GAAGTTATTC AGTATCTGAG CAATGGAAGG

wp-9s AATCTACTAG ATGAAGTTAT TCTACTAGAT GAAGTTATTC AGTATCTGAG CAATGGAAGG

wp-12s AATCTACTAG ATGAAGTTAT TCTACTAGAT GAAGTTATTC AGTATCTGAG CAATGGAAGG

wp-4s ---------- ---------- ---------- ---------- -GTATCTGAG CAATGGAAGG

wp-15s ---------- ---------- ---------- ---------- --TATCTGAG CAATGGAAGG

Consensus aatctactag atgaagttat tctactagat gaagttattc agTATCTGAG CAATGGAAGG

2581 2640

wp-25s TTCAAGAATG CTGATCACCA AGCGGTGGTG AACTCAAACC ATAGCCGTTT GTCCATAGCC

wp-22s TTCAAGAATG CTGATCACCA AGCGGTGGTG AACTCAAACC ATAGCCGTTT GTCCATAGCC

wp-28s TTCAAGAATG CTGATCACTA AGCGGTGGTG AACTCAAACC ATAGCCGTTT GTCCATAGCC

wp-9s TTCAAGAATG CTGATCACCA AGCGGTGGTG AACTCAAACC ATAGCCGTTT GTCCATAGCC

wp-12s TTCAAGAATG CTGATCACCA AGCGGTGGTG AACTCAAACC ATAGCCGTTT GTCCATAGCC

wp-4s TTCAAGAATG CTGATCACCA AGCGGTGGTG AACTCAAACC ATAGCCGTTT GTCCATAGCC

wp-15s TTCAAGAATG CTGATCACCA AGCGGTGGTG AACTCAGACC ATAGCCGTTT GTCCATAGCC

Consensus TTCAAGAATG CTGATCACcA AGCGGTGGTG AACTCAaACC ATAGCCGTTT GTCCATAGCC

2641 2700

wp-25s ACTTTTCAAA ACCCAGCACC AAATGCAACT GTTTACCCTC TGAAGATAAG AGAAGGAGAG

wp-22s ACTTTTCAAA ACCCAGCACC AAATGCAACT GTTTACCCTC TGAAGATAAG AGAAGGAGAG

wp-28s ACTTTTCAAA ACCCAGCACC AAATGCAACT GTTTACCCTC TGAAGATAAG AGAAGGAGAG

wp-9s ACTTTTCAAA ACCCAGCACC AAATGCAACT GTTTACCCTC TGAAGATAAG AGAAGGAGAG

wp-12s ACTTTTCAAA ACCCAGCACC AAATGCAACT GTTTACCCTC TGAAGATAAG AGAAGGAGAG

wp-4s ACTTTTCAAA ACCCAGCACC AAATGCAACT GTTTACCCTC TGAAGATAAG AGAAGGAGAG

wp-15s ACTTTTCAAA ACCCAGCACC AAATGCAACT GTTTACCCTC TGAAGATAAG AGAAGGAGAG

Consensus ACTTTTCAAA ACCCAGCACC AAATGCAACT GTTTACCCTC TGAAGATAAG AGAAGGAGAG

2701 2760

wp-25s AAGCCTGTGA TGGAGGAACC AATCACTTTT GCTGAAATGT ACAGGAGGAA GATGAGCAAG

wp-22s AAGCCTGTGA TGGAGGAACC AATCACTTTT GCTGAAATGT ACAGGAGGAA GATGAGCAAG

wp-28s AAGCCTGTGA TGGAGGAACC AATCACTTTT GCTGAAATGT ACAGGAGGAA GATGAGCAAG

wp-9s AAGCCTGTGA TGGAGGAACC AATCACTTTT GCTGAAATGT ACAGGAGGAA GATGAGCAAG

wp-12s AAGCCTGTGA TGGAGGAACC AATCACTTTT GCTGAAATGT ACAGGAGGAA GATGAGCAAG

wp-4s AAGCCTGTGA TGGAGGAACC AATCACTTTT GCTGAAATGT ACAGGAGGAA GATGAGCAAG

wp-15s AAGCCTGTGA TGGAGGAACC AATCACTTTT GCTGAAATGT ACAGGAGGAA GATGAGCAAG

Consensus AAGCCTGTGA TGGAGGAACC AATCACTTTT GCTGAAATGT ACAGGAGGAA GATGAGCAAG

2761 2820

wp-25s GACATTGAGA TTGCAAGGAT GAAGAAGCTG GCTAAGGAAA AGCATTTGCA GGACCTTGAG

wp-22s GACATTGAGA TTGCAAGGAT GAAGAAGCTG GCTAAGGAAA AGCATTTGCA GGACCTTGAG

wp-28s GACATTGAGA TTGCAAGGAT GAAGAAGCTG GCTAAGGAAA AGCATTTGCA GGACCTTGAG

wp-9s GACATTGAGA TTGCAAGGAT GAAGAAGCTG GCTAAGGAAA AGCATTTGCA GGACCTTGAG

wp-12s GACATTGAGA TTGCAAGGAT GAAGAAGCTG GCTAAGGAAA AGCATTTGCA GGACCTTGAG

wp-4s GACATTGAGA TTGCAAGGAT GAAGAAGCTG GCTAAGGAAA AGCATTTGCA GGACCTTGAG

wp-15s GACATTGAGA TTGCAAGGAT GAAGAAGCTG GCTAAGGAAA AGCATTTGCA GGACCTTGAG

Consensus GACATTGAGA TTGCAAGGAT GAAGAAGCTG GCTAAGGAAA AGCATTTGCA GGACCTTGAG

2821 2880

wp-25s AATGAAAAGC ATTTGCAAGA ACTTGATCAG AAGGCAAAAC TTGAGGCCAA GCCTTTGAAG

wp-22s AATGAAAAGC ATTTGCAAGA ACTTGATCAG AAGGCAAAAC TTGAGGCCAA GCCTTTGAAG

wp-28s AATGAAAAGC ATTTGCAAGA ACTTGATCAG AAGGCAAAAC TCGAGGCCAA GCCTTTGAAG

wp-9s AATGAAAAGC ATTTGCAAGA ACTTGATCAG AAGGCAAAAC TTGAGGCCAA GCCTTTGAAG

wp-12s AATGAAAAGC ATTTGCAAGA ACTTGATCAG AAGGCAAAAC TTGAGGCCAA GCCTTTGAAG

wp-4s AATGAAAAGC ATTTGCAAGA ACTTGATCAG AAGGCAAAAC TTGAGGCCAA GCCTTTGAAG

wp-15s AATGAAAAGC ATTTGCAAGA ACTTGATCAG AAGGCAAAAC TTGAGGCCAA GCCTTTGAAG

Consensus AATGAAAAGC ATTTGCAAGA ACTTGATCAG AAGGCAAAAC TtGAGGCCAA GCCTTTGAAG

2881 2940

wp-25s GAGATCCTTG CTTAATTAAT AATAATTACA TATGTATCAT TTGCATGCCC CCTTGGTGTT

wp-22s GAGATTCTTG CTTAATTAAT AATAATTACA TATGTATCAT TTGCATGCCC CCTTGGTGTT

wp-28s GAGATTCTTG CTTAATTAAT AATAATTACA TATGTATCAT TTGCATGCCC CCTTGGTGTT

wp-9s GAGATTCTTG CTTAATTAAT AATAATTACA TATGTATCAT TTGCATGCCC CCTTGGTGTT

wp-12s GAGATTCTTG CTTAATTAAT AATAATTACA TATGTATCAT TTGCATGCCC CCTTGGTGTT

wp-4s GAGATTCTTG CTTAATTAAT AATAATTACA TATGTATCAT TTGCATGCCC CCTTGGTGTT

wp-15s GAGATTCTTG CTTAATTAAT AATAATTACA TATGTATCAT TTGCATGCCC CCTTGGTGTT

Consensus GAGATtCTTG CTTAATTAAT AATAATTACA TATGTATCAT TTGCATGCCC CCTTGGTGTT

2941 3000

wp-25s TTTAGTATTT TTTAAGGGCC ATGAATTAAT AATAGTCCTT ACCTTTGTGC TTTTGTACGT

wp-22s TTTAGTATTT TTTAAGGGCC ATGAATTAAT AATAGTCCTT ACCTTTGTGC TTTTGTACGT

wp-28s TTTAGTATTT TTTAAGGGCC ATGAATTAAT AATAGTCCTT ACCTTTGTGC TTTTGTACGT

wp-9s TTTAGTATTT TTTAAGGGCC ATGAATTAAT AATAGTCCTT ACCTTTGTGC TTTTGTACGT

wp-12s TTTAGTATTT TTTAAGGGCC ATGAATTAAT AATAGTCCTT ACCTTTGTGC TTTTGTACGT

wp-4s TTTAGTATTT TTTAAGGGCC ATGAATTAAT AATAGTCCTT ACCTTTGTGC TTTTGTACGT

wp-15s TTTAGTATTT TTTAAGGGCC ATGAATTAAT AATAGTCCTT ACCTTTGTGC TTTTGTACGT

Consensus TTTAGTATTT TTTAAGGGCC ATGAATTAAT AATAGTCCTT ACCTTTGTGC TTTTGTACGT

3001 3060

wp-25s CTTATGATTT ATCCTTTGTG GGGATATCAT GTGTTGTGTT CAGTTGCCTA TGTCTTATTA

wp-22s CTTATGATTT ATCCTTTGTG GGGATATCAT GTGTTGTGTT CAGTTGCCTA TGTCTTATTA

wp-28s CTTATGATTT ATCCTTTGTG GGGATATCAT GTGTTGTGTT CAGTTGCCTA TGTCTTATTA

wp-9s CTTATGATTT ATCCTTTGTG GGGATATCAT GTGTTGTGTT CAGTTGCCTA TGTCTTATTA

wp-12s CTTATGATTT ATCCTTTGTG GGGATATCAT GTGTTGTGTT CAGTTGCCTA TGTCTTATTA

wp-4s CTTATGATTT ATCCTTTGTG GGGATATCAT GTGTTGTGTT CAGTTGCCTA TGTCTTATTA

wp-15s CTTATGATTT ATCCTTTGTG GGGATATCAT GTGTTGTGTT CAGTTGCCTA TGTCTTATTA

Consensus CTTATGATTT ATCCTTTGTG GGGATATCAT GTGTTGTGTT CAGTTGCCTA TGTCTTATTA

3061 3120

wp-25s GCTAGCTGGC TCATCTATGT ATACCTTATA TTTGCCTCTA TTATAAATGA AAATAAGTGG

wp-22s GCTAGCTGGC TCATCTATGT ATACCTTATA TTTGCCTCTA TTATAAATGA AAATAAGTGG

wp-28s GCTAGCTGGC TCATCTATGT ATACCTTATA TTTGCCTCTA TTATAAATGA AAATAAGTGG

wp-9s GCTAGCTGGC TCATCTATGT ATACCTTATA TTTGCCTCTA TTATAAATGA AAATAAGTGG

wp-12s GCTAGCTGGC TCATCTATGT ATACCTTATA TTTGCCTCTA TTATAAATGA AAATAAGTGG

wp-4s GCTAGCTGGC TCATCTATGT ATACCTTATA TTTGCCTCTA TTATAAATGA AAATAAGTGG

wp-15s GCTAGCTGGC TCATCTATGT ATACCTTATA TTTGCCTCTA TTATAAATGA AAATAAGTGG

Consensus GCTAGCTGGC TCATCTATGT ATACCTTATA TTTGCCTCTA TTATAAATGA AAATAAGTGG

3121 3130

wp-25s CACTGTCTTT

wp-22s CACTGTCTTT

wp-28s CACTGTCTTT

wp-9s CACTGTCTTT

wp-12s CACTGTCTTT

wp-4s CACTGTCTTT

wp-15s CACTGTCTTT

Consensus CACTGTCTTT
