# Supplementary material for: Novel exon combinations generated by alternative splicing of gene fragments mobilized by a CACTA transposon in Glycine max
Source: BMC Plant Biol. 2007 Jul 14;7:38. doi: 10.1186/1471-2229-7-38 (PMC1947982; doi:10.1186/1471-2229-7-38)
Supplement: Additional file 2 — Cotyledons wp RT-PCR cDNA sequence alignment. [file 1471-2229-7-38-S2.doc]

**Cotyledon *wp* RT-PCR cDNA clones**

1 60

wp-9c GCATTGCATT CTGCTATTTA ATTCCACTAC GTACACGCAC ATTCTCCTCA AAGACAACAA

wp-8c GCATTGCATT CTGCTATTTA ATTCCACTAC GTACACGCAC ATTCTCCTCA AAGACAACAA

wp-2c GCATTGCATT CTGCTATTTA ATTCCACTAC GTACACGCAC ATTCTCCTCA AAGACAACAA

wp-13c GCATTGCATT CTGCTATTTA ATTCCACTAC GTACACGCAC ATTCTCCTCA AAGACAACAA

wp-12c GCATTGCATT CTGCTATTTA ATTCCACTAC GTACACGCAC ATTCTCCTCA AAGACAACAA

wp-6c GCATTGCATT CTGCTATTTA ATTCCACTAC GTACACGCAC ATTCTCCTCA AAGACAACAA

Consensus GCATTGCATT CTGCTATTTA ATTCCACTAC GTACACGCAC ATTCTCCTCA AAGACAACAA

61 120

wp-9c TGGCACCAAC AGCCAAGACT CTGACTTACC TGGCCCAAGA GAAAACCCTA GAATCGAGCT

wp-8c TGGCACCAAC AGCCAAGACT CTGGCTTACC TGGCCCAGGA GAAAACCCTA GAATCGAGCT

wp-2c TGGCACCAAC AGCCAAGACT CTGACTTACC TGGCCCAGGA GAAAACCCTA GAATCGAGCT

wp-13c TGGCACCAAC AGCCAAGACT CTGACTTACC TGGCCCAGGA GAAAACCCTA GAATCGAGCT

wp-12c TGGCACCAAC AGCCAAGACT CTGACTTACC TGGCCCAGGA GAAAACCCTA GAATCGAGCT

wp-6c TGGCACCAAC AGCCAAGACT CTGACTTACC TGGCCCAGGA GAAAACCCTA GAATCGAGCT

Consensus TGGCACCAAC AGCCAAGACT CTGaCTTACC TGGCCCAgGA GAAAACCCTA GAATCGAGCT

121 180

wp-9c TCGTTCGGGA CGAGGAGGAG CGTCCCAAGG TTGCCTACAA CGAATTCAGC GACGAGATCC

wp-8c TCGTTCGGGA CGAGGAGGAG CGTCCCAAGG TTGCCTACAA CGAATTCAGC GACGAGATCC

wp-2c TCGTTCGGGA CGAGGAGGAG CGTCCCAAGG TTGCCTACAA CGAATTCAGC GACGAGATCC

wp-13c TCGTTCGGGA CGAGGAGGAG CGTCCCAAGG TTGCCTACAA CGAATTCAGC GACGAGATCC

wp-12c TCGTTCGGGA CGAGGAGGAG CGTCCCAAGG TTGCCTACAA CGAATTCAGC GACGAGATCC

wp-6c TCGTTCGGGA CGAGGAGGAG CGTCCCAAGG TTGCCTACAA CGAATTCAGC GACGAGATCC

Consensus TCGTTCGGGA CGAGGAGGAG CGTCCCAAGG TTGCCTACAA CGAATTCAGC GACGAGATCC

181 240

wp-9c CAGTGATTTC TCTTGCCGGA ATCGACGAGG TGGATGGACG CAGAAGAGAG ATTTGTGAGA

wp-8c CAGTGATTTC TCTTGCCGGA ATCGACGAGG TGGATGGACG CAGAAGAGAG ATTTGTGAGA

wp-2c CAGTGATTTC TCTTGCCGGA ATCGACGAGG TGGATGGACG CAGAAGAGAG ATTTGTGAGA

wp-13c CAGTGATTTC TCTTGCCGGA ATCGACGAGG TGGATGGACG CAGAAGAGAG ATTTGTGAGA

wp-12c CAGTGATTTC TCTTGCCGGA ATCGACGAGG TGGATGGACG CAGAAGAGAG ATTTGTGAGA

wp-6c CAGTGATTTC TCTTGCCGGA ATCGACGAGG TGGATGGACG CAGAAGAGAG ATTTGTGGGA

Consensus CAGTGATTTC TCTTGCCGGA ATCGACGAGG TGGATGGACG CAGAAGAGAG ATTTGTGaGA

241 300

wp-9c AGATCGTGGA GGCTTGCGAG AATTGGGGTA TATTCCAGGT TGTTGATCAC GGTGTGGATC

wp-8c AGATCGTGGA GGCTTGCGAG AATTGGGGCA TATTCCAGGT TGTTGATCAC GGTGTGGATC

wp-2c AGATCGTGGA GGCTTGCGAG AATTGGGGTA TATTCCAGGT TGTTGATCAC GGTGTGGATC

wp-13c AGATCGTGGA GGCTTGCGAG AATTGGGGTA TATTCCAGGT TGTTGATCAC GGTGTGGATC

wp-12c AGATCGTGGA GGCTTGCGAG AATTGGGGTA TATTCCAGGT TGTTGATCAC GGTGTGGATC

wp-6c AGATCGTGGA GGCTTGCGAG AATTGGGGTA TATTCCAGGT TGTTGATCAC GGTGTGGATC

Consensus AGATCGTGGA GGCTTGCGAG AATTGGGGtA TATTCCAGGT TGTTGATCAC GGTGTGGATC

301 360

wp-9c AACAACTCGT GGCCGAGATG ACCCGTCTCG CCAAAGAGTT CTTTGCTTTG CCACCGGACG

wp-8c AACAACTCGT GGCCGAGATG ACCCGTCTCG CCAAAGAGTT CTTTGCTTTG CCACCGGACG

wp-2c AACAACTCGT GGCCGAGATG ACCCGTCTCG CCAAAGAGTT CTTTGCTTTG CCACCGGACG

wp-13c AACAACTCGT GGCCGAGATG ACCCGTCTCG CCAAAGAGTT CTTTGCTTTG CCACCGGACG

wp-12c AACAACTCGT GGCCGAGATG ACCCGTCTCG CCAAAGAGTT CTTTGCTTTG CCACCGGACG

wp-6c AACAACTCGT GGCCGAGATG ACCCGTCTCG CCAAAGAGTT CTTTGCTTTG CCACCGGACG

Consensus AACAACTCGT GGCCGAGATG ACCCGTCTCG CCAAAGAGTT CTTTGCTTTG CCACCGGACG

361 420

wp-9c AGAAGCTTCG TTTTGATATG TCCGGCGCCA AAAAGGGTGG ATTCATTGTC TCCAGCCATC

wp-8c AGAAGCTTCG TTTTGATATG TCCGGCGCCA AAAAGGGTGG ATTCATTGTC TCCAGCCATC

wp-2c AGAAGCTTCG TTTTGATATG TCCGGCGCCA AAAAGGGTGG ATTCATTGTC TCCAGCCATC

wp-13c AGAAGCTTCG TTTTGATATG TCCGGCGCCA AAAAGGGTGG ATTCATTGTC TCCAGCCATC

wp-12c AGAAGCTTCG TTTTGATATG TCCGGCGCCA AAAAGGGTGG ATTCATTGTC TCCAGCCATC

wp-6c AGAAGCTTCG TTTTGATATG TCCGGCGCCA AAAAGGGTGG ATTCATTGTC TCCAGCCATC

Consensus AGAAGCTTCG TTTTGATATG TCCGGCGCCA AAAAGGGTGG ATTCATTGTC TCCAGCCATC

421 480

wp-9c TCCAAGGGGA ATCGGTGCAG GACTGGAGAG AAATAGTGAC ATACTTTTCG TACCCAAAAA

wp-8c TCCAAGGGGA ATCGGTGCAG GACTGGAGAG AAATAGTGAC ATACTTTTCG TACCCAAAAA

wp-2c TCCAAGGGGA ATCGGTGCAG GACTGGAGAG AAATAGTGAC ATACTTTTCG TACCCAAAAA

wp-13c TCCAAGGGGA ATCGGTGCAG GACTGGAGAG AAATAGTGAC ATACTTTTCG TACCCAAAAA

wp-12c TCCAAGGGGA ATCGGTGCAG GACTGGAGAG AAATAGTGAC ATACTTTTCG TACCCAAAA-

wp-6c TCCAAG---- ---------- -ACTGGAGAG AAATAGTGAC ATACTTTTCG TACCCAAAAA

Consensus TCCAAGggga atcggtgcag gACTGGAGAG AAATAGTGAC ATACTTTTCG TACCCAAAAa

481 540

wp-9c GAGAGAGGGA CTATTCAAGG TGGCCAGACA CGCCAGAAGG GTGGAGATCG GTGACTGAGG

wp-8c GAGAGAGGGA CTATTCAAGG TGGCCAGACA CGCCAGAAGG GTGGAGGTCG GTGACTGAGG

wp-2c GAGAGAGGGA CTATTCAAGG TGGCCAGACA CGCCAGAAGG GTGGAGATCG GTGACTGAGG

wp-13c GAGAGAGGGA CTATTCAAGG TGGCCAGACG CGCCAGAAGG GTGGAGATCG GTGACTGAGG

wp-12c GAGAGAGGGA CTATTCAAGG TGGCCAGACA CGCCAGAAGG GTGGAGATCG GTGACTGAGG

wp-6c GAGAGAGGGA CTATTCAAGG TGGCCAGACA CGCCAGAAGG GTGGAGATCG GTGACTGAGG

Consensus GAGAGAGGGA CTATTCAAGG TGGCCAGACa CGCCAGAAGG GTGGAGaTCG GTGACTGAGG

541 600

wp-9c AATACAGCGA CAAAGTAATG GGTCTAGCTT GCAAGCTCAT GGAGGTGTTG TCCGAAGCAA

wp-8c AATACAGCGA CAAAGTAATG GGTCTAGCTT GCAAGCTCAT GGAGGTGTTG TCCGAAGCAA

wp-2c AATACAGCGA CAAAGTAATG GGTCTAGCTT GCAAGCTCAT GGAGGTGTTG TCCGAAGCAA

wp-13c AATACAGCGA CAAAGTAATG GGTCTAGCTT GCAAGCTCAT GGAGGTGTTG TCCGAAGCAA

wp-12c AATACAGCGA CAAAGTAATG GGTCTAGCTT GCAAGCTCAT GGAGGTGTTG TCCGAAGCAA

wp-6c AATACAGCGA CAAAGTAATG GGTCTAGCTT GCAAGCTCAT GGAGGTGTTG TCCGAAGCAA

Consensus AATACAGCGA CAAAGTAATG GGTCTAGCTT GCAAGCTCAT GGAGGTGTTG TCCGAAGCAA

601 660

wp-9c TGGGGTTAGA GAAAGAGGGT TTAAGCAAAG CATGTGTTGA CATGGACCAG AAGGTGGTGG

wp-8c TGGGGTTAGA GAAAGAGGGT TTAAGCAAAG CATGTGTTGA CATGGACCAG AAGGTGGTGG

wp-2c TGGGGTTAGA GAAAGAGGGT TTAAGCAAAG CATGTGTTGA CATGGACCAG AAGGTGGTGG

wp-13c TGGGGTTAGA GAAAGAGGGT TTAAGCAAAG CATGTGTTGA CATGGACCAG AAGGTGGTGG

wp-12c TGGGGTTAGA GAAAGAGGGT TTAAGCAAAG CATGTGTTGA CATGGACCAG AAGGTGGTGG

wp-6c TGGGGTTAGA GAAAGAGGGT TTAAGCAAAG CATGTGTTGA CATGGACCAG AAGGTGGTGG

Consensus TGGGGTTAGA GAAAGAGGGT TTAAGCAAAG CATGTGTTGA CATGGACCAG AAGGTGGTGG

661 720

wp-9c TTAATTACTA CCCCAAATGC CCTCAACCTG ACCTCACTCT TGGCCTGAAG CGCCACACGG

wp-8c TTAATTACTA CCCCAAATGC CCTCAACCTG ACCTCACTCT TGGCCTGAAG CGCCACACGG

wp-2c TTAATTACTA CCCCAAATGC CCTCAACCTG ACCTCACTCT TGGCCTGAAG CGCCACACGG

wp-13c TTAATTACTA CCCCAAATGC CCTCAACCTG ACCTCACTCT TGGCCTGAAG CGCCACACGG

wp-12c TTAATTACTA CCCCAAATGC CCTCAACCTG ACCTCACTCT TGGCCTGAAG CGCCACACGG

wp-6c TTAATTACTA CCCCAAATGC CCTCAACCTG ACCTCACTCT TGGCCTGAAG CGCCACACGG

Consensus TTAATTACTA CCCCAAATGC CCTCAACCTG ACCTCACTCT TGGCCTGAAG CGCCACACGG

721 780

wp-9c ATCCGGGCAC TATCACCTTG CTGCTTCAGG ACCAAGTGGG TGGACTTCAA GCCACCAGGG

wp-8c ATCCGGGCAC TATCACCTTG CTGCTTCAGG ACCAAGTGGG TGGACTTCAA GCCACCAGGG

wp-2c ATCCGGGCAC TATCACCTTG CTGCTTCAGG ACCAAGTGGG TGGACTTCAA GCCACCAGGG

wp-13c ATCCGGGCAC TATCACCTTG CTGCTTCAGG ACCAAGTGGG TGGACTTCAA GCCACCAGGG

wp-12c ATCCGGGCAC TATCACCTTG CTGCTTCAGG ACCAAGTGGG TGGACTTCAA GCCACCAGGG

wp-6c ATCCGGGCAC TATCACCTTG CTGCTTCAGG ACCAAGTGGG TGGACTTCAA GCCACCAGGG

Consensus ATCCGGGCAC TATCACCTTG CTGCTTCAGG ACCAAGTGGG TGGACTTCAA GCCACCAGGG

781 840

wp-9c ACAATGGCAA AACATGGATC ACCGTTCAGC CTGTGGAGGC TGCCTTCGTC GTCAATCTTG

wp-8c ACAATGGCAA AACATGGATC ACCGTTCAGC CTGTGGAGGC TGCCTTCGTC GTCAATCTTG

wp-2c ACAATGGCAA AACATGGATC ACCGTTCAGC CTGTGGAGGC TGCCTTCGTC GTCAATCTTG

wp-13c ACAATGGCAA AATATGGATC ACCGTTCAGC CTGTGGAGGC TGCCTTCGTC GTCAATCTTG

wp-12c ACAATGGCAA AACATGGATC ACCGTTCAGC CTGTGGAGGC TGCCTTCGTC GTCAATCTTG

wp-6c ACAATGGCAA AACATGGATC ACCGTTCAGC CTGTGGAGGC TGCCTTCGTC GTCAATCTTG

Consensus ACAATGGCAA AAcATGGATC ACCGTTCAGC CTGTGGAGGC TGCCTTCGTC GTCAATCTTG

841 900

wp-9c GAGATCATGC TCATGTAGAC CACGATGGCA TTTTTGTAAT TCGAAGACTT TATTCTAAGA

wp-8c GAGATCATGC TCAT----AC CACGATGGCA TTTTTGTAAT TCGAAGACTT TATTCTAAGA

wp-2c GAGATCATGC TCATGTAGAC CACGATGGCA TTTTTGTAAT TCGAAGACTT TATTCTAAGA

wp-13c GAGATCATGC TCAT------ ---------- ---------- ---------- ----------

wp-12c GAGATCATGC TCAT------ ---------- ---------- ---------- ----------

wp-6c GAGATCATGC TCAT------ ---------- ---------- ---------- ----------

Consensus GAGATCATGC TCAT...... .......... .......... .......... ..........

901 960

wp-9c CGAGTGTCAT TAGAAAACGT CGTTGTTAGT TTAGAAAAAA AAAAGTTATA CGCGTTCTGA

wp-8c CGAGTGTCAT TAGAAAACGT CGTTGTTAGT TTAGAAAAAA AAAAGTTATA CGCGTTCTGA

wp-2c CGAGTGTCAT TAGAAAACGT CGTTGTTAGT TTAGAAAAAA AAAAGTTATA CGCGTTCTGA

wp-13c ---------- ---------- ---------- ---------- ---------- ----------

wp-12c ---------- ---------- ---------- ---------- ---------- ----------

wp-6c ---------- ---------- ---------- ---------- ---------- ----------

Consensus .......... .......... .......... .......... .......... ..........

961 1020

wp-9c ACCCTAAGCA AGGTCACAAC CTCGAAGCTG AGTTCTTTCT ACTGTGCTGC ATCAACAGTT

wp-8c ACCTTAAGCA AGGTCACAAC CTCGAAGCTG AGTTCTTTCT ACTGTGCTGC ATCAACAGTT

wp-2c ACCCTAAGCA AGGTCACAAC CTCGAAGCTG AGTTCTTTCT ACTGTGCTGC ATCAACAGTT

wp-13c ---------- ---------- ---------- ---------- ---------- --------TT

wp-12c ---------- ---------- ---------- ---------- ---------- --------TT

wp-6c ---------- ---------- ---------- ---------- ---------- --------TT

Consensus .......... .......... .......... .......... .......... ........TT

1021 1080

wp-9c ATACGACGAC AATGACTGTG ACCTAAAATC ACCTCTGAAG AAACCCGATT CCGCATCCAC

wp-8c ATACGACGAC AATGACTGTG ACCTAAAATC ACCTCAGAAG AAACCCGATT CCGCATCCAC

wp-2c ATACGACGAC AATGACTGTG ACCTAAAATC ACCTCTGAAG AAACCCGATT CCGCATCCAC

wp-13c ATACGACGAC AATGACTGTG ACCTAAAATC ACCTCTGAAG AAACCCGATT CCGCATCCAC

wp-12c ATACGACGAC AATGACTGTG ACCTAAAATC ACCTCTGAAG AAACCCGATT CCGCATCCAC

wp-6c ATACGACGAC AATGACTGTG ACCTAAAATC ACCTCTGAAG GAACCCGATT CCGCATCCAC

Consensus ATACGACGAC AATGACTGTG ACCTAAAATC ACCTCtGAAG aAACCCGATT CCGCATCCAC

1081 1140

wp-9c CCCCAGTCCC ACCGCGTGCG GCGTCAAAGA GGATTTCTCC GTGCTCACCA ACGACGAAGA

wp-8c CCCCAGTCCC ACCGCGTGCG GCGTCAAAGA GGATTTCTCC GTGCTCACCA ACGACGAAGA

wp-2c CCCCAGTCCC ACCGCGTGCG GCGTCAAAGA GGATTTCTCC GTGCTCACCA ACGACGAAGA

wp-13c CCCCAGTCCC ACCGCGTGCG GCGTCAAAGA GGATTTCTCC GTGCTCACCA ACGACGAAGA

wp-12c CCCCAGTCCC ACCGCGTGCG GCGTCAAAGA GGATTTCTCC GTGCTCACCA ACGACGAAGA

wp-6c CCCCAGTCCC ACCGCGTGCG GCGTCAAAGA GGATTTCTCC GTGCTCACCA ACGACGAAGA

Consensus CCCCAGTCCC ACCGCGTGCG GCGTCAAAGA GGATTTCTCC GTGCTCACCA ACGACGAAGA

1141 1200

wp-9c AGAAGAAGAT GTAATTGCCG GTATCCGAAA TGATTTCACA GAGTTAGTAG CAAGTTCAAG

wp-8c AGAAGAAGAT GTAATTGCCG GTATCCGAAA TGATTTCACA GAGTTAGTAG CAAGTTCAAG

wp-2c AGAAGAAGAT GTAATTGCCG GTATCCGAAA TGATTTCACA GAGTTAGTAG CAAGTTCAAG

wp-13c AGAAGAAGAT GTAATTGCCG GTATCCGAAA TGATTTCACA GAGTTAGTAG CAAGTTCAAG

wp-12c AGAAGAAGAT GTAATTGCCG GTATCCGAAA TGATTTCACA GAGTTAGTAG CAAGTTCAAG

wp-6c AGAAGAAGAT GTAATTGCCG GTATCCGAAA TGATTTCACA GAGTTAGTAG CAAGTTCAAG

Consensus AGAAGAAGAT GTAATTGCCG GTATCCGAAA TGATTTCACA GAGTTAGTAG CAAGTTCAAG

1201 1260

wp-9c AACGGGATCT TCAAAATCTG CGAGGCTCAC TGTAACTCCT AACTTCCCAG ATTGTCATCT

wp-8c AACGGGATCT TCAAAATCTG CGAGGCTCAC TGTAACTCCT AACTTCCCAG ATTGTCATCT

wp-2c AACGGGATCT TCAAAATCTG CGAGGCTCAC TGTAACTCCT AACTTCCCAG ATTGTCATCT

wp-13c AACGGGATCT TCAAAATCTG CGAGGCTCAC TGTAACTCCT AACTTCCCAG ATTGTCATCT

wp-12c AACGGGATCT TCAAAATCTG CGAGGCTCAC TGTAACTCCT AACTTCCCAG ATTGTCATCT

wp-6c AACGGGATCT TCAAAATCTG CGAGGCTCAC TGTAACTCCT AACTTCCCAG ATTGTCATCT

Consensus AACGGGATCT TCAAAATCTG CGAGGCTCAC TGTAACTCCT AACTTCCCAG ATTGTCATCT

1261 1320

wp-9c TCCTCTTCAG TCATCTACTT TTCCTCTTTT CTCTCCGATG ATTGTTGGAA CTCAAAGAGC

wp-8c TCCTCTTCAG TCATCTACTT TTCCTCTTTT CTCTCCGATG ATTGTTGGAA CTCAAAGAGC

wp-2c TCCTCTTCAG TCATCTACTT TTCCTCTTTT CTCTCCGATG ATTGTTGGAA CTCAAAGAGC

wp-13c TCCTCTTCAG TCATCTACTT TTCCTCTTTT CTCTCCGATG ATTGTTGGAA CTCAAAGAGC

wp-12c TCCTCTTCAG TCATCTACTT TTCCTCTTTT CTCTCCGATG ATTGTTGGAA CTCAAAGAGC

wp-6c TCCTCTTCAG TCATCTACTT TTCCTCTTTT CTCTCCGATG ATTGTTGGAA CTCAAAGAGC

Consensus TCCTCTTCAG TCATCTACTT TTCCTCTTTT CTCTCCGATG ATTGTTGGAA CTCAAAGAGC

1321 1380

wp-9c TTCTTTGATG GAACAGTACG AAAAGGTTGA GAAGATAGGG TCGCGACCGC GTCACCAACG

wp-8c TTCTTTGATG GAACAGTACG AAAAGGTTGA GAAGATAGGG TCGCGACCGC GTCACCAACG

wp-2c TTCTTTGATG GAACAGTACG AAAAGGTTGA GAAGATAGGG TCGCGACCGC GTCACCAACG

wp-13c TTCTTTGATG GAACAGTACG AAAAGGTTGA GAAGATAGGG TCGCGACCGC GTCACCAACG

wp-12c TTCTTTGATG GAACAGTACG AAAAGGTTGA GAAGATAGGG TCGCGACCGC GTCACCAACG

wp-6c TTCTTTGATG GAACAGTACG AAAAGGTTGA GAAGATAGGG TCGCGACCGC GTCACCAACG

Consensus TTCTTTGATG GAACAGTACG AAAAGGTTGA GAAGATAGGG TCGCGACCGC GTCACCAACG

1381 1440

wp-9c AGACCATCGC GTTGAAGAAG ATTCGCCTCG AGCAGGAGGA TGAGGGGGTT CCCAGCACCG

wp-8c AGACCATCGC GTTGAAGAAG ATTCGCCTCG AGCAGGAGGA TGAGGGGGTT CCCAGCACCG

wp-2c AGACCATCGC GTTGAAGAAG ATTCGCCTCG AGCAGGAGGA TGAGGGGGTT CCCAGCACCG

wp-13c AGACCATCGC GTTGAAGAAG ATTCGCCTCG AGCAGGAGGA TGAGGGGGTT CCCAGCACCG

wp-12c AGACCATCGC GTTGAAGAAG ATTCGCCTCG AGCAGGAGGA TGAGGGGGTT CCCAGCACCG

wp-6c AGACCATCGC GTTGAAGAAG ATTCGCCTCG AGCAGGAGGA TGAGGGGGTT CCCAGCACCG

Consensus AGACCATCGC GTTGAAGAAG ATTCGCCTCG AGCAGGAGGA TGAGGGGGTT CCCAGCACCG

1441 1500

wp-9c CCATTCACGA GATTTCTCTC TTGAAAGAAA TGCAGCACAG GAACATTGTT AGGTTGTAGG

wp-8c CCGTTCACGA GATTTCTCTC TTGAAAGAAA TGCAGCACAG GAACATTGTT AGGTTGTAGG

wp-2c CCATTCACGA GATTTCTCTC TTGAAAGAAA TGCAGCACAG GAACATTGTT AGGTTGTAGG

wp-13c CCATTCACGA GATTTCTCTC TTGAAAGAAA TGCAGCACAG GAACATTGTT AGGTTGTAGG

wp-12c CCATTCACGA GATTTCTCTC TTGAAAGAAA TGCAGCACAG GAACATTGTT AGGTTGTAGG

wp-6c CCATTCACGA GATTTCTCTC TTGAAAGAAA TGCAGCACAG GAACATTGTT AGGTTGTAGG

Consensus CCaTTCACGA GATTTCTCTC TTGAAAGAAA TGCAGCACAG GAACATTGTT AGGTTGTAGG

1501 1560

wp-9c ATGTAGTGCA CGATGAGAAG AGTTTGTATC TGGTACAATT GGTTTCTGAG GGAAGACAAA

wp-8c ATGTAGTGCA CGATGAGAAG AGTTTGTATC TGGTACAATT GGTTTCTGAG GGAAGACAAA

wp-2c ATGTAGTGCA CGATGAGAAG AGTTTGTATC TGGTACAATT GGTTTCTGAG GGAAAACAAA

wp-13c ATGTAGTGCA CGATGAGAAG AGTTTGTATC TGGTACAATT GGTTTCTGAG GGAAGACAAA

wp-12c ATGTAGTGCA CGATGAGAAG AGTTTGTATC TGGTACAATT GGTTTCTGAG GGAAGACAAA

wp-6c ATGTAGTGCA CGATGAGAAG AGTTTGTATC TGGTACAATT GGTTTCTGAG GGAAGACAAA

Consensus ATGTAGTGCA CGATGAGAAG AGTTTGTATC TGGTACAATT GGTTTCTGAG GGAAgACAAA

1561 1620

wp-9c GGTAGTTAGA TAATAAATTC AAGGACTTTG TTGCAAGGTT GAAATGGT-- ----------

wp-8c GGTAGTTAGA TAATAAATTC AAGGACTTTG TTGCAAGGTT GAAATGGT-- ----------

wp-2c GGTAGTTAGA TAATAAATTC AAGGACTTTG TTGCAAGGTT GAAATGGT-- ----------

wp-13c GGTAGTTAGA TAATAAATTC AAGGACTTTG TTGCAAGGTT GAAATGGTGT AATAGTTCTA

wp-12c GGTAGTTAGA TAATAAATTC AAGGACTTTG TTGCAAGGTT GAAATGGT-- ----------

wp-6c G--------- ---------- ---------- ---------- ---------- ----------

Consensus Ggtagttaga taataaattc aaggactttg ttgcaaggtt gaaatggt.. ..........

1621 1680

wp-9c ---------- ---------- ---------- ---------- ---------- ----------

wp-8c ---------- ---------- ---------- ---------- ---------- ----------

wp-2c ---------- ---------- ---------- ---------- ---------- ----------

wp-13c ACTTCTCTAT CCTTGAATTC TTGCTTCATT ACTCTCTTTT CTAATGTGAT ATGTGGCATT

wp-12c ---------- ---------- ---------- ---------- ---------- ----------

wp-6c ---------- ---------- ---------- ---------- ---------- ----------

Consensus .......... .......... .......... .......... .......... ..........

1681 1740

wp-9c ---------- ---------- ---------- ---------- ---------- --------TG

wp-8c ---------- ---------- ---------- ---------- ---------- --------TG

wp-2c ---------- ---------- ---------- ---------- ---------- --------TG

wp-13c AGAATCATTT TCAATATTTG GCTGAATACC TAGAACACTA TGCAATTTGT TATAACAGTG

wp-12c ---------- ---------- ---------- ---------- ---------- --------TG

wp-6c ---------- ---------- ---------- ---------- ---------- --------TG

Consensus .......... .......... .......... .......... .......... ........TG

1741 1800

wp-9c AGGCTGGAGA ACTTTATTCA AAGAAGCTTG CCAAGTTTGT TGGAAAGCGT CTCAAATCAG

wp-8c AGGCTGGAGA ACTTTATTCA AAGAAGCTTG CCAAGTTTGT TGGAAAGCGT CTCAAATCAG

wp-2c AGGCTGGAGA ACTTTATTCA AAGAAGCTTG CCAAGTTTGT TGGAAAGCGT CTCAAATCAG

wp-13c AGGCTGGAGA ACTTTATTCA AAGAAGCTTG CCAAGTTTGT TGGAAAGCGT CTCAAATCAG

wp-12c AGGCTGGAGA ACTTTATTCA AAGAAGCTTG CCAAGTTTGT TGGAAAGCGT CTCAAATCAG

wp-6c AGGCTGGAGA ACTTTATTCA AAGAAGCTTG CCAAGTTTGT TGGAAAGCGT CTCAAATCAG

Consensus AGGCTGGAGA ACTTTATTCA AAGAAGCTTG CCAAGTTTGT TGGAAAGCGT CTCAAATCAG

1801 1860

wp-9c AATGGGCTGC TTCTATATGG ACTAGTACAC TGCAACGAAC AATTCTGACA GCCACTCCAA

wp-8c AATGGGCTGC TTCTATATGG ACTAGTACAC TGCAACGAAC AATTCTGACA GCCACTCCAA

wp-2c AATGGGCTGC TTCTATATGG ACTAGTACAC TGCAACGAAC AATTCTGACA GCCACTCCAA

wp-13c AATGGGCTGC TTCTATATGG ACTAGTACAC TGCAACGAAC AATTCTGACA GCCACTCCAA

wp-12c AATGGGCTGC TTCTATATGG ACTAGTACAC TGCAACGAAC AATTCTGACA GCCACTCCAA

wp-6c AACGGGCTGC TTCTATATGG ACTAGTACAC TGCAACGAAC AATTCTGACA GCCACTCCAA

Consensus AAtGGGCTGC TTCTATATGG ACTAGTACAC TGCAACGAAC AATTCTGACA GCCACTCCAA

1861 1920

wp-9c TTATTGGATT TCCCAAGATA CAATGGCGTG CACTTGATGA GATAAACGCA GGGGTGTGTG

wp-8c TTATTGGATT TCCCAAGATA CAATGGCGTG CACTTGATGA GATAAACGCA GGGGTGTGTG

wp-2c TTATTGGATT TCCCAAGATA CAATGGCGTG CACTTGATGA GATAAACGCA GGGGTGTGTG

wp-13c TTACTGGATT TCCCAAGATA CAATGGCGTG CACTTGATGA GATAAACGCA GGGGTGTGTG

wp-12c TTATTGGATT TCCCAAGATA CAATGGCGTG CACTTGATGA GATAAACGCA GGGGTGTGTG

wp-6c TTATTGGATT TCCCAAGATA CAATGGCGTG CACTTGATGA GATAAACGCA GGGGTGTGTG

Consensus TTAtTGGATT TCCCAAGATA CAATGGCGTG CACTTGATGA GATAAACGCA GGGGTGTGTG

1921 1980

wp-9c ATGGTATGGC ATATGCAGAA ATCAAGAAAA ACATGCCAGA GGAGTATGAG TAGGTTTGCA

wp-8c ATGGTATGGC ATATGCAGAA ATCAAGAAAA ACATGCCAGA GGAGTATGAG TAGGTTTGCA

wp-2c ATGGTATGGC ATATGCAGAA ATCAAGAAAA ACATGCCAGA GGAGTATGAG TAGGT-----

wp-13c ATGGTATGGC ATATGCAGAA ATCAAGAAAA ACATGCCAGA GGAGTATGAG TA--------

wp-12c ATGGTATGGC ATATGCAGAA ATCAAGAAAA ACATGCCAGA GGAGTATGAG TA--------

wp-6c ATGGTATGGC ATATGCAGAA ATCAAGAAAA ACATGCCAGA GGAGTATGAG TA--------

Consensus ATGGTATGGC ATATGCAGAA ATCAAGAAAA ACATGCCAGA GGAGTATGAG TA........

1981 2040

wp-9c GACTTGTAAT TTTTCTCTTA TTTGCAAGTT GCATGCAACT AATATGGCAG ATAAAAAGAA

wp-8c GACTTGTAAT TTTTCTCTTA TTTGCAAGTT GCATGCAACT AATATGGCAG ATAAAAAGAA

wp-2c ---------- ---------- ---------- ---------- ---------- ----------

wp-13c ---------- ---------- ---------- ---------- ---------- ----------

wp-12c ---------- ---------- ---------- ---------- ---------- ----------

wp-6c ---------- ---------- ---------- ---------- ---------- ----------

Consensus .......... .......... .......... .......... .......... ..........

2041 2100

wp-9c GCACAATGTG AAACTTCTCT CTTATTAATT TTTATGATAC ATAGATTAAA ATATGATATG

wp-8c GCACAATGTG AAACTTCTCT CTTATTAATT TTTATGATAC ATAGATTAAA ATATAATATG

wp-2c ---------- ---------- ---------- ---------- ---------- ----------

wp-13c ---------- ---------- ---------- ---------- ---------- ----------

wp-12c ---------- ---------- ---------- ---------- ---------- ----------

wp-6c ---------- ---------- ---------- ---------- ---------- ----------

Consensus .......... .......... .......... .......... .......... ..........

2101 2160

wp-9c TCTTAGACAA ATATTAACTA AGACCTTGTA ACTCTAGATT ATATGACCAG GAACCATATA

wp-8c TCTTAGACAA ATATTAACTA AGACCTTGTA ACTCTAGATT ATATGACCAG GAACCATATA

wp-2c ---------- ---------- ---------- ---------- ---------- ----------

wp-13c ---------- ---------- ---------- ---------- ---------- ----------

wp-12c ---------- ---------- ---------- ---------- ---------- ----------

wp-6c ---------- ---------- ---------- ---------- ---------- ----------

Consensus .......... .......... .......... .......... .......... ..........

2161 2220

wp-9c ATATTTCCTT TGGATTTATT CCTTAAACTT TTTTAAAAAT TGTTTTTAGT TCGTGAATTT

wp-8c ATATTTC-TT TGGATTTATT CCTTAAACTT TTTTAAAAAT TGTTTTTAGT TCGTGAATTT

wp-2c ---------- ---------- ---------- ---------- ---------- ----------

wp-13c ---------- ---------- ---------- ---------- ---------- ----------

wp-12c ---------- ---------- ---------- ---------- ---------- ----------

wp-6c ---------- ---------- ---------- ---------- ---------- ----------

Consensus .......... .......... .......... .......... .......... ..........

2221 2280

wp-9c TTTTTATTTA TTTTTAGTTC TTTAATTATA TTTTGTCTTG GCTTTTTTGT TTTTCTTGCA

wp-8c TTTTTATTTA TTTTTAGTTC TTTAATTATA TTTTGTCTTG GCTTTTTTGT TTTTCTTGCA

wp-2c ---------- ---------- ---------- ---------- ---------- ----------

wp-13c ---------- ---------- ---------- ---------- ---------- ----------

wp-12c ---------- ---------- ---------- ---------- ---------- ----------

wp-6c ---------- ---------- ---------- ---------- ---------- ----------

Consensus .......... .......... .......... .......... .......... ..........

2281 2340

wp-9c AATTTAAGCA TGTCCAGCTT TCATTTATAC ATTTGATGTC TTTTTATTTT CTTATTGTGA

wp-8c AATTTAAGCA TGTCCAGCTT TCATTTATAC ATTTGATGTC TTTTTATTTT CTTATTGTGA

wp-2c ---------- ---------- ---------- ---------- ---------- ----------

wp-13c ---------- ---------- ---------- ---------- ---------- ----------

wp-12c ---------- ---------- ---------- ---------- ---------- ----------

wp-6c ---------- ---------- ---------- ---------- ---------- ----------

Consensus .......... .......... .......... .......... .......... ..........

2341 2400

wp-9c GTTACTATTC AATCACAAAG TTGCCAATTG CAATACCAGG TATATAGGAA CGGAAATCCT

wp-8c GTTACTATTC AATCACAAAG TTGCCAATTG CAATACCAGG TATATAGGAA CGGAAATCCT

wp-2c ---------- ---------- ---------- ---------- -ATATAGGAA CGGAAATCCT

wp-13c ---------- ---------- ---------- ---------- --TATAGGAA CGGAAATCCT

wp-12c ---------- ---------- ---------- ---------- --TATAGGAA CGGAAATCCT

wp-6c ---------- ---------- ---------- ---------- --TATAGGAA CGGAAATCCT

Consensus .......... .......... .......... .......... ..TATAGGAA CGGAAATCCT

2401 2460

wp-9c TATGGAATAG CTGAAGGTAT TGTTTTCAGT ATGCCATGCC GATCAAAGGT GATCACCAAA

wp-8c TATGGAATAG CTGAAGGTAT TGTTTTCAGT ATGCCATGCC GATCAAAGGT GATCACCAAA

wp-2c TATGGAATAG CTGAAGGTAT TGTTTTCAGT ATGCCATGCC GATCAAAGGT GATCACCAAA

wp-13c TATGGAATAG CTGAAGGTAT TGTTTTCAGT ATGCCATGCC GATCAAAGGT GATCACCAAA

wp-12c TATGGAATAG CTGAAGGTAT TGTTTTCAGT ATGCCATGCC GATCAAAGGT GATCACCAAA

wp-6c TATGGAATAG CTGAAGGTAT TGTTTTCAGT ATGCCATGCC GATCAAAGGT GATCACCAAA

Consensus TATGGAATAG CTGAAGGTAT TGTTTTCAGT ATGCCATGCC GATCAAAGGT GATCACCAAA

2461 2520

wp-9c TCTAAGGAAT TGGTAATGGT CTCATTCCAA GTGTACTGGA TGTTAATCTA CTAGATGAAG

wp-8c TCTAAGGAAT TGGTAATGGT CTCATTCCAA GTGTACTGGA TGTTAATCTA CTAGATGAAG

wp-2c TCTAAGGAAT TGGTAATGGT CTCATTCCAA GTGTACTGGA TGTTAATCTA CTAGATGAAG

wp-13c TCTAAGGAAT TGGTAATGGT CTCATTCCAA GTGTACTGGA TGTTAATCTA CTAGATGAAG

wp-12c TCTAAGGAAT TGGTAATGGT CTCATTCCAA GTGTACTGGA TGTTAATCTA CTAGATGAAG

wp-6c TCTAAGGAAT TGGTAATGGT CTCATTCCAA GTGTACTGGA TGTTAATCTA CTAGATGAAG

Consensus TCTAAGGAAT TGGTAATGGT CTCATTCCAA GTGTACTGGA TGTTAATCTA CTAGATGAAG

2521 2580

wp-9c TTATTCTACT AGATGAAGTT ATTCAGTATC TGAGCAATGG AAGGTTCAAG AATGCTGATC

wp-8c TTATTCTACT AGATGAAGTT ATTCAGTATC TGAGCAATGG AAGGTTCAAG AATGCTGATC

wp-2c TTATTCTACT AGATGAAGTT ATTCAGTATC TGAGCAATGG AAGGTTCAAG AATGCTGATC

wp-13c TTATTCTACT AGATGAAGTT ATTCAGTATC TGAGCAATGG AAGGTTCAAG AATGCTGATC

wp-12c TTATTCTACT AGATGAAGTT ATTCAGTATC TGAGCAATGG AAGGTTCAAG AATGCTGATC

wp-6c TTATTCTACT AGATGAAGTT ATTCAGTATC TGAGCAATGG AAGGTTCAAG AATGCTGATC

Consensus TTATTCTACT AGATGAAGTT ATTCAGTATC TGAGCAATGG AAGGTTCAAG AATGCTGATC

2581 2640

wp-9c ACCAAGCGGT GGTGAACTCA AACCATAGCC GTTTGTCCAT AGCCACTTTT CAAAACCCAG

wp-8c ACCAAGCGGT GGTGAACTCA AACCATAGCC GTTTGTCCAT AGCCACTTTT CAAAACCCAG

wp-2c ACCAAGCGGT GGTGAACTCA AACCATAGCC GTTTGTCCAT AGCCACTTTT CAAAACCCAG

wp-13c ACCAAGCGGT GGTGAACTCA AACCATAGCC GTTTGTCCAT AGCCACTTTT CAAAACCCAG

wp-12c ACCAAGCGGT GGTGAACTCA AACCATAGCC GTTTGTCCAT AGCCACTTTT CAAAACCCAG

wp-6c ACCAAGCGGT GGTGAACTCA AACCATAGCC GTTTGTCCAT AGCCACTTTT CAAAACCCAG

Consensus ACCAAGCGGT GGTGAACTCA AACCATAGCC GTTTGTCCAT AGCCACTTTT CAAAACCCAG

2641 2700

wp-9c CACCAAATGC AACTGTTTAC CCTCTGAAGA TAAGAGAAGG AGAGAAGCCT GTGATGGAGG

wp-8c CACCAAATGC AACTGTTTAC CCTCTGAAGA TAAGAGAAGG AGAGAAGCCT GTGATGGAGG

wp-2c CACCAAATGC AACTGTTTAC CCTCTGAAGA TAAGAGAAGG AGAGAAGCCT GTGATGGAGG

wp-13c CACCAAATGC AACTGTTTAC CCTCTGAAGA TAAGAGAAGG AGAGAAGCCT GTGATGGAGG

wp-12c CACCAAATGC AACTGTTTAC CCTCTGAAGA TAAGAGAAGG AGAGAAGCCT GTGATGGAGG

wp-6c CACCAAATGC AACTGTTTAC CCTCTGAAGA TAAGAGAAGG AGAGAAGCCT GTGATGGAGG

Consensus CACCAAATGC AACTGTTTAC CCTCTGAAGA TAAGAGAAGG AGAGAAGCCT GTGATGGAGG

2701 2760

wp-9c AACCAATCAC TTTTGCTGAA ATGTACAGGA GGAAGATGAG CAAGGACATT GAGATTGCAA

wp-8c AACCAATCAC TTTTGCTGAA ATGTACAGGA GGAAGATGAG CAAGGACATT GAGATTGCAA

wp-2c AACCAATCAC TTTTGCTGAA ATGTACAGGA GGAAGATGAG CAAGGACATT GAGATTGCAA

wp-13c AACCAATCAC TTTTGCTGAA ATGTACAGGA GGAAGATGAG CAAGGACATT GAGATTGCAA

wp-12c AACCAATCAC TTTTGCTGAA ATGTACAGGA GGAAGATGAG CAAGGACATT GAGATTGCAA

wp-6c AACCAATCAC TTTTGCTGAA ATGTACAGGA GGAAGATGAG CAAGGACATT GAGATTGCAA

Consensus AACCAATCAC TTTTGCTGAA ATGTACAGGA GGAAGATGAG CAAGGACATT GAGATTGCAA

2761 2820

wp-9c GGATGAAGAA GCTGGCTAAG GAAAAGCATT TGCAGGACCT TGAGAATGAA AAGCATTTGC

wp-8c GGATGAAGAA GCTGGCTAAG GAAAAGCATT TGCAGGACCT TGAGAATGAA AAGCATTTGC

wp-2c GGATGAAGAA GCTGGCTAAG GAAAAGCATT TGCAGGACCT TGAGAATGAA AAGCATTTGC

wp-13c GGATGAAGAA GCTGGCTAAG GAAAAGCATT TGCAGGACCT TGAGAATGAA AAGCATTTGC

wp-12c GGATGAAGAA GCTGGCTAAG GAAAAGCATT TGCAGGACCT TGAGAATGAA AAGCATTTGC

wp-6c GGATGAAGAA GCTGGCTAAG GAAAAGCATT TGCAGGACCT TGAGAATGAA AAGCATTTGC

Consensus GGATGAAGAA GCTGGCTAAG GAAAAGCATT TGCAGGACCT TGAGAATGAA AAGCATTTGC

2821 2880

wp-9c AAGAACTTGA TCAGAAGGCA AAACTTGAGG CCAGGCCTTT GAAGGAGATT CTTGCTTAAT

wp-8c AAGAACTTGA TCAGAAGGCA AAACTTGAGG CCAAGCCTTT GAAGGAGATT CTTGCTTAAT

wp-2c AAGAACTTGA TCAGAAGGCA AAACTTGAGG CCAAGCCTTT GAAGGAGATT CTTGCTTAAT

wp-13c AAGAACTTGA TCAGAAGGCA AAACTTGAGG CCAAGCCTTT GAAGGAGATT CTTGCTTAAT

wp-12c AAGAACTTGA TCAGAAGGCA AAACTTGAGG CCAAGCCTTT GAAGGAGATT CTTGCTTAAT

wp-6c AAGAACTTGA TCAGAAGGCA AAACTTGAGG CCAAGCCTTT GAAGGAGATT CTTGCTTAAT

Consensus AAGAACTTGA TCAGAAGGCA AAACTTGAGG CCAaGCCTTT GAAGGAGATT CTTGCTTAAT

2881 2940

wp-9c TAATAATAAT TACATATGTA TCATTTGCAT GCCCCCTTGG TGTTTTTAGT ATTTTTTAAG

wp-8c TAATAATAAT TACATATGTA TCATTTGCAT GCCCCCTTGG TGTTTTTAGT ATTTTTTAAG

wp-2c TAATAATAAT TACATATGTA TCATTTGCAT GCCCCCTTGG TGTTTTTAGT ATTTTTTAAG

wp-13c TAATAATAAT TACATATGTA TCATTTGCAT GCCCCCTTGG TGTTTTTAGT ATTTTTTAAG

wp-12c TAATAATAAT TACATATGTA TCATTTGCAT GCCCCCTTGG TGTTTTTAGT ATTTTTTAAG

wp-6c TAATAATAAT TACATATGTA TCATTTGCAT GCCCCCTTGG TGTTTTTAGT ATTTTTTAAG

Consensus TAATAATAAT TACATATGTA TCATTTGCAT GCCCCCTTGG TGTTTTTAGT ATTTTTTAAG

2941 3000

wp-9c GGCCATGAAT TAATAATAGT CCTTACCTTT GTGCTTTTGT ACGTCTTATG ATTTATCCTT

wp-8c GGCCATGAAT TAATAATAGT CCTTACCTTT GTGCTTTTGT ACGTCTTATG ATTTATCCTT

wp-2c GGCCATGAAT TAATAATAGT CCTTACCTTT GTGCTTTTGT ACGTCTTATG ATTTATCCTT

wp-13c GGCCATGAAT TAATAATAGT CCTTACCTTT GTGCTTTTGT ACGTCTTATG ATTTATCCTT

wp-12c GGCCATGAAT TAATAATAGT CCTTACCTTT GTGCTTTTGT ACGTCTTATG ATTTATCCTT

wp-6c GGCCATGAAT TAATAATAGT CCTTACCTTT GTGCTTTTGT ACGTCTTATA ATTTATCCTT

Consensus GGCCATGAAT TAATAATAGT CCTTACCTTT GTGCTTTTGT ACGTCTTATg ATTTATCCTT

3001 3060

wp-9c TGTGGGGATA TCATGTGTTG TGTTCAGTTG CCTATGTCTT ATTAGCTAGC TGGCTCATCT

wp-8c TGTGGGGATA TCATGTGTTG TGTTCAGTTG CCTATGTCTT ATTAGCTAGC TGGCTCATCT

wp-2c TGTGGGGATA TCATGTGTTG TGTTCAGTTG CCTATGTCTT ATTAGCTAGC TGGCTCATCT

wp-13c TGTGGGGATA TCATGTGTTG TGTTCAGTTG CCTATGTCTT ATTAGCTAGC TGGCTCATCT

wp-12c TGTGGGGATA TCATGTGTTG TGTTCAGTTG CCTATGTCTT ATTAGCTAGC TGGCTCATCT

wp-6c TGTGGGGATA TCATGTGTTG TGTTCAGTTG CCTATGTCTT ATTAGCTAGC TGGCTCATCT

Consensus TGTGGGGATA TCATGTGTTG TGTTCAGTTG CCTATGTCTT ATTAGCTAGC TGGCTCATCT

3061 3114

wp-9c ATGTATACCT TATATTTGCC TCTATTATAA ATGAAAATAA GTGGCACTGT CTTT

wp-8c ATGTATACCT TATATTTGCC TCTATTATAA ATGAAAATAA GTGGCACTGT CTTT

wp-2c ATGTATACCT TATATTTGCC TCTATTATAA ATGAAAATAA GTGGCACTGT CTTT

wp-13c ATGTATACCT TATATTTGCC TCTATTATAA ATGAAAATAA GTGGCACTGT CTTT

wp-12c ATGTATACCT TATATTTGCC TCTATTATAA ATGAAAATAA GTGGCACTGT CTTT

wp-6c ATGTATACCT TATATTTGCC TCTATTATAA ATGAAAATAA GTGGCACTGT CTTT

Consensus ATGTATACCT TATATTTGCC TCTATTATAA ATGAAAATAA GTGGCACTGT CTTT
