# Supplementary material for: Novel exon combinations generated by alternative splicing of gene fragments mobilized by a CACTA transposon in Glycine max
Source: BMC Plant Biol. 2007 Jul 14;7:38. doi: 10.1186/1471-2229-7-38 (PMC1947982; doi:10.1186/1471-2229-7-38)
Supplement: Additional file 3 — See coat wp RT-PCR cDNA derived anubi acxud sequences and open reading frames. [file 1471-2229-7-38-S3.doc]

Seed Coat *wp* – cDNA clones derived amino acid sequences.

***wp*-25s**

>/tmp/outseq.input.22458 [Unknown form], frame+3, 1035 bases, 19D6 checksum.

IAFCYLIPLRTRTFSSKTTMAPTAKTLTYLAQEKTLESSFVRDEEERPKV

AYNEFSDEIPVISLAGIDEVDGRRREICEKIVEACENWGIFQVVDHGVDQ

QLVAEMTRLAKEFFALPPDEKLRFDMSGAKKGGFIVSSHLQGESVQDWRE

IVTYFSYPKRERDYSRWPDTPEGWRSVTEEYSDKVMGLACKLMEVLSEAM

GLEKEGLSKACVDMDQKVVVNYYPKCPQPDLTLGLKRHTDPGTITLLLQD

QVGGLQATRDNGKTWITVQPVEAAFVVNLGDHAHVDHDGIFVIRRLYSKT

SVIRKRRC*FRKKKLYAF*TLSKVTTSKLSSFYCAASTVIRRQ*L*PKIT

SEET*FRIHPQSHRVRRQRGFLRAHQRRRRRRCNCRYPK*FHRVSSKFKN

GIFKICEAHCNS*LPRLSSSSSVIYFSSFLSDDCWNSKSFFDGTVRKG*E

DRVATASPTRPSR*RRFASSRRMRGFPAPPFTRFLS*KKCSTGTLLGCRM

*CTMRRVCIWYNWFLREDKGS*IINSRTLLQG*NGVIVLTSLSLNSCFIT

LFSNVMWH*NHFQYLAEYLEHYAICYNSEAGELYSKKLAKFVGKRLKSEW

AASIWTSTLQRTILTATPIIGFPKIQWRALDEINAGVCDGMAYAEIKNMP

EEYE*VCRLVIFPLFASCMQLIWQIKRSTM*NFSLINFYDT*IKI*YVLD

KY*LRPCNSRLYDQEPYNISFGFIP*TFLKIVFSS*IFFIYF*FFNYILS

WLFCFSCKFKHVQLSFIHLMSFYFLIVSYYSITKLPIAIPGI*ERKSLWN

S*RYCFQYAMPIKGDHQI*GIGNGLIPSVLDVNLLDEVILLDEVIQYLSN

GRFKNADHQAVVNSNHSRLSIATFQNPAPNATVYPLKIREGEKPVMEEPI

TFAEMYRRKMSKDIEIARMKKLAKEKHLQDLENEKHLQELDQKAKLEAKP

LKEILA*LIIITYVSFACPLGVFSIF*GP*INNSPYLCAFVRLMIYPLWG

YHVLCSVAYVLLASWLIYVYLIFASIINENKWHCL

**(425 bp Exon 1, 429 bp Exon 2, & 74 bp UP Intron)**

>/tmp/outseq.input.22458 [Unknown form], frame+1, 1036 bases, 1CE6 checksum.

ALHSAI*FHYVHAHSPQRQQWHQQPRL*LTWPRRKP*NRASFGTRRSVPR

LPTTNSATRSQ*FLLPESTRWMDAEERFVRRSWRLARIGVYSRLLITVWI

NNSWPR*PVSPKSSLLCHRTRSFVLICPAPKRVDSLSPAISKGNRCRTGE

K**HTFRTQKERGTIQGGQTRQKGGDR*LRNTATK*WV*LASSWRCCPKQ

WG*RKRV*AKHVLTWTRRWWLITTPNALNLTSLLA*SATRIRALSPCCFR

TKWVDFKPPGTMAKHGSPFSLWRLPSSSILEIMLM*TTMAFL*FEDFILR

RVSLENVVVSLEKKSYTRSEP*ARSQPRS*VLSTVLHQQLYDDNDCDLKS

PLKKPDSASTPSPTACGVKEDFSVLTNDEEEEDVIAGIRNDFTELVASSR

TGSSKSARLTVTPNFPDCHLPLQSSTFPLFSPTIVGTQRASLMEQYEKVE

KIGSRPRHQRDHRVEEDSPRAGG*GGSQHRHSRDFSLERNAAQEHC*VVG

CSAR*EEFVSGTIGF*GKTKVVR**IQGLCCKVEMV**F*LLYP*ILASL

LSFLM*CGIRIIFNIWLNT*NTMQFVITVRLENFIQRSLPSLLESVSNQN

GLLLYGLVHCNEQF*QPLQLLDFPRYNGVHLMR*TQGCVMVWHMQKSKTC

QRSMSRFADL*FFPYLQVACN*YGR*KEAQCETSLLLIFMIHRLKYNMS*

TNIN*DLVTLDYMTRNHIIFPLDLFLKLF*KLFLVREFFLFIFSSLIIFC

LGFFVFLANLSMSSFHLYI*CLFIFLL*VTIQSQSCQLQYQVYRNGNPYG

IAEGIVFSMPCRSKVITKSKELVMVLFQVYWMLIY*MKLFY*MKLFSI*A

MEGSRMLITKRW*TQTIAVCP*PLFKTQHQMQLFTL*R*EKERSL*WRNQ

SLLLKCTGGR*ARTLRLQG*RSWLRKSICRTLRMKSICKNLIRRQNLRPS

L*RRSLLN***LHMYHLHAPLVFLVFFKGHELIIVLTFVLLYVL*FILCG

DIMCCVQLPMSY*LAGSSMYTLYLPLL*MKISGTVF

**(28 bp UP Intron, 210 bp UP, & 192 bp CDC2)**

>/tmp/outseq.input.22458 [Unknown form], frame-2, 1035 bases, 180D checksum.

KTVPLIFIYNRGKYKVYIDEPAS**DIGN*TQHMISPQRINHKTYKSTKV

RTIINSWPLKNTKNTKGACK*YICNYY*LSKDLLQRLGLKFCLLIKFLQM

LFILKVLQMLFLSQLLHPCNLNVLAHLPPVHFSKSDWFLHHRLLSFSYLQ

RVNSCIWCWVLKSGYGQTAMV*VHHRLVISILEPSIAQILNNFI**NNFI

**INIQYTWNKTITNSLDLVITFDRHGILKTIPSAIP*GFPFLYTWYCNW

QLCD*IVTHNKKIKRHQMYK*KLDMLKFARKTKKPRQNIIKELKINKKNS

RTKNNF*KSLRNKSKGNIIWFLVI*SRVTRS*LIFV*DILYFNLCIIKIN

KREVSHCASFYLPY*LHATCK*GKNYKSANLLILLWHVFDFCICHTITHP

CVYLIKCTPLYLGKSNNWSGCQNCSLQCTSPYRSSPF*FETLSNKLGKLL

*IKFSSLTVITNCIVF*VFSQILKMILMPHHIRKESNEARIQG*RS*NYY

TISTLQQSP*IYYLTTFVFPQKPIVPDTNSSHRALHPTT*QCSCAAFLSR

EKSREWRCWEPPHPPARGESSSTRWSRW*RGRDPIFSTFSYCSIKEAL*V

PTIVGEKRGKVDD*RGR*QSGKLGVTVSLADFEDPVLELATNSVKSFRIP

AITSSSSSSLVSTEKSSLTPHAVGLGVDAESGFFRGDFRSQSLSSYNC*C

STVERTQLRGCDLA*GSERV*LFFSKLTTTFSNDTRLRIKSSNYKNAIVV

YMSMISKIDDEGSLHRLNGDPCFAIVPGGLKSTHLVLKQQGDSARIRVAL

QAKSEVRLRAFGVVINHHLLVHVNTCFA*TLFL*PHCFGQHLHELAS*TH

YFVAVFLSHRSPPFWRVWPP*IVPLSFWVRKVCHYFSPVLHRFPLEMAGD

NESTLFGAGHIKTKLLVRWQSKELFGETGHLGHELLIHTVINNLEYTPIL

ASLHDLLTNLSSASIHLVDSGKRNHWDLVAEFVVGNLGTLLLVPNEARF*

GFLLGQVSQSLGCWCHCCL*GECACT*WN*IAECN

**(91 bp UP Intron & 230 bp Exon 2)**

**(70 bp Exon 2 & 314 Exon 1)**

>/tmp/outseq.input.22458 [Unknown form], frame-3, 1035 bases, 89E checksum.

RQCHLFSFIIEANIRYT*MSQLANKT*ATEHNT*YPHKG*IIRRTKAQR*

GLLLIHGP*KILKTPRGHANDTYVIIIN*ARISFKGLASSFAF*SSSCKC

FSFSRSCKCFSLASFFILAISMSLLIFLLYISAKVIGSSITGFSPSLIFR

G*TVAFGAGF*KVAMDKRLWFEFTTAW*SAFLNLPLLRY*ITSSSRITSS

SRLTSSTLGIRPLPIP*IW*SPLIGMAY*KQYLQLFHKDFRSYIPGIAIG

NFVIE**LTIRK*KDIKCINESWTCLNLQEKQKSQDKI*LKN*K*IKKIH

ELKTIFKKV*GINPKEILYGSWSYNLELQGLS*YLSKTYYILIYVS*KLI

REKFHIVLLFICHISCMQLANKGKITSLQTYSYSSGMFLISAYAIPSHTP

AFISSSARHCILGNPIIGVAVRIVRCSVLVHIEAAHSDLRRFPTNLASFF

E*SSPASLL*QIA*CSRYSAKY*K*F*CHITLEKRVMKQEFKDREVRTIT

PFQPCNKVLEFII*LPLSSLRNQLYQIQTLLIVHYILQPNNVPVLHFFQE

RNLVNGGAGNPLILLLEANLLQRDGLVGDAVATLSSQPFRTVPSKKLFEF

QQSSERKEEK*MTEEEDDNLGS*ELQ*ASQILKIPFLNLLLTL*NHFGYR

QLHLLLLRRW*ARRNPL*RRTRWDWGWMRNQVSSEVILGHSHCRRITVDA

AQ*KELSFEVVTLLRVQNAYNFFFLN*QRRFLMTLVLE*SLRITKMPSWS

T*A*SPRLTTKAASTG*TVIHVLPLSLVA*SPPTWS*SSKVIVPGSVWRF

RPRVRSG*GHLG**LTTTFWSMSTHALLKPSFSNPIASDNTSMSLQARPI

TLSLYSSVTDLHPSGVSGHLE*SLSLFGYEKYVTISLQSCTDSPWRWLET

MNPPFLAPDISKRSFSSGGKAKNSLARRVISATSC*STP*STTWNIPQFS

QASTIFSQISLLRPSTSSIPAREITGISSLNSL*ATLGRSSSSRTKLDSR

VFSWAR*VRVLAVGAIVVFEENVRVRSGIK*QNAM

**(106 bp FPK-MDH Intron & 206 bp FPK)**

***wp*-22s**

>/tmp/outseq.input.17106 [Unknown form], frame+3, 992 bases, 1F9A checksum.

IAFCYLIPLRTRTFSSKTTMAPTAKTLTYLAQEKTLESSFVRDEEERPKV

AYNEFSDEIPVISLAGIDEVDGRRREICEKIVEACENWGIFQVVDHGVDQ

QLVAEMTRLAKEFFALPPDEKLRFDMSGAKKGGFIVSSHLQGESVQDWRE

IVTYFSYPKRERDYSRWPDTPEGWRSVTEEYSDKVMGPSCKLMEVLSEAM

GLEKEGLSKACVDMDQKVVVNYYPKCPQPDLTLGLKRHTDPGTITLLLQD

QVGGLQATRDNGKTWITVQPVEAAFVVNLGDHAHTTMAFL*FEDFILRRV

SLENVVVSLEKKKLYAF*TLSKVTTSKLSSFYCAASTVIRRQ*L*PKITS

EETRFRIHPQSHRVRRQRGFLRAHQRRRRRRCNCRYPK*FHRVSSKFKNG

IFKICEAHCNS*LPRLSSSSSVIYFSSFLSDDCWNSKSFFDGTVRKG*ED

RVATASPTRPSR*RRFASSRRMRGFPAPPFTRFHS*KKCSTGTLLGCRM*

CTMRRVCIWYNWFLREDKGS*IINSRTLLQG*NG*GWRTLFKEACQVCWK

ASQIRMGCFYMD*YTATNNSDSHSNYWISQDTMACT**DKRRGV*WYGIC

RNQEKHARGV*VGLQTCNFSLICKLRATNMADKKKHNVKLLSY*FL*YID

*NIICLRQILTKTL*L*II*PGTI*YFFGFIP*TFLKIVFSS*IFFIYF*

FFNYILSWLFCFSCKFKHVQLSFIHLMSFYFLIVSYYSITKLPIAIPGI*

ERKSLWNS*RYCFQYAMPIKGDHQI*GIGNGLIPSVLDVNLLDEVILLDE

VIQYLSNGRFKNADHQAVVNSNHSRLSIATFQNPAPNATVYPLKIREGEK

PVMEEPITFAEMYRRKMSKDIEIARMKKLAKEKHLQDLENEKHLQELDQK

AKLEAKPLKEILA*LIIITYVSFACPLGVFSIF*GP*INNSPYLCAFVRL

MIYPLWGYHVLCSVAYVLLASWLIYVYLIFASIINENKWHCL

**(425 bp Exon 1, 429 bp Exon 2, & 18 bp UP Intron)**

>/tmp/outseq.input.17106 [Unknown form], frame+1, 993 bases, 10A9 checksum.

ALHSAI*FHYVHAHSPQRQQWHQQPRL*LTWPRRKP*NRASFGTRRSVPR

LPTTNSATRSQ*FLLPESTRWMDAEERFVRRSWRLARIGVYSRLLITVWI

NNSWPR*PVSPKSSLLCHRTRSFVLICPAPKRVDSLSPAISKGNRCRTGE

K**HTFRTQKERGTIQGGQTRQKGGDR*LRNTATK*WVHLASSWRCCPKQ

WG*RKRV*AKHVLTWTRRWWLITTPNALNLTSLLA*SATRIRALSPCCFR

TKWVDFKPPGTMAKHGSPFSLWRLPSSSILEIMLIPRWHFCNSKTLF*DE

CH*KTSLLV*KKKSYTRSEP*ARSQPRS*VLSTVLHQQLYDDNDCDLKSP

LKKPDSASTPSPTACGVKEDFSVLTNDEEEEDVIAGIRNDFTELVASSRT

GSSKSARLTVTPNFPDCHLPLQSSTFPLFSPMIVGTQRASLMEQYEKVEK

IGSRPRHQRDHRVEEDSPRAGG*GGSQHRHSRDFTLERNAAQEHC*VVGC

SAR*EEFVSGTIGF*GKTKVVR**IQGLCCKVEMVEAGELYSKKLAKFVG

KRLKSEWAASIWTSTLQRTILTATPIIGFPKIQWRALDEINAGVCDGMAY

AEIKKNMPEEYE*VCRLVIFPLFASCVQLIWQIKRSTM*NFSLINFYDT*

IKI*YVLDKY*LRPCNSRLYDQEPYNISLDLFLKLF*KLFLVREFFLFIF

SSLIIFCLGFFVFLANLSMSSFHLYI*CLFIFLL*VTIQSQSCQLQYQVY

RNGNPYGIAEGIVFSMPCRSKVITKSKELVMVSFQVYWMLIY*MKLFYWM

KLFSI*AMEGSRMLITKRW*TQTIAVCP*PLFKTQHQMQLFTL*R*EKER

SL*WRNQSLLLKCTGGR*ARTLRLQG*RSWLRKSICRTLRMKSICKNLIR

RQNLRPSL*RRFLLN***LHMYHLHAPLVFLVFFKGHELIIVLTFVLLYV

L*FILCGDIMCCVQLPMSY*LAGSSMYTLYLPLL*MKISGTVF

**(28 bp UP Intron, 210 bp UP, & 192 bp CDC2)**

>/tmp/outseq.input.17106 [Unknown form], frame-2, 992 bases, 23D6 checksum.

KTVPLIFIYNRGKYKVYIDEPAS**DIGN*TQHMISPQRINHKTYKSTKV

RTIINSWPLKNTKNTKGACK*YICNYY*LSKNLLQRLGLKFCLLIKFLQM

LFILKVLQMLFLSQLLHPCNLNVLAHLPPVHFSKSDWFLHHRLLSFSYLQ

RVNSCIWCWVLKSGYGQTAMV*VHHRLVISILEPSIAQILNNFIQ*NNFI

**INIQYTWNETITNSLDLVITFDRHGILKTIPSAIP*GFPFLYTWYCNW

QLCD*IVTHNKKIKRHQMYK*KLDMLKFARKTKKPRQNIIKELKINKKNS

RTKNNF*KSLRNKSKEILYGSWSYNLELQGLS*YLSKTYYILIYVS*KLI

REKFHIVLLFICHISCTQLANKGKITSLQTYSYSSGMFFLISAYAIPSHT

PAFISSSARHCILGNPIIGVAVRIVRCSVLVHIEAAHSDLRRFPTNLASF

FE*SSPASTISTLQQSP*IYYLTTFVFPQKPIVPDTNSSHRALHPTT*QC

SCAAFLSRVKSREWRCWEPPHPPARGESSSTRWSRW*RGRDPIFSTFSYC

SIKEAL*VPTIIGEKRGKVDD*RGR*QSGKLGVTVSLADFEDPVLELATN

SVKSFRIPAITSSSSSSLVSTEKSSLTPHAVGLGVDAESGFFRGDFRSQS

LSSYNC*CSTVERTQLRGCDLA*GSERV*LFFF*TNNDVF**HSS*NKVF

ELQKCHRGMSMISKIDDEGSLHRLNGDPCFAIVPGGLKSTHLVLKQQGDS

ARIRVALQAKSEVRLRAFGVVINHHLLVHVNTCFA*TLFL*PHCFGQHLH

ELARWTHYFVAVFLSHRSPPFWRVWPP*IVPLSFWVRKVCHYFSPVLHRF

PLEMAGDNESTLFGAGHIKTKLLVRWQSKELFGETGHLGHELLIHTVINN

LEYTPILASLHDLLTNLSSASIHLVDSGKRNHWDLVAEFVVGNLGTLLLV

PNEARF*GFLLGQVSQSLGCWCHCCL*GECACT*WN*IAECN

**(103 bp FPK-MDH Intron & 212 bp FPK)**

***wp*-28s**

>/tmp/outseq.input.18124 [Unknown form], frame+3, 944 bases, 395 checksum.

IAFCYLIPLRTHTFSSKTTMAPTAKTLTYLAQEKTLESSFVRDEEESPKV

AYNEFSDEIPVISLAGIDEVDGRRREICEKIVEACENWGIFQVVDHGVDQ

QLVAEMTRLAKEFFALPPDEKLRFDMSGAKKGGFIVSSHLQGESVQDWRE

IVTYFSYPKRERDYSRWPDTPEGWRSVTEEYSDKVMGLACKLMEVLSEAM

GLEKEGLSKACVDMDQKVVVNYYPKCPQPDLTLGLKRRTDPGTITLLLQD

QVGGLQATRDNGKTWITVQPVEAAFVVNLGDHAHLYDDNDCDLKSPLKKP

DSASTPSPTACGVKEDFSVLTNDEEEEDVIAGIRNDFTELVASSRTGSSK

SARLTVTPNFPDCHLPLQSPTFPLFSPMIVGTQRASLMEQYEKVEKIGSR

PRHQRDHRVEEDSPRAGG*GGSQHRHSRDFSLERNAAQEHC*VVGCSAR*

EEFVSGTIGF*GKTKVVR**IQGLCCKVEMVEAGELYSKKLAKFVGKRLK

SEWAASVSIIYYMD*YTATNNSDSHSNYWISQDTMACT**DKRRGV*WYG

ICRNQEKHARGV*VGLQTCNFSLICKLHATNMADKKKHNVKLLSY*FL*Y

ID*NIICLRQILTKTL*L*II*PGTI*YFFGFIP*TFLKIVFSS*IFFIY

F*FFNYILSWLFCFSCKFKHVQLSFIHLMSFYFLIVSYYSITKLPIAIPG

I*ERKSLWNS*RYCFQYAMPIKGDHQI*GIGNGLIPSVLDVNLLDEVILL

DEVIQYLSNGRFKNADH*AVVNSNHSRLSIATFQNPAPNATVYPLKIREG

EKPVMEEPITFAEMYRRKMSKDIEIARMKKLAKEKHLQDLENEKHLQELD

QKAKLEAKPLKEILA*LIIITYVSFACPLGVFSIF*GP*INNSPYLCAFV

RLMIYPLWGYHVLCSVAYVLLASWLIYVYLIFASIINENKWHCL

**(425 bp Exon 1, 429 bp Exon 2, 209 bp UP, & 192 bp CDC2)**

>/tmp/outseq.input.18124 [Unknown form], frame-2, 944 bases, 1FE4 checksum.

KTVPLIFIYNRGKYKVYIDEPAS**DIGN*TQHMISPQRINHKTYKSTKV

RTIINSWPLKNTKNTKGACK*YICNYY*LSKNLLQRLGLEFCLLIKFLQM

LFILKVLQMLFLSQLLHPCNLNVLAHLPPVHFSKSDWFLHHRLLSFSYLQ

RVNSCIWCWVLKSGYGQTAMV*VHHRLVISILEPSIAQILNNFI**NNFI

**INIQYTWNETITNSLDLVITFDRHGILKTIPSAIP*GFPFLYTWYCNW

QLCD*IVTHNKKIKRHQMYK*KLDMLKFARKTKKPRQNIIKELKINKKNS

RTKNNF*KSLRNKSKEILYGSWSYNLELQGLS*YLSKTYYILIYVS*KLI

REKFHIVLLFICHISCMQLANKRKITSLQTYSYSSGMFFLISAYAIPSHT

PAFISSSARHCILGNPIIGVAVRIVRCSVLVHIIDNAYRSSPF*FETLSN

KLGKLL*IKFSSLNHFNLATKSLNLLSNYLCLPSETNCTRYKLFSSCTTS

YNLTMFLCCISFKREIS*MAVLGTPSSSCSRRIFFNAMVSLVTRSRPYLL

NLFVLFHQRSSLSSNNHRREKRKSR*LKRKMTIWEVRSYSEPRRF*RSRS

*TCY*LCEIISDTGNYIFFFFVVGEHGEILFDAARGGTGGGCGIGFLQR*

F*VTVIVVV*MSMISKIDDEGSLHRLNGDPCFAIVPGGLKSTHLVLKQQG

DSARIRAALQAKSEVRLRAFGVVINHHLLVHVNTCFA*TLFL*PHCFGQH

LHELAS*THYFVAVFLSHRSPPFWRVWPP*IVPLSFWVRKVCHYFSPVLH

RFPLEMAGDNESTLFGAGHIKTKLLVRWQSKELFGETGHLGHELLIHTVI

NNLEYTPILASLHDLLTNLSSASIHLVDSGKRNHWDLVAEFVVGNLGTLL

LVPNEARF*GFLLGQVSQSLGCWCHCCL*GECVCT*WN*IAECN

**(70 bp Exon 2 & 314 Exon 1)**

***wp*-9s**

>/tmp/outseq.input.9985 [Unknown form], frame+3, 939 bases, 141C checksum.

IAFCYLIPLRTRTFSSKTTMAPTAKTLTYLAQEKTLESSFVRDEEERPKV

AYNEFSDEIPVISLAGIDEVDGRRREICEKIVEACENWGIFQVVDHGVDQ

QLVAEMTRLAKEFFALPPDEKLRFDMSGAKKGGFIVSSHLQGESVQDWRE

IVTYFSYPKRERDYSRWPDTPEGWRSVTEEYSDKVMGLACKLMEVLSEAM

GLEKEGLSKACVDMDQKVVVNYYPKCPQPDLTLGLKRHTDPGTITLLLQD

QVGGLQATRDNGKTWITVQPVEAAFVVNLGDHAHLYDDNDCDLKSPLKKP

DSASTPSPTACGVEEVFSVLTNDEEEEDVIAGIRNDFTELVASSRTGSSK

SARLTVTPNFPDCHLPLQSSTFPLFSPMIVGTQRASLMEQYEKVEKIGSR

PRHQRDHRVEEDSPRAGG*GGSQHRHSRDFSLERNAAQEHC*VVGCSAR*

EEFVSGTIGF*GKTKVVR**IQGLCCKVEMVEAGELYSKKLAKFVGKRLK

SEWAASIWTSTLQRTILTATPIIGFPKIQWRALDEINAGVCDSMAYAEIK

KNMPEEYE*VCRLVIFPLFASCMQLIWQIKRSTM*NFSLINFYDT*IKI*

YVLDKY*LRPCNSRLYDQEPYNISLDLFLKLF*KLFLVREFFLFIFSSLI

IFCLGFFVFLANLSMSSFHLYI*CLFIFLL*VTIQSQSCQLQYQVYRNGN

PYGIAEGIVFSMPCRSKVITKSKELVMVSFQVYWMLIY*MKLFY*MKLFS

I*AMEGSRMLITKRW*TQTIAVCP*PLFKTQHQMQLFTL*R*EKERSL*W

RNQSLLLKCTGGR*ARTLRLQG*RSWLRKSICRTLRMKSICKNLIRRQNL

RPSL*RRFLLN***LHMYHLHAPLVFLVFFKGHELIIVLTFVLLYVL*FI

LCGDIMCCVQLPMSY*LAGSSMYTLYLPLL*MKISGTVF

**(425 bp Exon 1, 429 bp Exon 2, 209 bp UP, & 192 bp CDC2)**

>/tmp/outseq.input.9985 [Unknown form], frame+2, 939 bases, 1532 checksum.

HCILLFNSTTYTHILLKDNNGTNSQDSDLPGPGENPRIELRSGRGGASQG

CLQRIQRRDPSDFSCRNRRGGWTQKRDL*EDRGGLRELGYIPGC*SRCGS

TTRGRDDPSRQRVLCFATGREASF*YVRRQKGWIHCLQPSPRGIGAGLER

NSDILFVPKKREGLFKVARHARRVEIGD*GIQRQSNGSSLQAHGGVVRSN

GVRERGFKQSMC*HGPEGGG*LLPQMPST*PHSWPEAPHGSGHYHLAASG

PSGWTSSHQGQWQNMDHRSACGGCLRRQSWRSCSFIRRQ*L*PKITSEET

RFRIHPQSHRVRRRRGFLRAHQRRRRRRCNCRYPK*FHRVSSKFKNGIFK

ICEAHCNS*LPRLSSSSSVIYFSSFLSDDCWNSKSFFDGTVRKG*EDRVA

TASPTRPSR*RRFASSRRMRGFPAPPFTRFLS*KKCSTGTLLGCRM*CTM

RRVCIWYNWFLREDKGS*IINSRTLLQG*NG*GWRTLFKEACQVCWKASQ

IRMGCFYMD*YTATNNSDSHSNYWISQDTMACT**DKRRGV**YGICRNQ

EKHARGV*VGLQTCNFSLICKLHATNMADKKKHNVKLLSY*FL*YID*NI

ICLRQILTKTL*L*II*PGTI*YFFGFIP*TFLKIVFSS*IFFIYF*FFN

YILSWLFCFSCKFKHVQLSFIHLMSFYFLIVSYYSITKLPIAIPGI*ERK

SLWNS*RYCFQYAMPIKGDHQI*GIGNGLIPSVLDVNLLDEVILLDEVIQ

YLSNGRFKNADHQAVVNSNHSRLSIATFQNPAPNATVYPLKIREGEKPVM

EEPITFAEMYRRKMSKDIEIARMKKLAKEKHLQDLENEKHLQELDQKAKL

EAKPLKEILA*LIIITYVSFACPLGVFSIF*GP*INNSPYLCAFVRLMIY

PLWGYHVLCSVAYVLLASWLIYVYLIFASIINENKWHCL

**(81 bp CS & 329 bp Exon 3)**

>/tmp/outseq.input.9985 [Unknown form], frame-1, 939 bases, 1027 checksum.

KDSATYFHL**RQI*GIHR*AS*LIRHRQLNTTHDIPTKDKS*DVQKHKG

KDYY*FMALKKY*KHQGGMQMIHM*LLLIKQESPSKAWPQVLPSDQVLAN

AFHSQGPANAFP*PASSSLQSQCPCSSSSCTFQQK*LVPPSQASLLLLSS

EGKQLHLVLGFEKWLWTNGYGLSSPPLGDQHS*TFHCSDTE*LHLVE*LH

LVD*HPVHLE*DHYQFLRFGDHL*SAWHTENNTFSYSIRISVPIYLVLQL

ATL*LNSNSQ*ENKKTSNV*MKAGHA*ICKKNKKAKTKYN*RTKNK*KKF

TN*KQFLKKFKE*IQRNIIWFLVI*SRVTRS*LIFV*DILYFNLCIIKIN

KREVSHCASFYLPY*LHATCK*GKNYKSANLLILLWHVFLDFCICHTITH

PCVYLIKCTPLYLGKSNNWSGCQNCSLQCTSPYRSSPF*FETLSNKLGKL

L*IKFSSLNHFNLATKSLNLLSNYLCLPSETNCTRYKLFSSCTTSYNLTM

FLCCISFKREIS*MAVLGTPSSSCSRRIFFNAMVSLVTRSRPYLLNLFVL

FHQRSSLSSNNHRREKRKSR*LKRKMTIWEVRSYSEPRRF*RSRS*TCY*

LCEIISDTGNYIFFFFVVGEHGENLFDAARGGTGGGCGIGFLQR*F*VTV

IVVV*MSMISKIDDEGSLHRLNGDPCFAIVPGGLKSTHLVLKQQGDSARI

RVALQAKSEVRLRAFGVVINHHLLVHVNTCFA*TLFL*PHCFGQHLHELA

S*THYFVAVFLSHRSPPFWRVWPP*IVPLSFWVRKVCHYFSPVLHRFPLE

MAGDNESTLFGAGHIKTKLLVRWQSKELFGETGHLGHELLIHTVINNLEY

TPILASLHDLLTNLSSASIHLVDSGKRNHWDLVAEFVVGNLGTLLLVPNE

ARF*GFLLGQVSQSLGCWCHCCL*GECACT*WN*IAECN

**(70 bp Exon 2 & 314 Exon 1)**

>/tmp/outseq.input.9985 [Unknown form], frame-2, 939 bases, E71 checksum.

KTVPLIFIYNRGKYKVYIDEPAS**DIGN*TQHMISPQRINHKTYKSTKV

RTIINSWPLKNTKNTKGACK*YICNYY*LSKNLLQRLGLKFCLLIKFLQM

LFILKVLQMLFLSQLLHPCNLNVLAHLPPVHFSKSDWFLHHRLLSFSYLQ

RVNSCIWCWVLKSGYGQTAMV*VHHRLVISILEPSIAQILNNFI**NNFI

**INIQYTWNETITNSLDLVITFDRHGILKTIPSAIP*GFPFLYTWYCNW

QLCD*IVTHNKKIKRHQMYK*KLDMLKFARKTKKPRQNIIKELKINKKNS

RTKNNF*KSLRNKSKEILYGSWSYNLELQGLS*YLSKTYYILIYVS*KLI

REKFHIVLLFICHISCMQLANKGKITSLQTYSYSSGMFFLISAYAILSHT

PAFISSSARHCILGNPIIGVAVRIVRCSVLVHIEAAHSDLRRFPTNLASF

FE*SSPASTISTLQQSP*IYYLTTFVFPQKPIVPDTNSSHRALHPTT*QC

SCAAFLSREKSREWRCWEPPHPPARGESSSTRWSRW*RGRDPIFSTFSYC

SIKEAL*VPTIIGEKRGKVDD*RGR*QSGKLGVTVSLADFEDPVLELATN

SVKSFRIPAITSSSSSSLVSTEKTSSTPHAVGLGVDAESGFFRGDFRSQS

LSSYK*A*SPRLTTKAASTG*TVIHVLPLSLVA*SPPTWS*SSKVIVPGS

VWRFRPRVRSG*GHLG**LTTTFWSMSTHALLKPSFSNPIASDNTSMSLQ

ARPITLSLYSSVTDLHPSGVSGHLE*SLSLFGYEKYVTISLQSCTDSPWR

WLETMNPPFLAPDISKRSFSSGGKAKNSLARRVISATSC*STP*STTWNI

PQFSQASTIFSQISLLRPSTSSIPAREITGISSLNSL*ATLGRSSSSRTK

LDSRVFSWAR*VRVLAVGAIVVFEENVRVRSGIK*QNAM

**(103 bp FPK-MDH Intron, & 212 bp FPK)**

***wp*-12s**

>/tmp/outseq.input.14542 [Unknown form], frame+3, 895 bases, 22E checksum.

IAFCYLIPLRTRTFSSKTTMAPTAKTLTYLAQEKTLESSFVRDEEERPKV

AYNEFSDEIPVISLAGIDEVDGRRREICEKIVEACENWGIFQVVDHGVDQ

QLVAEMTRLAKEFFALPPDEKLRFDMSGAKKGGFIVSSHLQDWREIVTYF

SYPKRERDYSRWPDTPEGWRSVTEEYSDKVMGLACKLMEVLSEAMGLEKE

GLSKACVDMDQKVVVNYYPKCPQPDLTLGLKRHTDPGTITLLLQDQVGGL

QATRDNGKTWITVQPVEAAFVVNLGDHAHVDHDGIFVIRRLYSKTSVIRK

RRC*FRKKKVIRVLNPKQGHNLEAEFFLLCCINSYTTTMTVT*NHL*RNP

IPHPPPVPPRAASKRISPCSPTTKKKKM*LPVSEMISQS**QVQERDLQN

LRGSL*LLTSQIVIFLFSHLLFLFSLR*LLELKELL*WNSTKRLRR*GRD

RVTNETIALKKIRLEQEDEGVPSTAIHEISLLKEMQHRNIVRL*DVVHDE

KSLYLVQLVSEGRQR*LDNKFKDFVARLKWCNSSNFSILEFLLHYSLF*C

DMWH*NHFQYLAEYLEHYAICYNSEAGELYSKKLAKFVGKRLKSEWAASI

WTSTLQRTILTATPIIGFPKIQWRALDEINAGVCDGMAYAEIKKSMPEEY

EYIGTEILME*LKVLFSVCHADQR*SPNLRNW*WSHSKCTGC*STR*SYS

TR*SYSVSEQWKVQEC*SPSGGELKP*PFVHSHFSKPSTKCNCLPSEDKR

RREACDGGTNHFC*NVQEEDEQGH*DCKDEEAG*GKAFAGP*E*KAFART

*SEGKT*GQAFEGDSCLINNNYICIICMPPWCF*YFLRAMN***SLPLCF

CTSYDLSFVGISCVVFSCLCLIS*LAHLCIPYICLYYK*K*VALS

**(425 bp Exon 1, 414 bp Exon 2, & 74 bp UP Intron)**

**(57 bp FPK Intron, 231 bp FPK & 27 bp MDH)**

>/tmp/outseq.input.14542 [Unknown form], frame+2, 896 bases, 304 checksum.

HCILLFNSTTYTHILLKDNNGTNSQDSDLPGPGENPRIELRSGRGGASQG

CLQRIQRRDPSDFSCRNRRGGWTQKRDL*EDRGGLRELGYIPGC*SRCGS

TTRGRDDPSRQRVLCFATGREASF*YVRRQKGWIHCLQPSPRLERNSDIL

FVPKKREGLFKVARHARRVEIGD*GIQRQSNGSSLQAHGGVVRSNGVRER

GFKQSMC*HGPEGGG*LLPQMPST*PHSWPEAPHGSGHYHLAASGPSGWT

SSHQGQWQNMDHRSACGGCLRRQSWRSCSCRPRWHFCNSKTLF*DECH*K

TSLLV*KKKSYTRSEP*ARSQPRS*VLSTVLHQQLYDDNDCDLKSPLKKP

DSASTPSPTACGVKEDFSVLTNDEEEEDVIAGIRNDFTELVASSRTGSSK

SARLTVTPNFPDCHLPLQSSTFPLFSPMIVGTQRASLMEQYEEVEKIGSR

PRHQRDHRVEEDSPRAGG*GGSQHRHSRDFSLERNAAQEHC*VVGCSAR*

EEFVSGTIGF*GKTKVVR**IQGLCCKVEMV**F*LLYP*ILASLLSFLM

*YVALESFSIFG*IPRTLCNLL*Q*GWRTLFKEACQVCWKASQIRMGCFY

MD*YTATNNSDSHSNYWISQDTMACT**DKRRGV*WYGICRNQEKHARGV

*VYRNGNPYGIAEGIVFSMPCRSKVITKSKELVMVSFQVYWMLIY*MKLF

Y*MKLFSI*AMEGSRMLITKRW*TQTIAVCP*PLFKTQHQMQLFTL*R*E

KERSL*WRNQSLLLKCTGGR*ARTLRLQG*RSWLRKSICRTLRMKSICKN

LIRRQNLRPSL*RRFLLN***LHMYHLHAPLVFLVFFKGHELIIVLTFVL

LYVL*FILCGDIMCCVQLPMSY*LAGSSMYTLYLPLL*MKISGTVF

**(28 bp UP Intron, 210 bp UP, & 192 bp CDC2)**

>/tmp/outseq.input.14542 [Unknown form], frame+1, 896 bases, 2395 checksum.

ALHSAI*FHYVHAHSPQRQQWHQQPRL*LTWPRRKP*NRASFGTRRSVPR

LPTTNSATRSQ*FLLPESTRWMDAEERFVRRSWRLARIGVYSRLLITVWI

NNSWPR*PVSPKSSLLCHRTRSFVLICPAPKRVDSLSPAISKTGEK**HT

FRTQKERGTIQGGQTRQKGGDR*LRNTATK*WV*LASSWRCCPKQWG*RK

RV*AKHVLTWTRRWWLITTPNALNLTSLLA*SATRIRALSPCCFRTKWVD

FKPPGTMAKHGSPFSLWRLPSSSILEIMLM*TTMAFL*FEDFILRRVSLE

NVVVSLEKKKLYAF*TLSKVTTSKLSSFYCAASTVIRRQ*L*PKITSEET

RFRIHPQSHRVRRQRGFLRAHQRRRRRRCNCRYPK*FHRVSSKFKNGIFK

ICEAHCNS*LPRLSSSSSVIYFSSFLSDDCWNSKSFFDGTVRRG*EDRVA

TASPTRPSR*RRFASSRRMRGFPAPPFTRFLS*KKCSTGTLLGCRM*CTM

RRVCIWYNWFLREDKGS*IINSRTLLQG*NGVIVLTSLSLNSCFITLFSN

VICGIRIIFNIWLNT*NTMQFVITVRLENFIQRSLPSLLESVSNQNGLLL

YGLVHCNEQF*QPLQLLDFPRYNGVHLMR*TQGCVMVWHMQKSRKACQRS

MSI*ERKSLWNS*RYCFQYAMPIKGDHQI*GIGNGLIPSVLDVNLLDEVI

LLDEVIQYLSNGRFKNADHQAVVNSNHSRLSIATFQNPAPNATVYPLKIR

EGEKPVMEEPITFAEMYRRKMSKDIEIARMKKLAKEKHLQDLENEKHLQE

LDQKAKLEAKPLKEILA*LIIITYVSFACPLGVFSIF*GP*INNSPYLCA

FVRLMIYPLWGYHVLCSVAYVLLASWLIYVYLIFASIINENKWHCL

**(81 bp CS & 329 bp Exon 3)**

>/tmp/outseq.input.14542 [Unknown form], frame-3, 895 bases, 178F checksum.

RQCHLFSFIIEANIRYT*MSQLANKT*ATEHNT*YPHKG*IIRRTKAQR*

GLLLIHGP*KILKTPRGHANDTYVIIIN*ARISFKGLASSFAF*SSSCKC

FSFSRSCKCFSLASFFILAISMSLLIFLLYISAKVIGSSITGFSPSLIFR

G*TVAFGAGF*KVAMDKRLWFEFTTAW*SAFLNLPLLRY*ITSSSRITSS

SRLTSSTLGMRPLPIP*IW*SPLIGMAY*KQYLQLFHKDFRSYILILLWH

AFLDFCICHTITHPCVYLIKCTPLYLGKSNNWSGCQNCSLQCTSPYRSSP

F*FETLSNKLGKLL*IKFSSLTVITNCIVF*VFSQILKMILMPHITLEKR

VMKQEFKDREVRTITPFQPCNKVLEFII*LPLSSLRNQLYQIQTLLIVHY

ILQPNNVPVLHFFQERNLVNGGAGNPLILLLEANLLQRDGLVGDAVATLS

SQPLRTVPSKKLFEFQQSSERKEEK*MTEEEDDNLGS*ELQ*ASQILKIP

FLNLLLTL*NHFGYRQLHLLLLRRW*ARRNPL*RRTRWDWGWMRNRVSSE

VILGHSHCRRITVDAAQ*KELSFEVVTLLRVQNAYNFFFSKLTTTFSNDT

RLRIKSSNYKNAIVVYMSMISKIDDEGSLHRLNGDPCFAIVPGGLKSTHL

VLKQQGDSARIRVALQAKSEVRLRAFGVVINHHLLVHVNTCFA*TLFL*P

HCFGQHLHELAS*THYFVAVFLSHRSPPFWRVWPP*IVPLSFWVRKVCHY

FSPVLEMAGDNESTLFGAGHIKTKLLVRWQSKELFGETGHLGHELLIHTV

INNLEYTPILASLHDLLTNLSSASIHLVDSGKRNHWDLVAEFVVGNLGTL

LLVPNEARF*GFLLGQVSQSLGCWCHCCL*GECACT*WN*IAECN

**(145 bp UP Intron & 230 bp Exon 2)**

***wp*-4s**

>/tmp/outseq.input.16002 [Unknown form], frame+3, 748 bases, B23 checksum.

IAFCYLIPLRTRTFSSKTTMAPTAKTLTYLAQEKTLESSFVRDEEERPKV

AYNEFSDEIPVISLAGIDEVDGRRREICEKIVEACENWGIFQVVDHGVDQ

QLVAEMTRLAKEFFALPPDEKLRFDMSGAKRVDSLSPAISKGNRCRTGEK

**HTFRTQKERGTIQGGQTRQKGGDR*LRNTATK*WV*LASSWRCCPKQW

G*RKRV*AKHVLTWTRRWWLITTPNALNLTSLLA*SATRIRALSPCCFRT

KWVDFKPPGTMAKHGSPFSLWRLPSSSILEIMLIYTTTMTVT*NHL*RNP

IPHPPPVPPRAASKRISPCSPTTKKKKM*LPVSEMISQS**QVQERDLQN

LRGSL*LLTSQIVIFLFSHLLFLFSLR*LLELKELL*WNSTKRLRR*GRD

RVTNETIALKKIRLEQEDEGVPSTAIHEISLLKEMQHRNIVRL*DVVHDE

KSLYLVQLVSEGRQR*LDNKFKDFVARLKWLRLENFIQRSLPSLLESVSN

QNGLLLYGLVHCNEQF*QPLQLLDFPRYNGVHLMR*TQGCVMVWHMQKSR

KTCQRSMSSI*AMEGSRMLITKRW*TQTIAVCP*PLFKTQHQMQLFTL*R

*EKERSL*WRNQSLLLKCTGGR*ARTLRLQG*RSWLRKSICRTLRMKSIC

KNLIRRQNLRPSL*RRFLLN***LHMYHLHAPLVFLVFFKGHELIIVLTF

VLLYVL*FILCGDIMCCVQLPMSY*LAGSSMYTLYLPLL*MKISGTVF

**(424 bp Exon 1 & 27 bp Exon 2)**

>/tmp/outseq.input.16002 [Unknown form], frame+2, 748 bases, 845 checksum.

HCILLFNSTTYTHILLKDNNGTNSQDSDLPGPGENPRIELRSGRGGASQG

CLQRIQRRDPSDFSCRNRRGGWTQKRDL*EDRGGLRELGYIPGC*SRCGS

TTRGRDDPSRQRVLCFATGREASF*YVRRQKGGFIVSSHLQGESVQDWRE

IVTYFSYPKRERDYSRWPDTPEGWRSVTEEYSDKVMGLACKLMEVLSEAM

GLEKEGLSKACVDMDQKVVVNYYPKCPQPDLTLGLKRHTDPGTITLLLQD

QVGGLQATRDNGKTWITVQPVEAAFVVNLGDHAHLYDDNDCDLKSPLKKP

DSASTPSPTACGVKEDFSVLTNDEEEEDVIAGIRNDFTELVASSRTGSSK

SARLTVTPNFPDCHLPLQSSTFPLFSPMIVGTQRASLMEQYEKVEKIGSR

PRHQRDHRVEEDSPRAGG*GGSQHRHSRDFSLERNAAQEHC*VVGCSAR*

EEFVSGTIGF*GKTKVVR**IQGLCCKVEMVEAGELYSKKLAKFVGKRLK

SEWAASIWTSTLQRTILTATPIIGFPKIQWRALDEINAGVCDGMAYAEIK

KNMPEEYE*YLSNGRFKNADHQAVVNSNHSRLSIATFQNPAPNATVYPLK

IREGEKPVMEEPITFAEMYRRKMSKDIEIARMKKLAKEKHLQDLENEKHL

QELDQKAKLEAKPLKEILA*LIIITYVSFACPLGVFSIF*GP*INNSPYL

CAFVRLMIYPLWGYHVLCSVAYVLLASWLIYVYLIFASIINENKWHCL

**(49 bp Exon 1, 429 bp Exon 2, 209 bp UP, & 192 bp CDC2)**

>/tmp/outseq.input.16002 [Unknown form], frame-3, 748 bases, 22F6 checksum.

RQCHLFSFIIEANIRYT*MSQLANKT*ATEHNT*YPHKG*IIRRTKAQR*

GLLLIHGP*KILKTPRGHANDTYVIIIN*ARISFKGLASSFAF*SSSCKC

FSFSRSCKCFSLASFFILAISMSLLIFLLYISAKVIGSSITGFSPSLIFR

G*TVAFGAGF*KVAMDKRLWFEFTTAW*SAFLNLPLLRYYSYSSGMFFLI

SAYAIPSHTPAFISSSARHCILGNPIIGVAVRIVRCSVLVHIEAAHSDLR

RFPTNLASFFE*SSPASTISTLQQSP*IYYLTTFVFPQKPIVPDTNSSHR

ALHPTT*QCSCAAFLSREKSREWRCWEPPHPPARGESSSTRWSRW*RGRD

PIFSTFSYCSIKEAL*VPTIIGEKRGKVDD*RGR*QSGKLGVTVSLADFE

DPVLELATNSVKSFRIPAITSSSSSSLVSTEKSSLTPHAVGLGVDAESGF

FRGDFRSQSLSSYK*A*SPRLTTKAASTG*TVIHVLPLSLVA*SPPTWS*

SSKVIVPGSVWRFRPRVRSG*GHLG**LTTTFWSMSTHALLKPSFSNPIA

SDNTSMSLQARPITLSLYSSVTDLHPSGVSGHLE*SLSLFGYEKYVTISL

QSCTDSPWRWLETMNPPFWRRTYQNEASRPVAKQRTLWRDGSSRPRVVDP

HRDQQPGIYPNSRKPPRSSHKSLFCVHPPRRFRQEKSLGSRR*IRCRQPW

DAPPRPERSSILGFSPGPGKSESWLLVPLLSLRRMCVYVVELNSRMQC

**(66 bp Exon 2 & 255 bp Exon 1)**

***wp*-15s**

>/tmp/outseq.input.16146 [Unknown form], frame+3, 473 bases, 19EF checksum.

IAFCYLIPLRTRTFSSKTTMAPTAKTLTYLAQEKTLESSFVRDEEERPKV

AYNEFSDEIPVISLAGIDEVDGRRREICEKIVEACENWGIFQVVDHGVDQ

QLVAEITRLAKEFFALPPDEKLRFDMSGAKKGGFIVSSHLQGESVQDWRE

IVTYFSYPKRERDYSRWPDTPEGWRSVTEEYSDKVMGLACKLMEVLSEAM

GLEKEGLSKACVDMDQKVVVNYYPKCPQPDLTLGLKRHTDPGTITLLLQD

QVGGLQATRDNGKTWITAQPVEAAFVVNLGDHAHYLSNGRFKNADHQAVV

NSDHSRLSIATFQNPAPNATVYPLKIREGEKPVMEEPITFAEMYRRKMSK

DIEIARMKKLAKEKHLQDLENEKHLQELDQKAKLEAKPLKEILA*LIIIT

YVSFACPLGVFSIF*GP*INNSPYLCAFVRLMIYPLWGYHVLCSVAYVLL

ASWLIYVYLIFASIINENKWHCL

**(425 bp Exon 1, 429 bp Exon 2, & 330 bp Exon 3)**

>/tmp/outseq.input.16146 [Unknown form], frame-2, 473 bases, 189A checksum.

KTVPLIFIYNRGKYKVYIDEPAS**DIGN*TQHMISPQRINHKTYKSTKV

RTIINSWPLKNTKNTKGACK*YICNYY*LSKNLLQRLGLKFCLLIKFLQM

LFILKVLQMLFLSQLLHPCNLNVLAHLPPVHFSKSDWFLHHRLLSFSYLQ

RVNSCIWCWVLKSGYGQTAMV*VHHRLVISILEPSIAQIMSMISKIDDEG

SLHRLSGDPCFAIVPGGLKSTHLVLKQQGDSARIRVALQAKSEVRLRAFG

VVINHHLLVHVNTCFA*TLFL*PHCFGQHLHELAS*THYFVAVFLSHRSP

PFWRVWPP*IVPLSFWVRKVCHYFSPVLHRFPLEMAGDNESTLFGAGHIK

TKLLVRWQSKELFGETGYLGHELLIHTVINNLEYTPILASLHDLLTNLSS

ASIHLVDSGKRNHWDLVAEFVVGNLGTLLLVPNEARF*GFLLGQVSQSLG

CWCHCCL*GECACT*WN*IAECN

**(70 bp Exon 2 & 314 Exon 1)**

** Exon 1, Exon 2, & Exon 3**

** UP Intron**

** UP**

** CDC2**

** FPK Intron**

** FPK**

** FPK-MDH Intron**

** MDH**

** CS**
