# Supplementary material for: Novel exon combinations generated by alternative splicing of gene fragments mobilized by a CACTA transposon in Glycine max
Source: BMC Plant Biol. 2007 Jul 14;7:38. doi: 10.1186/1471-2229-7-38 (PMC1947982; doi:10.1186/1471-2229-7-38)
Supplement: Additional file 4 — Cotyledon wp RT-PCR cDNA derived amino acid sequences and open reading frames. [file 1471-2229-7-38-S4.doc]

Cotyledon *wp* – cDNA clones derived amino acid sequences.

***wp*-9c**

>/tmp/outseq.input.9407 [Unknown form], frame+3, 994 bases, 7BC checksum.

IAFCYLIPLRTRTFSSKTTMAPTAKTLTYLAQEKTLESSFVRDEEERPKV

AYNEFSDEIPVISLAGIDEVDGRRREICEKIVEACENWGIFQVVDHGVDQ

QLVAEMTRLAKEFFALPPDEKLRFDMSGAKKGGFIVSSHLQGESVQDWRE

IVTYFSYPKRERDYSRWPDTPEGWRSVTEEYSDKVMGLACKLMEVLSEAM

GLEKEGLSKACVDMDQKVVVNYYPKCPQPDLTLGLKRHTDPGTITLLLQD

QVGGLQATRDNGKTWITVQPVEAAFVVNLGDHAHVDHDGIFVIRRLYSKT

SVIRKRRC*FRKKKVIRVLNPKQGHNLEAEFFLLCCINSYTTTMTVT*NH

L*RNPIPHPPPVPPRAASKRISPCSPTTKKKKM*LPVSEMISQS**QVQE

RDLQNLRGSL*LLTSQIVIFLFSHLLFLFSLR*LLELKELL*WNSTKRLR

R*GRDRVTNETIALKKIRLEQEDEGVPSTAIHEISLLKEMQHRNIVRL*D

VVHDEKSLYLVQLVSEGRQR*LDNKFKDFVARLKWLRLENFIQRSLPSLL

ESVSNQNGLLLYGLVHCNEQF*QPLQLLDFPRYNGVHLMR*TQGCVMVWH

MQKSRKTCQRSMSRFADL*FFSYLQVACN*YGR*KEAQCETSLLLIFMIH

RLKYDMS*TNIN*DLVTLDYMTRNHIIFPLDLFLKLF*KLFLVREFFLFI

FSSLIIFCLGFFVFLANLSMSSFHLYI*CLFIFLL*VTIQSQSCQLQYQV

YRNGNPYGIAEGIVFSMPCRSKVITKSKELVMVSFQVYWMLIY*MKLFY*

MKLFSI*AMEGSRMLITKRW*TQTIAVCP*PLFKTQHQMQLFTL*R*EKE

RSL*WRNQSLLLKCTGGR*ARTLRLQG*RSWLRKSICRTLRMKSICKNLI

RRQNLRPGL*RRFLLN***LHMYHLHAPLVFLVFFKGHELIIVLTFVLLY

VL*FILCGDIMCCVQLPMSY*LAGSSMYTLYLPLL*MKISGTVF

**(425 bp Exon 1, 429 bp Exon 2, & 74 bp UP Intron)**

>/tmp/outseq.input.9407 [Unknown form], frame+2, 994 bases, 261B checksum.

HCILLFNSTTYTHILLKDNNGTNSQDSDLPGPRENPRIELRSGRGGASQG

CLQRIQRRDPSDFSCRNRRGGWTQKRDL*EDRGGLRELGYIPGC*SRCGS

TTRGRDDPSRQRVLCFATGREASF*YVRRQKGWIHCLQPSPRGIGAGLER

NSDILFVPKKREGLFKVARHARRVEIGD*GIQRQSNGSSLQAHGGVVRSN

GVRERGFKQSMC*HGPEGGG*LLPQMPST*PHSWPEAPHGSGHYHLAASG

PSGWTSSHQGQWQNMDHRSACGGCLRRQSWRSCSCRPRWHFCNSKTLF*D

ECH*KTSLLV*KKKSYTRSEP*ARSQPRS*VLSTVLHQQLYDDNDCDLKS

PLKKPDSASTPSPTACGVKEDFSVLTNDEEEEDVIAGIRNDFTELVASSR

TGSSKSARLTVTPNFPDCHLPLQSSTFPLFSPMIVGTQRASLMEQYEKVE

KIGSRPRHQRDHRVEEDSPRAGG*GGSQHRHSRDFSLERNAAQEHC*VVG

CSAR*EEFVSGTIGF*GKTKVVR**IQGLCCKVEMVEAGELYSKKLAKFV

GKRLKSEWAASIWTSTLQRTILTATPIIGFPKIQWRALDEINAGVCDGMA

YAEIKKNMPEEYE*VCRLVIFLLFASCMQLIWQIKRSTM*NFSLINFYDT

*IKI*YVLDKY*LRPCNSRLYDQEPYNISFGFIP*TFLKIVFSS*IFFIY

F*FFNYILSWLFCFSCKFKHVQLSFIHLMSFYFLIVSYYSITKLPIAIPG

I*ERKSLWNS*RYCFQYAMPIKGDHQI*GIGNGLIPSVLDVNLLDEVILL

DEVIQYLSNGRFKNADHQAVVNSNHSRLSIATFQNPAPNATVYPLKIREG

EKPVMEEPITFAEMYRRKMSKDIEIARMKKLAKEKHLQDLENEKHLQELD

QKAKLEARPLKEILA*LIIITYVSFACPLGVFSIF*GP*INNSPYLCAFV

RLMIYPLWGYHVLCSVAYVLLASWLIYVYLIFASIINENKWHCL

**(28 bp UP Intron, 210 bp UP, & 192 bp CDC2)**

**(81 bp CS & 329 bp Exon 3)**

>/tmp/outseq.input.9407 [Unknown form], frame-1, 994 bases, 2026 checksum.

KDSATYFHL**RQI*GIHR*AS*LIRHRQLNTTHDIPTKDKS*DVQKHKG

KDYY*FMALKKY*KHQGGMQMIHM*LLLIKQESPSKAWPQVLPSDQVLAN

AFHSQGPANAFP*PASSSLQSQCPCSSSSCTFQQK*LVPPSQASLLLLSS

EGKQLHLVLGFEKWLWTNGYGLSSPPLGDQHS*TFHCSDTE*LHLVE*LH

LVD*HPVHLE*DHYQFLRFGDHL*SAWHTENNTFSYSIRISVPIYLVLQL

ATL*LNSNSQ*ENKKTSNV*MKAGHA*ICKKNKKAKTKYN*RTKNK*KKF

TN*KQFLKKFKE*IQRKYYMVPGHII*SYKVLVNICLRHIIF*SMYHKN*

*ERSFTLCFFLSAILVACNLQIREKLQVCKPTHTPLACFS*FLHMPYHHT

PLRLSHQVHAIVSWEIQ*LEWLSELFVAVY*SI*KQPILI*DAFQQTWQA

SLNKVLQPQPFQPCNKVLEFII*LPLSSLRNQLYQIQTLLIVHYILQPNN

VPVLHFFQERNLVNGGAGNPLILLLEANLLQRDGLVGDAVATLSSQPFRT

VPSKKLFEFQQSSERKEEK*MTEEEDDNLGS*ELQ*ASQILKIPFLNLLL

TL*NHFGYRQLHLLLLRRW*ARRNPL*RRTRWDWGWMRNRVSSEVILGHS

HCRRITVDAAQ*KELSFEVVTLLRVQNAYNFFFSKLTTTFSNDTRLRIKS

SNYKNAIVVYMSMISKIDDEGSLHRLNGDPCFAIVPGGLKSTHLVLKQQG

DSARIRVALQAKSEVRLRAFGVVINHHLLVHVNTCFA*TLFL*PHCFGQH

LHELAS*THYFVAVFLSHRSPPFWRVWPP*IVPLSFWVRKVCHYFSPVLH

RFPLEMAGDNESTLFGAGHIKTKLLVRWQSKELFGETGHLGHELLIHTVI

NNLEYTPILASLHDLLTNLSSASIHLVDSGKRNHWDLVAEFVVGNLGTLL

LVPNEARF*GFLLGQVSQSLGCWCHCCL*GECACT*WN*IAECN

**(145 bp UP Intron & 230 bp Exon 2)**

**(70 bp Exon 2 & 314 Exon 1)**

>/tmp/outseq.input.9407 [Unknown form], frame-3, 994 bases, C1D checksum.

RQCHLFSFIIEANIRYT*MSQLANKT*ATEHNT*YPHKG*IIRRTKAQR*

GLLLIHGP*KILKTPRGHANDTYVIIIN*ARISFKGLASSFAF*SSSCKC

FSFSRSCKCFSLASFFILAISMSLLIFLLYISAKVIGSSITGFSPSLIFR

G*TVAFGAGF*KVAMDKRLWFEFTTAW*SAFLNLPLLRY*ITSSSRITSS

SRLTSSTLGMRPLPIP*IW*SPLIGMAY*KQYLQLFHKDFRSYIPGIAIG

NFVIE**LTIRK*KDIKCINESWTCLNLQEKQKSQDKI*LKN*K*IKKIH

ELKTIFKKV*GINPKEILYGSWSYNLELQGLS*YLSKTYHILIYVS*KLI

REKFHIVLLFICHISCMQLANKRKITSLQTYSYSSGMFFLISAYAIPSHT

PAFISSSARHCILGNPIIGVAVRIVRCSVLVHIEAAHSDLRRFPTNLASF

FE*SSPASTISTLQQSP*IYYLTTFVFPQKPIVPDTNSSHRALHPTT*QC

SCAAFLSREKSREWRCWEPPHPPARGESSSTRWSRW*RGRDPIFSTFSYC

SIKEAL*VPTIIGEKRGKVDD*RGR*QSGKLGVTVSLADFEDPVLELATN

SVKSFRIPAITSSSSSSLVSTEKSSLTPHAVGLGVDAESGFFRGDFRSQS

LSSYNC*CSTVERTQLRGCDLA*GSERV*LFFF*TNNDVF**HSS*NKVF

ELQKCHRGLHEHDLQD*RRRQPPQAER*SMFCHCPWWLEVHPLGPEAAR*

*CPDPCGASGQE*GQVEGIWGSN*PPPSGPCQHMLCLNPLSLTPLLRTTP

P*ACKLDPLLCRCIPQSPISTLLACLATLNSPSLFLGTKSMSLFLSSPAP

IPLGDGWRQ*IHPFWRRTYQNEASRPVAKQRTLWRDGSSRPRVVDPHRDQ

QPGIYPNSRKPPRSSHKSLFCVHPPRRFRQEKSLGSRR*IRCRQPWDAPP

RPERSSILGFSLGPGKSESWLLVPLLSLRRMCVYVVELNSRMQC

**(103 bp FPK-MDH Intron & 212 bp FPK)**

***wp*-8c**

>/tmp/outseq.input.944 [Unknown form], frame+3, 992 bases, 1A3E checksum.

IAFCYLIPLRTRTFSSKTTMAPTAKTLAYLAQEKTLESSFVRDEEERPKV

AYNEFSDEIPVISLAGIDEVDGRRREICEKIVEACENWGIFQVVDHGVDQ

QLVAEMTRLAKEFFALPPDEKLRFDMSGAKKGGFIVSSHLQGESVQDWRE

IVTYFSYPKRERDYSRWPDTPEGWRSVTEEYSDKVMGLACKLMEVLSEAM

GLEKEGLSKACVDMDQKVVVNYYPKCPQPDLTLGLKRHTDPGTITLLLQD

QVGGLQATRDNGKTWITVQPVEAAFVVNLGDHAHTTMAFL*FEDFILRRV

SLENVVVSLEKKKLYAF*TLSKVTTSKLSSFYCAASTVIRRQ*L*PKITS

EETRFRIHPQSHRVRRQRGFLRAHQRRRRRRCNCRYPK*FHRVSSKFKNG

IFKICEAHCNS*LPRLSSSSSVIYFSSFLSDDCWNSKSFFDGTVRKG*ED

RVATASPTRPSR*RRFASSRRMRGFPAPPFTRFLS*KKCSTGTLLGCRM*

CTMRRVCIWYNWFLREDKGS*IINSRTLLQG*NG*GWRTLFKEACQVCWK

ASQIRMGCFYMD*YTATNNSDSHSNYWISQDTMACT**DKRRGV*WYGIC

RNQEKHARGV*VGLQTCNFSLICKLHATNMADKKKHNVKLLSY*FL*YID

*NIICLRQILTKTL*L*II*PGTI*YFFGFIP*TFLKIVFSS*IFFIYF*

FFNYILSWLFCFSCKFKHVQLSFIHLMSFYFLIVSYYSITKLPIAIPGI*

ERKSLWNS*RYCFQYAMPIKGDHQI*GIGNGLIPSVLDVNLLDEVILLDE

VIQYLSNGRFKNADHQAVVNSNHSRLSIATFQNPAPNATVYPLKIREGEK

PVMEEPITFAEMYRRKMSKDIEIARMKKLAKEKHLQDLENEKHLQELDQK

AKLEAKPLKEILA*LIIITYVSFACPLGVFSIF*GP*INNSPYLCAFVRL

MIYPLWGYHVLCSVAYVLLASWLIYVYLIFASIINENKWHCL

**(425 bp Exon 1, 429 bp Exon 2, & 18 bp UP Intron)**

**(81 bp CS & 329 bp Exon 3)**

>/tmp/outseq.input.944 [Unknown form], frame+1, 993 bases, ED9 checksum.

ALHSAI*FHYVHAHSPQRQQWHQQPRLWLTWPRRKP*NRASFGTRRSVPR

LPTTNSATRSQ*FLLPESTRWMDAEERFVRRSWRLARIGAYSRLLITVWI

NNSWPR*PVSPKSSLLCHRTRSFVLICPAPKRVDSLSPAISKGNRCRTGE

K**HTFRTQKERGTIQGGQTRQKGGGR*LRNTATK*WV*LASSWRCCPKQ

WG*RKRV*AKHVLTWTRRWWLITTPNALNLTSLLA*SATRIRALSPCCFR

TKWVDFKPPGTMAKHGSPFSLWRLPSSSILEIMLIPRWHFCNSKTLF*DE

CH*KTSLLV*KKKSYTRSEP*ARSQPRS*VLSTVLHQQLYDDNDCDLKSP

QKKPDSASTPSPTACGVKEDFSVLTNDEEEEDVIAGIRNDFTELVASSRT

GSSKSARLTVTPNFPDCHLPLQSSTFPLFSPMIVGTQRASLMEQYEKVEK

IGSRPRHQRDHRVEEDSPRAGG*GGSQHRRSRDFSLERNAAQEHC*VVGC

SAR*EEFVSGTIGF*GKTKVVR**IQGLCCKVEMVEAGELYSKKLAKFVG

KRLKSEWAASIWTSTLQRTILTATPIIGFPKIQWRALDEINAGVCDGMAY

AEIKKNMPEEYE*VCRLVIFLLFASCMQLIWQIKRSTM*NFSLINFYDT*

IKI*YVLDKY*LRPCNSRLYDQEPYNISLDLFLKLF*KLFLVREFFLFIF

SSLIIFCLGFFVFLANLSMSSFHLYI*CLFIFLL*VTIQSQSCQLQYQVY

RNGNPYGIAEGIVFSMPCRSKVITKSKELVMVSFQVYWMLIY*MKLFY*M

KLFSI*AMEGSRMLITKRW*TQTIAVCP*PLFKTQHQMQLFTL*R*EKER

SL*WRNQSLLLKCTGGR*ARTLRLQG*RSWLRKSICRTLRMKSICKNLIR

RQNLRPSL*RRFLLN***LHMYHLHAPLVFLVFFKGHELIIVLTFVLLYV

L*FILCGDIMCCVQLPMSY*LAGSSMYTLYLPLL*MKISGTVF

**(28 bp UP Intron, 210 bp UP, & 192 bp CDC2)**

>/tmp/outseq.input.944 [Unknown form], frame-2, 992 bases, 15B6 checksum.

KTVPLIFIYNRGKYKVYIDEPAS**DIGN*TQHMISPQRINHKTYKSTKV

RTIINSWPLKNTKNTKGACK*YICNYY*LSKNLLQRLGLKFCLLIKFLQM

LFILKVLQMLFLSQLLHPCNLNVLAHLPPVHFSKSDWFLHHRLLSFSYLQ

RVNSCIWCWVLKSGYGQTAMV*VHHRLVISILEPSIAQILNNFI**NNFI

**INIQYTWNETITNSLDLVITFDRHGILKTIPSAIP*GFPFLYTWYCNW

QLCD*IVTHNKKIKRHQMYK*KLDMLKFARKTKKPRQNIIKELKINKKNS

RTKNNF*KSLRNKSKEILYGSWSYNLELQGLS*YLSKTYYILIYVS*KLI

REKFHIVLLFICHISCMQLANKRKITSLQTYSYSSGMFFLISAYAIPSHT

PAFISSSARHCILGNPIIGVAVRIVRCSVLVHIEAAHSDLRRFPTNLASF

FE*SSPASTISTLQQSP*IYYLTTFVFPQKPIVPDTNSSHRALHPTT*QC

SCAAFLSREKSRERRCWEPPHPPARGESSSTRWSRW*RGRDPIFSTFSYC

SIKEAL*VPTIIGEKRGKVDD*RGR*QSGKLGVTVSLADFEDPVLELATN

SVKSFRIPAITSSSSSSLVSTEKSSLTPHAVGLGVDAESGFF*GDFRSQS

LSSYNC*CSTVERTQLRGCDLA*GSERV*LFFF*TNNDVF**HSS*NKVF

ELQKCHRGMSMISKIDDEGSLHRLNGDPCFAIVPGGLKSTHLVLKQQGDS

ARIRVALQAKSEVRLRAFGVVINHHLLVHVNTCFA*TLFL*PHCFGQHLH

ELAS*THYFVAVFLSHRPPPFWRVWPP*IVPLSFWVRKVCHYFSPVLHRF

PLEMAGDNESTLFGAGHIKTKLLVRWQSKELFGETGHLGHELLIHTVINN

LEYAPILASLHDLLTNLSSASIHLVDSGKRNHWDLVAEFVVGNLGTLLLV

PNEARF*GFLLGQVSQSLGCWCHCCL*GECACT*WN*IAECN

**(103 bp FPK-MDH Intron, & 212 bp FPK)**

**(70 bp Exon 2 & 314 Exon 1)**

***wp*-2c**

>/tmp/outseq.input.17602 [Unknown form], frame+3, 858 bases, 226A checksum.

IAFCYLIPLRTRTFSSKTTMAPTAKTLTYLAQEKTLESSFVRDEEERPKV

AYNEFSDEIPVISLAGIDEVDGRRREICEKIVEACENWGIFQVVDHGVDQ

QLVAEMTRLAKEFFALPPDEKLRFDMSGAKKGGFIVSSHLQGESVQDWRE

IVTYFSYPKRERDYSRWPDTPEGWRSVTEEYSDKVMGLACKLMEVLSEAM

GLEKEGLSKACVDMDQKVVVNYYPKCPQPDLTLGLKRHTDPGTITLLLQD

QVGGLQATRDNGKTWITVQPVEAAFVVNLGDHAHVDHDGIFVIRRLYSKT

SVIRKRRC*FRKKKVIRVLNPKQGHNLEAEFFLLCCINSYTTTMTVT*NH

L*RNPIPHPPPVPPRAASKRISPCSPTTKKKKM*LPVSEMISQS**QVQE

RDLQNLRGSL*LLTSQIVIFLFSHLLFLFSLR*LLELKELL*WNSTKRLR

R*GRDRVTNETIALKKIRLEQEDEGVPSTAIHEISLLKEMQHRNIVRL*D

VVHDEKSLYLVQLVSEGKQR*LDNKFKDFVARLKWLRLENFIQRSLPSLL

ESVSNQNGLLLYGLVHCNEQF*QPLQLLDFPRYNGVHLMR*TQGCVMVWH

MQKSRKTCQRSMSRYIGTEILME*LKVLFSVCHADQR*SPNLRNW*WSHS

KCTGC*STR*SYSTR*SYSVSEQWKVQEC*SPSGGELKP*PFVHSHFSKP

STKCNCLPSEDKRRREACDGGTNHFC*NVQEEDEQGH*DCKDEEAG*GKA

FAGP*E*KAFART*SEGKT*GQAFEGDSCLINNNYICIICMPPWCF*YFL

RAMN***SLPLCFCTSYDLSFVGISCVVFSCLCLIS*LAHLCIPYICLYY

K*K*VALS

**(425 bp Exon 1, 429 bp Exon 2, & 74 bp UP Intron)**

>/tmp/outseq.input.17602 [Unknown form], frame+2, 859 bases, 2180 checksum.

HCILLFNSTTYTHILLKDNNGTNSQDSDLPGPGENPRIELRSGRGGASQG

CLQRIQRRDPSDFSCRNRRGGWTQKRDL*EDRGGLRELGYIPGC*SRCGS

TTRGRDDPSRQRVLCFATGREASF*YVRRQKGWIHCLQPSPRGIGAGLER

NSDILFVPKKREGLFKVARHARRVEIGD*GIQRQSNGSSLQAHGGVVRSN

GVRERGFKQSMC*HGPEGGG*LLPQMPST*PHSWPEAPHGSGHYHLAASG

PSGWTSSHQGQWQNMDHRSACGGCLRRQSWRSCSCRPRWHFCNSKTLF*D

ECH*KTSLLV*KKKSYTRSEP*ARSQPRS*VLSTVLHQQLYDDNDCDLKS

PLKKPDSASTPSPTACGVKEDFSVLTNDEEEEDVIAGIRNDFTELVASSR

TGSSKSARLTVTPNFPDCHLPLQSSTFPLFSPMIVGTQRASLMEQYEKVE

KIGSRPRHQRDHRVEEDSPRAGG*GGSQHRHSRDFSLERNAAQEHC*VVG

CSAR*EEFVSGTIGF*GKTKVVR**IQGLCCKVEMVEAGELYSKKLAKFV

GKRLKSEWAASIWTSTLQRTILTATPIIGFPKIQWRALDEINAGVCDGMA

YAEIKKNMPEEYE*VYRNGNPYGIAEGIVFSMPCRSKVITKSKELVMVSF

QVYWMLIY*MKLFY*MKLFSI*AMEGSRMLITKRW*TQTIAVCP*PLFKT

QHQMQLFTL*R*EKERSL*WRNQSLLLKCTGGR*ARTLRLQG*RSWLRKS

ICRTLRMKSICKNLIRRQNLRPSL*RRFLLN***LHMYHLHAPLVFLVFF

KGHELIIVLTFVLLYVL*FILCGDIMCCVQLPMSY*LAGSSMYTLYLPLL

*MKISGTVF

**(28 bp UP Intron, 210 bp UP, & 192 bp CDC2)**

>/tmp/outseq.input.17602 [Unknown form], frame+1, 859 bases, 113E checksum.

ALHSAI*FHYVHAHSPQRQQWHQQPRL*LTWPRRKP*NRASFGTRRSVPR

LPTTNSATRSQ*FLLPESTRWMDAEERFVRRSWRLARIGVYSRLLITVWI

NNSWPR*PVSPKSSLLCHRTRSFVLICPAPKRVDSLSPAISKGNRCRTGE

K**HTFRTQKERGTIQGGQTRQKGGDR*LRNTATK*WV*LASSWRCCPKQ

WG*RKRV*AKHVLTWTRRWWLITTPNALNLTSLLA*SATRIRALSPCCFR

TKWVDFKPPGTMAKHGSPFSLWRLPSSSILEIMLM*TTMAFL*FEDFILR

RVSLENVVVSLEKKKLYAF*TLSKVTTSKLSSFYCAASTVIRRQ*L*PKI

TSEETRFRIHPQSHRVRRQRGFLRAHQRRRRRRCNCRYPK*FHRVSSKFK

NGIFKICEAHCNS*LPRLSSSSSVIYFSSFLSDDCWNSKSFFDGTVRKG*

EDRVATASPTRPSR*RRFASSRRMRGFPAPPFTRFLS*KKCSTGTLLGCR

M*CTMRRVCIWYNWFLRENKGS*IINSRTLLQG*NG*GWRTLFKEACQVC

WKASQIRMGCFYMD*YTATNNSDSHSNYWISQDTMACT**DKRRGV*WYG

ICRNQEKHARGV*VGI*ERKSLWNS*RYCFQYAMPIKGDHQI*GIGNGLI

PSVLDVNLLDEVILLDEVIQYLSNGRFKNADHQAVVNSNHSRLSIATFQN

PAPNATVYPLKIREGEKPVMEEPITFAEMYRRKMSKDIEIARMKKLAKEK

HLQDLENEKHLQELDQKAKLEAKPLKEILA*LIIITYVSFACPLGVFSIF

*GP*INNSPYLCAFVRLMIYPLWGYHVLCSVAYVLLASWLIYVYLIFASI

INENKWHCL

**(81 bp CS & 329 bp Exon 3)**

>/tmp/outseq.input.17602 [Unknown form], frame-3, 858 bases, 1C26 checksum.

RQCHLFSFIIEANIRYT*MSQLANKT*ATEHNT*YPHKG*IIRRTKAQR*

GLLLIHGP*KILKTPRGHANDTYVIIIN*ARISFKGLASSFAF*SSSCKC

FSFSRSCKCFSLASFFILAISMSLLIFLLYISAKVIGSSITGFSPSLIFR

G*TVAFGAGF*KVAMDKRLWFEFTTAW*SAFLNLPLLRY*ITSSSRITSS

SRLTSSTLGMRPLPIP*IW*SPLIGMAY*KQYLQLFHKDFRSYIPTHTPL

ACFS*FLHMPYHHTPLRLSHQVHAIVSWEIQ*LEWLSELFVAVY*SI*KQ

PILI*DAFQQTWQASLNKVLQPQPFQPCNKVLEFII*LPLFSLRNQLYQI

QTLLIVHYILQPNNVPVLHFFQERNLVNGGAGNPLILLLEANLLQRDGLV

GDAVATLSSQPFRTVPSKKLFEFQQSSERKEEK*MTEEEDDNLGS*ELQ*

ASQILKIPFLNLLLTL*NHFGYRQLHLLLLRRW*ARRNPL*RRTRWDWGW

MRNRVSSEVILGHSHCRRITVDAAQ*KELSFEVVTLLRVQNAYNFFFSKL

TTTFSNDTRLRIKSSNYKNAIVVYMSMISKIDDEGSLHRLNGDPCFAIVP

GGLKSTHLVLKQQGDSARIRVALQAKSEVRLRAFGVVINHHLLVHVNTCF

A*TLFL*PHCFGQHLHELAS*THYFVAVFLSHRSPPFWRVWPP*IVPLSF

WVRKVCHYFSPVLHRFPLEMAGDNESTLFGAGHIKTKLLVRWQSKELFGE

TGHLGHELLIHTVINNLEYTPILASLHDLLTNLSSASIHLVDSGKRNHWD

LVAEFVVGNLGTLLLVPNEARF*GFLLGQVSQSLGCWCHCCL*GECACT*

WN*IAECN

**(145 bp UP Intron & 230 bp UP Exon 2)**

**(70 bp Exon 2 & 314 Exon 1)**

***wp*-13c**

>/tmp/outseq.input.18595 [Unknown form], frame+3, 846 bases, 2405 checksum.

IAFCYLIPLRTRTFSSKTTMAPTAKTLTYLAQEKTLESSFVRDEEERPKV

AYNEFSDEIPVISLAGIDEVDGRRREICEKIVEACENWGIFQVVDHGVDQ

QLVAEMTRLAKEFFALPPDEKLRFDMSGAKKGGFIVSSHLQGESVQDWRE

IVTYFSYPKRERDYSRWPDAPEGWRSVTEEYSDKVMGLACKLMEVLSEAM

GLEKEGLSKACVDMDQKVVVNYYPKCPQPDLTLGLKRHTDPGTITLLLQD

QVGGLQATRDNGKIWITVQPVEAAFVVNLGDHAHLYDDNDCDLKSPLKKP

DSASTPSPTACGVKEDFSVLTNDEEEEDVIAGIRNDFTELVASSRTGSSK

SARLTVTPNFPDCHLPLQSSTFPLFSPMIVGTQRASLMEQYEKVEKIGSR

PRHQRDHRVEEDSPRAGG*GGSQHRHSRDFSLERNAAQEHC*VVGCSAR*

EEFVSGTIGF*GKTKVVR**IQGLCCKVEMV**F*LLYP*ILASLLSFLM

*YVALESFSIFG*IPRTLCNLL*Q*GWRTLFKEACQVCWKASQIRMGCFY

MD*YTATNNSDSHSNYWISQDTMACT**DKRRGV*WYGICRNQEKHARGV

*VYRNGNPYGIAEGIVFSMPCRSKVITKSKELVMVSFQVYWMLIY*MKLF

Y*MKLFSI*AMEGSRMLITKRW*TQTIAVCP*PLFKTQHQMQLFTL*R*E

KERSL*WRNQSLLLKCTGGR*ARTLRLQG*RSWLRKSICRTLRMKSICKN

LIRRQNLRPSL*RRFLLN***LHMYHLHAPLVFLVFFKGHELIIVLTFVL

LYVL*FILCGDIMCCVQLPMSY*LAGSSMYTLYLPLL*MKISGTVF

**(425 bp Exon 1, 429 bp Exon 2, 209 bp UP, & 192 bp CDC2)**

>/tmp/outseq.input.18595 [Unknown form], frame+2, 846 bases, 7AD checksum.

HCILLFNSTTYTHILLKDNNGTNSQDSDLPGPGENPRIELRSGRGGASQG

CLQRIQRRDPSDFSCRNRRGGWTQKRDL*EDRGGLRELGYIPGC*SRCGS

TTRGRDDPSRQRVLCFATGREASF*YVRRQKGWIHCLQPSPRGIGAGLER

NSDILFVPKKREGLFKVARRARRVEIGD*GIQRQSNGSSLQAHGGVVRSN

GVRERGFKQSMC*HGPEGGG*LLPQMPST*PHSWPEAPHGSGHYHLAASG

PSGWTSSHQGQWQNMDHRSACGGCLRRQSWRSCSFIRRQ*L*PKITSEET

RFRIHPQSHRVRRQRGFLRAHQRRRRRRCNCRYPK*FHRVSSKFKNGIFK

ICEAHCNS*LPRLSSSSSVIYFSSFLSDDCWNSKSFFDGTVRKG*EDRVA

TASPTRPSR*RRFASSRRMRGFPAPPFTRFLS*KKCSTGTLLGCRM*CTM

RRVCIWYNWFLREDKGS*IINSRTLLQG*NGVIVLTSLSLNSCFITLFSN

VICGIRIIFNIWLNT*NTMQFVITVRLENFIQRSLPSLLESVSNQNGLLL

YGLVHCNEQF*QPLQLLDFPRYNGVHLMR*TQGCVMVWHMQKSRKTCQRS

MSI*ERKSLWNS*RYCFQYAMPIKGDHQI*GIGNGLIPSVLDVNLLDEVI

LLDEVIQYLSNGRFKNADHQAVVNSNHSRLSIATFQNPAPNATVYPLKIR

EGEKPVMEEPITFAEMYRRKMSKDIEIARMKKLAKEKHLQDLENEKHLQE

LDQKAKLEAKPLKEILA*LIIITYVSFACPLGVFSIF*GP*INNSPYLCA

FVRLMIYPLWGYHVLCSVAYVLLASWLIYVYLIFASIINENKWHCL

**(81 bp CS & 329 bp Exon 3)**

>/tmp/outseq.input.18595 [Unknown form], frame+1, 846 bases, 252A checksum.

ALHSAI*FHYVHAHSPQRQQWHQQPRL*LTWPRRKP*NRASFGTRRSVPR

LPTTNSATRSQ*FLLPESTRWMDAEERFVRRSWRLARIGVYSRLLITVWI

NNSWPR*PVSPKSSLLCHRTRSFVLICPAPKRVDSLSPAISKGNRCRTGE

K**HTFRTQKERGTIQGGQTRQKGGDR*LRNTATK*WV*LASSWRCCPKQ

WG*RKRV*AKHVLTWTRRWWLITTPNALNLTSLLA*SATRIRALSPCCFR

TKWVDFKPPGTMAKYGSPFSLWRLPSSSILEIMLIYTTTMTVT*NHL*RN

PIPHPPPVPPRAASKRISPCSPTTKKKKM*LPVSEMISQS**QVQERDLQ

NLRGSL*LLTSQIVIFLFSHLLFLFSLR*LLELKELL*WNSTKRLRR*GR

DRVTNETIALKKIRLEQEDEGVPSTAIHEISLLKEMQHRNIVRL*DVVHD

EKSLYLVQLVSEGRQR*LDNKFKDFVARLKWCNSSNFSILEFLLHYSLF*

CDMWH*NHFQYLAEYLEHYAICYNSEAGELYSKKLAKFVGKRLKSEWAAS

IWTSTLQRTILTATPITGFPKIQWRALDEINAGVCDGMAYAEIKKNMPEE

YEYIGTEILME*LKVLFSVCHADQR*SPNLRNW*WSHSKCTGC*STR*SY

STR*SYSVSEQWKVQEC*SPSGGELKP*PFVHSHFSKPSTKCNCLPSEDK

RRREACDGGTNHFC*NVQEEDEQGH*DCKDEEAG*GKAFAGP*E*KAFAR

T*SEGKT*GQAFEGDSCLINNNYICIICMPPWCF*YFLRAMN***SLPLC

FCTSYDLSFVGISCVVFSCLCLIS*LAHLCIPYICLYYK*K*VALS

**(57 bp FPK Intron, 231 bp FPK, & 27 bp MDH)**

>/tmp/outseq.input.18595 [Unknown form], frame-1, 846 bases, 1E1F checksum.

KDSATYFHL**RQI*GIHR*AS*LIRHRQLNTTHDIPTKDKS*DVQKHKG

KDYY*FMALKKY*KHQGGMQMIHM*LLLIKQESPSKAWPQVLPSDQVLAN

AFHSQGPANAFP*PASSSLQSQCPCSSSSCTFQQK*LVPPSQASLLLLSS

EGKQLHLVLGFEKWLWTNGYGLSSPPLGDQHS*TFHCSDTE*LHLVE*LH

LVD*HPVHLE*DHYQFLRFGDHL*SAWHTENNTFSYSIRISVPIYSYSSG

MFFLISAYAIPSHTPAFISSSARHCILGNPVIGVAVRIVRCSVLVHIEAA

HSDLRRFPTNLASFFE*SSPASLL*QIA*CSRYSAKY*K*F*CHISH*KR

E**SKNSRIEKLELLHHFNLATKSLNLLSNYLCLPSETNCTRYKLFSSCT

TSYNLTMFLCCISFKREIS*MAVLGTPSSSCSRRIFFNAMVSLVTRSRPY

LLNLFVLFHQRSSLSSNNHRREKRKSR*LKRKMTIWEVRSYSEPRRF*RS

RS*TCY*LCEIISDTGNYIFFFFVVGEHGEILFDAARGGTGGGCGIGFLQ

R*F*VTVIVVV*MSMISKIDDEGSLHRLNGDPYFAIVPGGLKSTHLVLKQ

QGDSARIRVALQAKSEVRLRAFGVVINHHLLVHVNTCFA*TLFL*PHCFG

QHLHELAS*THYFVAVFLSHRSPPFWRVWPP*IVPLSFWVRKVCHYFSPV

LHRFPLEMAGDNESTLFGAGHIKTKLLVRWQSKELFGETGHLGHELLIHT

VINNLEYTPILASLHDLLTNLSSASIHLVDSGKRNHWDLVAEFVVGNLGT

LLLVPNEARF*GFLLGQVSQSLGCWCHCCL*GECACT*WN*IAECN

**(70 bp Exon 2 & 314 Exon 1)**

***wp*-12c**

>/tmp/outseq.input.24446 [Unknown form], frame+3, 802 bases, 1DB3 checksum.

IAFCYLIPLRTRTFSSKTTMAPTAKTLTYLAQEKTLESSFVRDEEERPKV

AYNEFSDEIPVISLAGIDEVDGRRREICEKIVEACENWGIFQVVDHGVDQ

QLVAEMTRLAKEFFALPPDEKLRFDMSGAKKGGFIVSSHLQGESVQDWRE

IVTYFSYPKERGTIQGGQTRQKGGDR*LRNTATK*WV*LASSWRCCPKQW

G*RKRV*AKHVLTWTRRWWLITTPNALNLTSLLA*SATRIRALSPCCFRT

KWVDFKPPGTMAKHGSPFSLWRLPSSSILEIMLIYTTTMTVT*NHL*RNP

IPHPPPVPPRAASKRISPCSPTTKKKKM*LPVSEMISQS**QVQERDLQN

LRGSL*LLTSQIVIFLFSHLLFLFSLR*LLELKELL*WNSTKRLRR*GRD

RVTNETIALKKIRLEQEDEGVPSTAIHEISLLKEMQHRNIVRL*DVVHDE

KSLYLVQLVSEGRQR*LDNKFKDFVARLKWLRLENFIQRSLPSLLESVSN

QNGLLLYGLVHCNEQF*QPLQLLDFPRYNGVHLMR*TQGCVMVWHMQKSR

KTCQRSMSI*ERKSLWNS*RYCFQYAMPIKGDHQI*GIGNGLIPSVLDVN

LLDEVILLDEVIQYLSNGRFKNADHQAVVNSNHSRLSIATFQNPAPNATV

YPLKIREGEKPVMEEPITFAEMYRRKMSKDIEIARMKKLAKEKHLQDLEN

EKHLQELDQKAKLEAKPLKEILA*LIIITYVSFACPLGVFSIF*GP*INN

SPYLCAFVRLMIYPLWGYHVLCSVAYVLLASWLIYVYLIFASIINENKWH

CL

**(425 bp Exon 1 & 104 bp Exon 2)**

**(81 bp CS & 329 bp Exon 3)**

>/tmp/outseq.input.24446 [Unknown form], frame+2, 802 bases, D50 checksum.

HCILLFNSTTYTHILLKDNNGTNSQDSDLPGPGENPRIELRSGRGGASQG

CLQRIQRRDPSDFSCRNRRGGWTQKRDL*EDRGGLRELGYIPGC*SRCGS

TTRGRDDPSRQRVLCFATGREASF*YVRRQKGWIHCLQPSPRGIGAGLER

NSDILFVPKRERDYSRWPDTPEGWRSVTEEYSDKVMGLACKLMEVLSEAM

GLEKEGLSKACVDMDQKVVVNYYPKCPQPDLTLGLKRHTDPGTITLLLQD

QVGGLQATRDNGKTWITVQPVEAAFVVNLGDHAHLYDDNDCDLKSPLKKP

DSASTPSPTACGVKEDFSVLTNDEEEEDVIAGIRNDFTELVASSRTGSSK

SARLTVTPNFPDCHLPLQSSTFPLFSPMIVGTQRASLMEQYEKVEKIGSR

PRHQRDHRVEEDSPRAGG*GGSQHRHSRDFSLERNAAQEHC*VVGCSAR*

EEFVSGTIGF*GKTKVVR**IQGLCCKVEMVEAGELYSKKLAKFVGKRLK

SEWAASIWTSTLQRTILTATPIIGFPKIQWRALDEINAGVCDGMAYAEIK

KNMPEEYEYIGTEILME*LKVLFSVCHADQR*SPNLRNW*WSHSKCTGC*

STR*SYSTR*SYSVSEQWKVQEC*SPSGGELKP*PFVHSHFSKPSTKCNC

LPSEDKRRREACDGGTNHFC*NVQEEDEQGH*DCKDEEAG*GKAFAGP*E

*KAFART*SEGKT*GQAFEGDSCLINNNYICIICMPPWCF*YFLRAMN**

*SLPLCFCTSYDLSFVGISCVVFSCLCLIS*LAHLCIPYICLYYK*K*VA

LS

**(50 bp Exon 1, 429 bp Exon 2, 209 bp UP, & 192 bp CDC2)**

>/tmp/outseq.input.24446 [Unknown form], frame-2, 802 bases, 5FD checksum.

KTVPLIFIYNRGKYKVYIDEPAS**DIGN*TQHMISPQRINHKTYKSTKV

RTIINSWPLKNTKNTKGACK*YICNYY*LSKNLLQRLGLKFCLLIKFLQM

LFILKVLQMLFLSQLLHPCNLNVLAHLPPVHFSKSDWFLHHRLLSFSYLQ

RVNSCIWCWVLKSGYGQTAMV*VHHRLVISILEPSIAQILNNFI**NNFI

**INIQYTWNETITNSLDLVITFDRHGILKTIPSAIP*GFPFLYTHTPLA

CFS*FLHMPYHHTPLRLSHQVHAIVSWEIQ*LEWLSELFVAVY*SI*KQP

ILI*DAFQQTWQASLNKVLQPQPFQPCNKVLEFII*LPLSSLRNQLYQIQ

TLLIVHYILQPNNVPVLHFFQERNLVNGGAGNPLILLLEANLLQRDGLVG

DAVATLSSQPFRTVPSKKLFEFQQSSERKEEK*MTEEEDDNLGS*ELQ*A

SQILKIPFLNLLLTL*NHFGYRQLHLLLLRRW*ARRNPL*RRTRWDWGWM

RNRVSSEVILGHSHCRRINEHDLQD*RRRQPPQAER*SMFCHCPWWLEVH

PLGPEAAR**CPDPCGASGQE*GQVEGIWGSN*PPPSGPCQHMLCLNPLS

LTPLLRTTPP*ACKLDPLLCRCIPQSPISTLLACLATLNSPSLFWVRKVC

HYFSPVLHRFPLEMAGDNESTLFGAGHIKTKLLVRWQSKELFGETGHLGH

ELLIHTVINNLEYTPILASLHDLLTNLSSASIHLVDSGKRNHWDLVAEFV

VGNLGTLLLVPNEARF*GFLLGQVSQSLGCWCHCCL*GECACT*WN*IAE

CN

**(151 bp Exon 2 & 314 bp Exon 1)**

***wp*-6c**

>/tmp/outseq.input.17873 [Unknown form], frame+3, 782 bases, 2D4 checksum.

IAFCYLIPLRTRTFSSKTTMAPTAKTLTYLAQEKTLESSFVRDEEERPKV

AYNEFSDEIPVISLAGIDEVDGRRREICGKIVEACENWGIFQVVDHGVDQ

QLVAEMTRLAKEFFALPPDEKLRFDMSGAKKGGFIVSSHLQDWREIVTYF

SYPKRERDYSRWPDTPEGWRSVTEEYSDKVMGLACKLMEVLSEAMGLEKE

GLSKACVDMDQKVVVNYYPKCPQPDLTLGLKRHTDPGTITLLLQDQVGGL

QATRDNGKTWITVQPVEAAFVVNLGDHAHLYDDNDCDLKSPLKEPDSAST

PSPTACGVKEDFSVLTNDEEEEDVIAGIRNDFTELVASSRTGSSKSARLT

VTPNFPDCHLPLQSSTFPLFSPMIVGTQRASLMEQYEKVEKIGSRPRHQR

DHRVEEDSPRAGG*GGSQHRHSRDFSLERNAAQEHC*VVGCSAR*EEFVS

GTIGF*GKTK*GWRTLFKEACQVCWKASQIRTGCFYMD*YTATNNSDSHS

NYWISQDTMACT**DKRRGV*WYGICRNQEKHARGV*VYRNGNPYGIAEG

IVFSMPCRSKVITKSKELVMVSFQVYWMLIY*MKLFY*MKLFSI*AMEGS

RMLITKRW*TQTIAVCP*PLFKTQHQMQLFTL*R*EKERSL*WRNQSLLL

KCTGGR*ARTLRLQG*RSWLRKSICRTLRMKSICKNLIRRQNLRPSL*RR

FLLN***LHMYHLHAPLVFLVFFKGHELIIVLTFVLLYVL*FILCGDIMC

CVQLPMSY*LAGSSMYTLYLPLL*MKISGTVF

**(425 bp Exon 1, 414 bp Exon 2, 209 bp UP, & 192 bp CDC2)**

>/tmp/outseq.input.17873 [Unknown form], frame+2, 782 bases, 2A0 checksum.

HCILLFNSTTYTHILLKDNNGTNSQDSDLPGPGENPRIELRSGRGGASQG

CLQRIQRRDPSDFSCRNRRGGWTQKRDLWEDRGGLRELGYIPGC*SRCGS

TTRGRDDPSRQRVLCFATGREASF*YVRRQKGWIHCLQPSPRLERNSDIL

FVPKKREGLFKVARHARRVEIGD*GIQRQSNGSSLQAHGGVVRSNGVRER

GFKQSMC*HGPEGGG*LLPQMPST*PHSWPEAPHGSGHYHLAASGPSGWT

SSHQGQWQNMDHRSACGGCLRRQSWRSCSFIRRQ*L*PKITSEGTRFRIH

PQSHRVRRQRGFLRAHQRRRRRRCNCRYPK*FHRVSSKFKNGIFKICEAH

CNS*LPRLSSSSSVIYFSSFLSDDCWNSKSFFDGTVRKG*EDRVATASPT

RPSR*RRFASSRRMRGFPAPPFTRFLS*KKCSTGTLLGCRM*CTMRRVCI

WYNWFLREDKVRLENFIQRSLPSLLESVSNQNGLLLYGLVHCNEQF*QPL

QLLDFPRYNGVHLMR*TQGCVMVWHMQKSRKTCQRSMSI*ERKSLWNS*R

YCFQYAMPIKGDHQI*GIGNGLIPSVLDVNLLDEVILLDEVIQYLSNGRF

KNADHQAVVNSNHSRLSIATFQNPAPNATVYPLKIREGEKPVMEEPITFA

EMYRRKMSKDIEIARMKKLAKEKHLQDLENEKHLQELDQKAKLEAKPLKE

ILA*LIIITYVSFACPLGVFSIF*GP*INNSPYLCAFVRLIIYPLWGYHV

LCSVAYVLLASWLIYVYLIFASIINENKWHCL

**(81 bp CS & 329 bp Exon 3)**

>/tmp/outseq.input.17873 [Unknown form], frame+1, 782 bases, 4C3 checksum.

ALHSAI*FHYVHAHSPQRQQWHQQPRL*LTWPRRKP*NRASFGTRRSVPR

LPTTNSATRSQ*FLLPESTRWMDAEERFVGRSWRLARIGVYSRLLITVWI

NNSWPR*PVSPKSSLLCHRTRSFVLICPAPKRVDSLSPAISKTGEK**HT

FRTQKERGTIQGGQTRQKGGDR*LRNTATK*WV*LASSWRCCPKQWG*RK

RV*AKHVLTWTRRWWLITTPNALNLTSLLA*SATRIRALSPCCFRTKWVD

FKPPGTMAKHGSPFSLWRLPSSSILEIMLIYTTTMTVT*NHL*RNPIPHP

PPVPPRAASKRISPCSPTTKKKKM*LPVSEMISQS**QVQERDLQNLRGS

L*LLTSQIVIFLFSHLLFLFSLR*LLELKELL*WNSTKRLRR*GRDRVTN

ETIALKKIRLEQEDEGVPSTAIHEISLLKEMQHRNIVRL*DVVHDEKSLY

LVQLVSEGRQSEAGELYSKKLAKFVGKRLKSERAASIWTSTLQRTILTAT

PIIGFPKIQWRALDEINAGVCDGMAYAEIKKNMPEEYEYIGTEILME*LK

VLFSVCHADQR*SPNLRNW*WSHSKCTGC*STR*SYSTR*SYSVSEQWKV

QEC*SPSGGELKP*PFVHSHFSKPSTKCNCLPSEDKRRREACDGGTNHFC

*NVQEEDEQGH*DCKDEEAG*GKAFAGP*E*KAFART*SEGKT*GQAFEG

DSCLINNNYICIICMPPWCF*YFLRAMN***SLPLCFCTSYNLSFVGISC

VVFSCLCLIS*LAHLCIPYICLYYK*K*VALS

**(95 bp CDC2, 199 bp FPK, & 27 bp MDH)**

>/tmp/outseq.input.17873 [Unknown form], frame-1, 782 bases, 192B checksum.

KDSATYFHL**RQI*GIHR*AS*LIRHRQLNTTHDIPTKDKL*DVQKHKG

KDYY*FMALKKY*KHQGGMQMIHM*LLLIKQESPSKAWPQVLPSDQVLAN

AFHSQGPANAFP*PASSSLQSQCPCSSSSCTFQQK*LVPPSQASLLLLSS

EGKQLHLVLGFEKWLWTNGYGLSSPPLGDQHS*TFHCSDTE*LHLVE*LH

LVD*HPVHLE*DHYQFLRFGDHL*SAWHTENNTFSYSIRISVPIYSYSSG

MFFLISAYAIPSHTPAFISSSARHCILGNPIIGVAVRIVRCSVLVHIEAA

RSDLRRFPTNLASFFE*SSPASLCLPSETNCTRYKLFSSCTTSYNLTMFL

CCISFKREIS*MAVLGTPSSSCSRRIFFNAMVSLVTRSRPYLLNLFVLFH

QRSSLSSNNHRREKRKSR*LKRKMTIWEVRSYSEPRRF*RSRS*TCY*LC

EIISDTGNYIFFFFVVGEHGEILFDAARGGTGGGCGIGFLQR*F*VTVIV

VV*MSMISKIDDEGSLHRLNGDPCFAIVPGGLKSTHLVLKQQGDSARIRV

ALQAKSEVRLRAFGVVINHHLLVHVNTCFA*TLFL*PHCFGQHLHELAS*

THYFVAVFLSHRSPPFWRVWPP*IVPLSFWVRKVCHYFSPVLEMAGDNES

TLFGAGHIKTKLLVRWQSKELFGETGHLGHELLIHTVINNLEYTPILASL

HDLPTNLSSASIHLVDSGKRNHWDLVAEFVVGNLGTLLLVPNEARF*GFL

LGQVSQSLGCWCHCCL*GECACT*WN*IAECN

**(63 bp MDH & 213 bp FPK)**

**(55 bp Exon 2 & 314 bp Exon 1)**

>/tmp/outseq.input.17873 [Unknown form], frame-3, 782 bases, 1DFC checksum.

RQCHLFSFIIEANIRYT*MSQLANKT*ATEHNT*YPHKG*IIRRTKAQR*

GLLLIHGP*KILKTPRGHANDTYVIIIN*ARISFKGLASSFAF*SSSCKC

FSFSRSCKCFSLASFFILAISMSLLIFLLYISAKVIGSSITGFSPSLIFR

G*TVAFGAGF*KVAMDKRLWFEFTTAW*SAFLNLPLLRY*ITSSSRITSS

SRLTSSTLGMRPLPIP*IW*SPLIGMAY*KQYLQLFHKDFRSYILILLWH

VFLDFCICHTITHPCVYLIKCTPLYLGKSNNWSGCQNCSLQCTSPYRSSP

F*FETLSNKLGKLL*IKFSSLTLSSLRNQLYQIQTLLIVHYILQPNNVPV

LHFFQERNLVNGGAGNPLILLLEANLLQRDGLVGDAVATLSSQPFRTVPS

KKLFEFQQSSERKEEK*MTEEEDDNLGS*ELQ*ASQILKIPFLNLLLTL*

NHFGYRQLHLLLLRRW*ARRNPL*RRTRWDWGWMRNRVPSEVILGHSHCR

RINEHDLQD*RRRQPPQAER*SMFCHCPWWLEVHPLGPEAAR**CPDPCG

ASGQE*GQVEGIWGSN*PPPSGPCQHMLCLNPLSLTPLLRTTPP*ACKLD

PLLCRCIPQSPISTLLACLATLNSPSLFLGTKSMSLFLSSLGDGWRQ*IH

PFWRRTYQNEASRPVAKQRTLWRDGSSRPRVVDPHRDQQPGIYPNSRKPP

RSSHKSLFCVHPPRRFRQEKSLGSRR*IRCRQPWDAPPRPERSSILGFSP

GPGKSESWLLVPLLSLRRMCVYVVELNSRMQC

**(20 bp FPK & 283 bp CDC2)**

** Exon 1, Exon 2, & Exon 3**

** UP Intron**

** UP**

** CDC2**

** FPK Intron**

** FPK**

** FPK-MDH Intron**

** MDH**

** CS**
